# Unveiling the Longevity Potential of Natural Phytochemicals: A Comprehensive Review of Active Ingredients in Dietary Plants and Herbs

**DOI:** 10.1021/acs.jafc.4c07756

**Published:** 2024-10-31

**Authors:** Yu Wang, Xiuling Cao, Jin Ma, Shenkui Liu, Xuejiao Jin, Beidong Liu

**Affiliations:** † State Key Laboratory of Subtropical Silviculture, School of Forestry and Biotechnology, Zhejiang A&F University, Hangzhou 311300, China; ‡ Department of Chemistry and Molecular Biology, University of Gothenburg, Gothenburg 41390, Sweden

**Keywords:** Phytochemicals, Aging, Longevity, Antiaging, Metabolism, Lipid metabolism

## Abstract

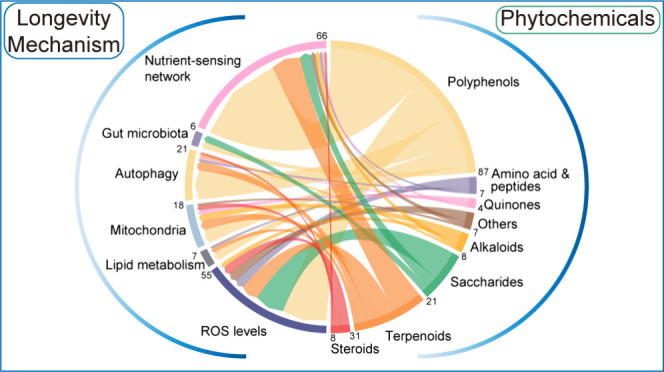

Ancient humans used dietary plants and herbs to treat disease and to pursue eternal life. Today, phytochemicals in dietary plants and herbs have been shown to be the active ingredients, some of which have antiaging and longevity-promoting effects. Here, we summarize 210 antiaging phytochemicals in dietary plants and herbs, systematically classify them into 8 groups. We found that all groups of phytochemicals can be categorized into six areas that regulate organism longevity: ROS levels, nutrient sensing network, mitochondria, autophagy, gut microbiota, and lipid metabolism. We review the role of these processes in aging and the molecular mechanism of the health benefits through phytochemical-mediated regulation. Among these, how phytochemicals promote longevity through the gut microbiota and lipid metabolism is rarely highlighted in the field. Our understanding of the mechanisms of phytochemicals based on the above six aspects may provide a theoretical basis for the further development of antiaging drugs and new insights into the promotion of human longevity.

## 1 Introduction

Aging is a cellular stress response triggered by molecular damage, which ultimately results in an imbalance within the body. This is an inevitable progression toward dysfunction and eventual death across most living organisms, particularly mammals. As aging progresses, there is accumulation of damage that increases disease susceptibility and mortality. Why and how we age remains a mystery. However, lifespan can be extended, and both caloric restriction and a plant-based diet are considered to play notable roles.^1^ Caloric restriction (CR) refers to the reduction in calorie intake without causing malnutrition. It can prolong the lifespan of various model organisms ranging from yeast to primates.^2−4^ Consistent with this notion, recent studies have demonstrated that healthful plant-based dietary patterns, which include whole grains, fruits, vegetables, nuts, legumes, and tea, are associated with a decreased risk of mortality among older adults.^5^ Higher plant protein intake carries a lower risk of all-cause mortality and cardiovascular diseases,^6^ which suggests that plant-based diets have an effect on longevity.

Numerous dietary plants and herbs, traditionally employed in ancient times to address a variety of diseases such as traditional Chinese medicines (TCMs), are now gaining recognition for their pharmacological effects. This is primarily due to the organic and bioactive compounds known as phytochemicals produced by these plants.^7,8^ Their roles in promoting longevity are of interest, as some phytochemicals have been found to increase the lifespan in a variety of model organisms, from yeast to mice.^9−11^ Although many phytochemicals have been identified to be associated with organismal health or longevity, the underlying mechanisms of action of most phytochemicals are not fully understood. In this review, we concentrate on phytochemicals whose antiaging properties have been extensively researched across various model organisms and/or validated in mammals. We also explore potential applications and suggest areas for future investigation.

We will discuss how these phytochemicals affect aging by regulation from six aspects: the reactive oxygen species (ROS) levels, nutrient-sensing network, mitochondria, autophagy, gut microbiota, and lipid metabolism. Notably, the first letter of all the phytochemicals’ names is capitalized throughout the text for easy identification.

## 2 Antiaging Phytochemicals

In this review, we summarized approximately 210 phytochemicals (Supplementary Table 1–8) that were reported to have effects on the aging process in different model systems, along with their sources, functions, and potential underlying mechanisms. These natural phytochemicals can be divided into eight categories according to their chemical structures, namely, saccharides, amino acids and peptides, quinones, polyphenols, terpenoids, steroids, alkaloids, and others.

### 2.1 Saccharides

Saccharides are primary metabolites synthesized by plants through photosynthesis and are widely distributed in nature. According to the number of monosaccharide groups constituting saccharides, they can be divided into monosaccharides, oligosaccharides, and polysaccharides.^12^ Saccharides and other compounds can form glycosides through glycosidic bonds. The bioactivities of many dietary plants and herbs are closely related to saccharides and their derivatives, such as polysaccharides and glycosides, many of which have antiaging activities.^13^

Among saccharides and their derivatives, *Astragalus* polysaccharides (APS) are the active ingredient of *Angelica sinensis* in TCMs, whose antiaging effects have been studied extensively and deeply by researchers. APS can reduce pathogenic polyglutamine (polyQ) aggregates in Huntington’s disease, alleviate the associated neurotoxicity, and extend the maximum lifespan of both wild-type and polyQ aggregate-containing *Caenorhabditis elegans*.^14^ Moreover, APS has been reported to activate antioxidant enzymes, such as superoxide dismutase (SOD) and catalase (CAT), to protect mitochondria by scavenging ROS in a D-gal-induced aging mouse model.^15^ It also balances endoplasmic reticulum (ER) homeostasis through the immune globulin binding protein–protein kinase R-like ER kinase (Bip-PERK) signaling pathway in *Bombyx mori*(16) and the insulin/IGF-1 signaling (IIS) pathway in *Drosophila melanogaster*, thereby extending the maximum lifespan of *B. mori* and *D. melanogaster*.^17^

Saccharides can readily interact with other small molecules (such as phenols, vitamins, and mineral elements), lipids, and proteins,^18^ thereby affecting the physiological functions of organisms.

### 2.2 Amino Acids and Peptides

At present, many amino acids and peptides have been isolated from dietary plants and show certain biological activities in organisms. There are two kinds of amino acids in nature, namely, protein amino acids and nonprotein amino acids (NPAAs). NPAAs are intermediates in protein amino acid biosynthesis, and their structures are similar to protein amino acids.^19^ According to reports, more than 250 NPAAs have been isolated from plants.^20^ Of these, L-Theanine is the only one studied for its antiaging effects (Supplementary Table 2), although others are also of interest. L-Theanine is known to be present in green tea (*Camellia sinensis*) and has many unique health benefits.^19^ It can increase worms’ maximum lifespan and resistance to oxidative stress, although the underlying mechanism is unclear.^21^

Certain amino acid derivatives demonstrate notable antiaging properties and serve as key active components in dietary supplements. Spermidine, primarily derived from arginine, can induce autophagy and promote longevity. It is prevalent in plant-based foods such as wheat germ, soybeans, and peas.^22,23^ Taurine, a cysteine derivative, is synthesized in abundance in humans and other eukaryotes. Ergothioneine, a sulfur-containing histidine derivative, is predominantly sourced from editable fungi and some prokaryotes. Both taurine and ergothioneine have been shown to promote mammals’ longevity and healthy aging.^24,25^

Peptides from dietary plants also have many pharmacological effects, including antiaging effects.^26^ Current studies on plant peptides usually utilize protein hydrolysates that contain many different types of peptides, and it would be more instructive to test the antiaging effects of a single peptide with a specific amino acid sequence. For instance, Tyr-Ala (TA), a single dipeptide isolated from hydrolyzed maize protein, can prolong the maximum lifespan of worms under normal conditions, heat, and oxidative stress by upregulating some antiaging associated genes.^27^ Some previous studies have shown that most antioxidant peptides have less than 20 amino acid residues,^28−32^ and whether their antioxidant abilities can be translated into antiaging effects may be a worthy research direction.

### 2.3 Quinones

Generally, natural organic compounds with unsaturated cyclic diketone structures or compounds that are easily converted to such structures are referred to as quinones. Quinones mainly include benzoquinone, naphthoquinone, anthraquinone, and phenanthrenequinone compounds, which are important secondary metabolites and widely present in nature.^33^ Quinones are regarded as privileged structures for designing new compounds with possible pharmacological activities, and their antitumor, antibacterial, and antiviral properties have been widely reported.^34^ However, research on their antiaging functions is limited.

Only four kinds of quinones have been found to have antiaging effects (Supplementary Table 3). Among them, Juglone and Plumbagin are naphthoquinones,^35,36^ Emodin is an anthraquinone,^37^ and Ehretiquinone is a benzoquinone-type molecule.^38^ Ehretiquinone isolated from *Onosma bracteatum* is the only one that has been studied in different models, including yeast, mammalian cells, and mice, while others have only been tested in worms. Ehretiquinone can prolong the replicative lifespan and the chronological lifespan of yeast as well as the yeast-like chronological lifespan of mammalian cells. It also promotes autophagy in mice, which may depend on the upregulation of the histone deacetylase Sir2 and increased activities of antioxidant enzymes.^38^ Juglone and Plumbagin are usually considered to be ROS generators that induce oxidative stress in *C. elegans*. They can prolong the lifespan of worms at a low dose through hormesis, which means that low levels of stress can trigger beneficial adaptive responses to protect organisms from subsequent severe stress.^35,36,39^ In addition, the naturally occurring anthraquinone Emodin, which is isolated from many TCMs, can extend the maximum lifespan of worms and protect worms from oxidative stress, depending on SIR-2.1 and abnormal dauer formation (DAF)-16.^37^

Further in-depth investigations on the antiaging effects of quinones are also needed, and due to their different structural modification possibilities, they may be candidates for the development of synthetic derivatives of antiaging drugs.

### 2.4 Polyphenols

The antiaging effects of polyphenols are the most widely and deeply studied among plant secondary metabolites. Furthermore, they are prevalent in our daily diet, with reported dietary intake reaching up to 1g per day.^40^ Such high doses may induce pharmacological effects to humans. Traditionally, plant polyphenols are defined as plant tannins and tannin-derived compounds, which can be structurally categorized into flavonoids, phenylpropanoids, phenolic acids, stilbenes, and other polyphenols with a hydroxyl group(s) attached to the carbon atom on the aromatic ring.^41^

Polyphenols are considered autophagy inducers^42,43^ and are the best-studied natural antioxidants.^44^ Many of them, such as Epigallocatechin-3-gallate, Resveratrol, and Curcumin, have shown antiaging activities, which are of interest and are under vigorous investigation. Here, we will not describe them in detail, as many researchers have conducted detailed and critical reviews.^45,46^ Instead, we introduce some emerging phytochemicals that have recently drawn attention for their roles in improving mammalian health and promoting longevity.

#### 2.4.1 Phenylpropanoids

Phenylpropanoids are a broad category of plant secondary metabolites, consisting of one or more C6–C3 structural unit acids derived from shikimic acids. Phenylpropanoids possess diverse pharmacological activities that are advantageous to human health.^47^ They contain many kinds of phytochemicals, such as phenylpropene, phenylpropanol, phenylpropionic acid and its condensations, coumarin, and lignan. Among them, the antiaging effects of phenylpropionic acids are the most widely and thoroughly studied. For instance, Caffeic acid and its derivatives, namely, Chlorogenic acid,^48,49^ Ferulic acid,^50^ Chicoric acid,^51^ and Rosmarinic acid,^52^ can promote the longevity of worms. The underlying mechanisms of lifespan extension include activation of the transcription factors DAF-16, heat shock factor (HSF)-1, and SKN-1 (a homologue of nuclear factor erythroid 2-related factor 2) and their downstream gene expression; activation of the adenosine monophosphate-activated protein kinase (AMPK) pathway; reduction of polyQ aggregate formation; and modulation of ROS levels and mitochondrial functions.

In addition to phenylpropionic acid, coumarin and lignan have also been reported to be involved in lifespan extension. Coumarin is a secondary metabolite widely present in plants and belongs to the benzopyrone family with diverse bioactivities.^53^ Ferulsinaic acid is a new rearranged class of sesquiterpene coumarin that can increase the maximum lifespan of worms and increase their resistance to heat stress and oxidative stress. The lifespan extension effect may partially depend on the expression of stress resistance-related genes, which requires further study.^54^ Lignans are formed by the oxidative polymerization of phenylpropane, and most of them are dimers, while a few are trimers or tetramers. Several kinds of lignans have been found to have antiaging effects, including Sesamin, Sesamolin, and Sesamol;^55−58^ Arctigenin, Matairesinol, Arctiin, Lsolappaol A, Lappaol C, and Lappaol F;^59^ and nordihydroguaiaretic acid.^60^ For example, multiple pathways are involved in the longevity effect of Sesamin, which is abundant in the healthful food sesame. These include the *sir-2.1*/SIRT1, *aak-2*/AMPK, mechanistic target of rapamycin (mTOR), and IIS pathways. All these pathways have been shown to mediate caloric restriction (CR) signaling. However, the direct molecular targets of Sesamin are still not well understood. which riched in healthy food sesame.^58,56^ In addition, Arctigenin, Matairesinol, Arctiin, Lsolappaol A, Lappaol C, and Lappaol F isolated from the seeds of *Arctium lappa*, a famous herbal medicine utilized to treat arthritis, baldness, or cancer, also prolong worms’ mean lifespan under normal and oxidative stress conditions via the upregulation of *jnk-1* gene expression. Subsequently, DAF-16 nuclear localization is promoted through a JNK-1-DAF-16 cascade.^61^ However, their antiaging effects need to be further evaluated in other model organisms.

Importantly, interest in phenylpropanoids has increased in recent years due to their bioactive properties and abundant daily intake, so further research on their antiaging properties in mammals would help to pave the way for clinical trials.

#### 2.4.2 Flavonoids

Flavonoids are 2- or 3-phenyl derivatives of chromane or chromone that generally refer to a series of compounds formed by the connection of two aromatic rings (A and B) through a central three-carbon chain, which generally have the basic skeleton characteristics of C6 (ring A)-C3 (ring C)-C6 (ring B). Flavonoids mainly exist in the form of glycosides in flowers, leaves and dietary fruits and in a free state in the xylem of plants. As phenolic compounds are widely distributed in higher plants, most of them have color and are one of the main active components in medicinal plants.^62^ Due to their diverse bioactivities and low toxicity, they have attracted extensive attention worldwide and have become a hot spot in research, development and utilization.

Flavonoids are generally classified according to the degree of oxidation, annularity of ring C, and connection position of ring B. Their typical structures can interact with enzyme systems involved in critical pathways,^63,64^ which has led to the abundance of studies on the relationships between their chemical structures and activities, such as antiviral/bacterial, anticancer, and antioxidant activities.^65^ The most commonly studied flavonoids include flavones, flavanones, flavonols, flavanonols, isoflavones, isoflavanone, chalcones, aurones, and homoisoflavones, among which a total of 37 kinds of flavonoids exhibit antiaging effects (Supplementary Table 4).

The flavonol Fisetin and the chalcone 4,4′-dimethoxychalcone (DMC) significantly promote the healthspan and even the maximum lifespan of mammals and thus have received extensive attention. Fisetin can reduce the percentage of senescent cells *in vivo*, improve healthspan and extend the mean and maximum lifespan of wild-type mice, acting as a senotherapeutic compound in late life. Clinical trials on its antiaging effects are currently underway.^66^ DMC, a chalcone found in *Angelica keiskei*, can extend the maximum lifespan of yeast, worms, and flies in an autophagy-dependent manner and can exert additional cytoprotective effects from yeast to mice by inhibiting specific GATA transcription factors.^67^ Further studies could explore the beneficial health effects of this promising chalcone on humans.

As far as our current knowledge is concerned, the observed antiaging effects in the same group of flavonoids are hard to generalize to a common mechanism; thus, for future research, an interesting point is whether the chemical structures of flavonoids correlate with their antiaging activities. Aging is a complex process involving multiple factors.^68^ In contrast, current explorations into the antiaging mechanisms of phytochemicals are still relatively superficial, with the targets of most flavonoid compounds remaining unknown. Since most of the current studies have been conducted in worms and the research content is limited to stress resistance and nutrient perception, more phenotypes need to be validated in various model organisms to verify the antiaging effects of these flavonoids.

#### 2.4.3 Phenolic Glycoside

Glycosides are compounds formed by connecting saccharides or saccharide derivatives with another substance called aglycone through the terminal carbon atom of saccharides. The commonality of glycosides is the saccharide moiety, while the aglycone moiety covers almost all types of natural products, such as steroid glycosides, terpenoids glycosides and phenolic glycosides. Phenolic glycosides (such as flavonoid glycosides and stilbene glycosides) may differ from their aglycone forms, as their solubility in water generally improves their bioavailability from the diet, and glycosylation usually increases aqueous solubility.^69^ Then thehydrolysis of glycosides can release free aglycone *in vivo*(70) and potentiate bioactivity and exhibit more bioavailability. Icariin, the major bioactive compound in *Epimedium brevicornu* Maxim, has multiple pharmacological effects^71^ and promising antiaging properties, including its healthspan extension and/or lifespan extension effects in *C. elegans* and C57BL/6 mice.^72^ In *C. elegans*, Icariside II, the bioactive form of Icariin *in vivo*, can increase resistance to thermos-stress and oxidative stress and ameliorate polyQ aggregation.^73^ In C57BL/6 mice, Icariin can upregulate the expression of mammalian protein kinase ataxia telangiectasia mutated (ATM), which may help maintain genome stability and enhance the activities of antioxidant enzymes, thereby synergistically contributing to healthspan extension.^72^ Another glycoside, Tetrahydroxystilbene glucoside (TSG) was isolated from the root of *Fallopia multiflora*, an esteemed TCM historically revered for its potential to promote longevity. Further mechanistic studies revealed that TSG extends the maximum lifespan of senescence-accelerated mouse prone 8 (SAMP8) by 17% via the IIS pathway in the brain.^74^

#### 2.4.4 Other Polyphenols

The isomers of both Catechin and Epicatechin are the most common monomers of condensed tannins, belonging to flavan-3-ols, which are abundant in the leaves of *Camellia species* and *Acacia catechu*. Catechin extends the maximum lifespan of worms by activating mitophagy,^75^ while Epicatechin increases the lifespan of *C. elegans*(76) and *D. melanogaster*,^77^ as well as the survival rate of C57BL/6 mice with supplementation for 37 weeks.^78^ A recent study also highlighted the antiaging effects of the trimeric Epicatechin Procyanidin C1 (PCC1) isolated from grape seeds, which increases the healthspan and maximum lifespan of naturally aged mice. Its functional mechanism is thought to involve processes that inhibit senescence-associated secretory phenotype (SASP) formation at low concentrations and selectively kill senescent cells by promoting the production of ROS and mitochondrial dysfunction at high concentrations.^79^ As a natural senotherapeutic agent, PCC1 has safety advantages and is expected to become a novel and effective candidate for delaying aging.

Research on plant polyphenols is accelerated because of their abundant and safe daily intake, and, more importantly, the synergistic effect of polyphenol combinations or polyphenol-rich food combinations should be given more attention, which will lead to better utilization of polyphenols to delay aging.

### 2.5 Terpenoids

Among plant secondary metabolites, terpenoids (isoprenoids) represent the most abundant and diverse group of natural phytochemicals, and each plant can synthesize hundreds of terpenoid compounds.^80,81^ Terpenoids are derived from the mevalonate pathway and are formed through the condensation and subsequent modification of isoprene units in various ways.^82^ They are usually divided into monoterpenoids, sesquiterpenoids, diterpenoids, triterpenoids, tetraterpenoids, and more, according to the number of isoprene units. Many monoterpenoids and sesquiterpenoids are part of essential oils, while triterpenoids are often combined with glycosides to form saponins.

Terpenoids, owing to their complex structures and diverse effects, are ideal molecules in the field of natural product activity research and can be used to discover and search for novel bioactive leads for drugs. Some of them are prominent, such as the sesquiterpenoid Artemisinin for antimalaria,^83^ the diterpenoid Paclitaxel for anticancer therapy,^84^ and the triterpenoid saponin Ginsenoside with many biological properties.^85^ In Supplementary Table 5, we list the antiaging effects of 32 terpenoids by classification with 1 special hemiterpene, 6 monoterpenoids, 6 sesquiterpenoids, 8 diterpenoids, and 11 triterpenoids, hoping to find some rules on the molecular mechanism. However, almost all terpenoids have only been tested in yeast or worms, with the exception of Ginsenoside Rg1 and Ursolic acid, which have exhibited promising antiaging properties in mammals.^86−88^ Ursolic acid, an ursane-type pentacyclic triterpenoid found in *Arctostaphylos uva-ursi*, *Mentha canadensis*, *Lavandula angustifolia*, and *Thymus mongolicus*, can increase the healthspan and maximum lifespan of *D. melanogaster* and enhance the levels of SIRT1 and SIRT6 in C5BL/6 mice.^89,90^ Moreover, it can upregulate the *srl* gene, the ortholog of mammalian peroxisome proliferator-activated receptor-gamma coactivator-1α (PGC-1α) in flies^90^ and PGC-1β in mice,^89^ both of which are closely related to mitochondrial biogenesis.

In terms of the types of carbocyclic skeletons and their abundance, terpenoids are the most abundant class of plant secondary metabolites. Moreover, the number of sugar molecules and their linkage in terpenoid glycosides imbue them with unique chemical structures and associated bioactivities. Studies have indicated that an increase in antitumor effects correlates with a decrease in the number of sugar moieties in a ginsenoside.^91^ Specifically, the C-3 sugar moiety contributes to extended circulation *in vivo* and augments the tumor active targeting ability of ginsenosides.^92^ Considering the success in the discovery and utilization of Artemisinin, we firmly believe that terpenoids can be introduced to humans.

### 2.6 Steroids

Composed of three cyclohexanes (rings A, B, and C) and one cyclopentane (ring D), steroids represent a specific class of terpenoid lipids that share a 17-carbon-atom skeleton and are widely present in animals, plants and microorganisms.^93^ Steroid glycosides, also referred to as steroidal saponins, are primarily forms of steroid found in plants such as Dioscoreaceae, Liliaceae, and Scrophulariaceae. The primary metaskeletons of steroidal saponins include spirostane, furostane, cholestane, and cardenolide.^94^ In this review, five out of seven are classified as steroidal saponins (Supplementary Table 6). Steroids are lipophilic and can easily enter cells and then interact with nuclear receptors and/or membrane proteins to mediate many physiological functions, such as the regulation of signal transduction pathways, cell proliferation and differentiation.^95−97^ For a long time, steroid-based therapeutic drugs have drawn attention from researchers and pharmaceutical companies due to their low toxicity and high bioavailability.^98−100^ Approximately 300 steroid-based drugs have been marketed,^101^ with many therapeutic properties.^102^

Regarding their antiaging effects, seven kinds of steroids distributed in plants, namely, *Asparagus racemosus*, *Acnistus arborescens*, *Convallaria majalis*, *Cynanchum otophyllum*, *Dioscorea* spp., *Gentiana rigescens*, and *Ophiopogon japonicus*, have been shown to prolong the lifespan of yeast, worms, and flies, suggesting their potential to delay aging and/or aging-related diseases. For instance, a representative steroid Withaferin A, found in the leaves of *Acnistus arborescens*, prolongs the median as well as the maximum lifespan of flies by regulating genes associated with multiple aspects, such as antioxidant defense, recognition of DNA damage, and repair of double-strand breaks.^103^ In addition, previous studies have suggested that some widely researched sex steroids, such as 17β-estradiol and testosterone, can activate molecular components of aging-associated pathways in mammals,^104^ and thus, the longevity extension effects of certain plant steroids warrant further verification in mammalian models.

### 2.7 Alkaloids

Alkaloids are widely distributed in nature and are one of the most important classes of organic nitrogen compounds in plant secondary metabolites. They possess physiological activities and usually serve as a rich source for drug discovery because of their diverse structures and biological effects.^105,106^

Some well-known alkaloids, such as Morphine from *Papaver somniferum* for pain relief^107^ and Vincristine from *Catharanthus roseus* with anticancer effects,^108^ have already been successfully developed into drugs. In terms of antiaging, eight alkaloids have been shown to have the capability to extend the lifespan of worms, two alkaloids have been shown to promote the longevity of flies, and two alkaloids have been found to delay mammalian aging, showing their potential benefits for longevity (Supplementary Table 7). For example, Tetramethylpyrazine and Berberine are considered to have antiaging activity in mammals. Tetramethylpyrazine is one of the main bioactive components extracted from the TCM *Ligusticum sinense*, which can suppress NF-κB signaling and reduce the levels of proinflammatory factors, thereby significantly improving cell viability and delaying bone marrow mesenchymal stem cell senescence.^109^ Berberine (BBR) found in *Coptis chinensis* can not only prolong the maximum lifespan of doxorubicin-induced aging mice but can also affect naturally aged mice, as experimental results revealed that BBR can ameliorate DNA damage by downregulating the expression of p16 and thereby triggering a series of downstream reactions.^110^ Further pharmacological evaluations of these antiaging compounds should be undertaken to identify whether any of these alkaloids could delay aging and/or treat age-related diseases as new lead compounds.

### 2.8 Others

For the time being, some phytochemicals cannot be classified in the manner we currently use, so they are listed as others (Supplementary Table 8). Quinic acid, a naturally occurring organic compound found abundantly in dietary foods like apples and peaches,^111^ exhibits an antiaging effect in *C. elegans*, as evidenced by the lifespan extension of worms under normal conditions, heat stress, and oxidative stress.^112^ Additionally, five organosulfur compounds have been reported to extend the lifespan of worms. These include Allicin,^113^ an unstable compound released from garlic, and its decomposition product, Diallyl trisulfide.^114^ S-allylcystein and S-allylmercaptocysteine are formed in significant amounts during garlic’s aging process through enzymatic hydrolysis.^115^ Lastly, the broccoli-derived isothiocyanate Sulforaphane has also been shown to increase worm lifespan.^116^ Interestingly, all four garlic-derived organosulfur compounds can exhibit antiaging properties in either worms or mice, at least partially by activating SKN-1/Nrf, owing to their common thioallyl structure and disulfide bonds.^113−115^ This makes it possible for natural thioallyl compounds to be utilized in nutraceutical products and drugs that target the Nrf pathway.^115^

## 3 Mechanisms of Phytochemicals for Longevity Promotion

Considering the molecular target, related signaling pathway and the actual effects, we divided almost all of the phytochemicals into six categories by their possible mechanisms of promoting longevity, which are closely linked to the fundamental function of cells and organisms. They are (1) regulation of the ROS levels, (2) regulation of the nutrient-sensing network: phytochemicals affect nutrient-sensing signaling pathways such as IIS, AMPK signaling, and mTOR signaling; (3) regulation of mitochondria: phytochemicals induce mitophagy and/or mitohormesis (mitochondrial hormesis) or enhance antioxidant enzymes and maintain ROS levels in an appropriate range; (4) regulation of the autophagy; (5) regulation of the gut microbiota: phytochemicals participate in the alteration of the composition of the gut microbial community and microbial metabolism; and (6) regulation of lipid metabolism: phytochemicals affect lipid metabolism or may enhance the activity of the transcription factor HSF-1. We will discuss how phytochemicals regulate longevity in these six aspects.

**Figure 1:**
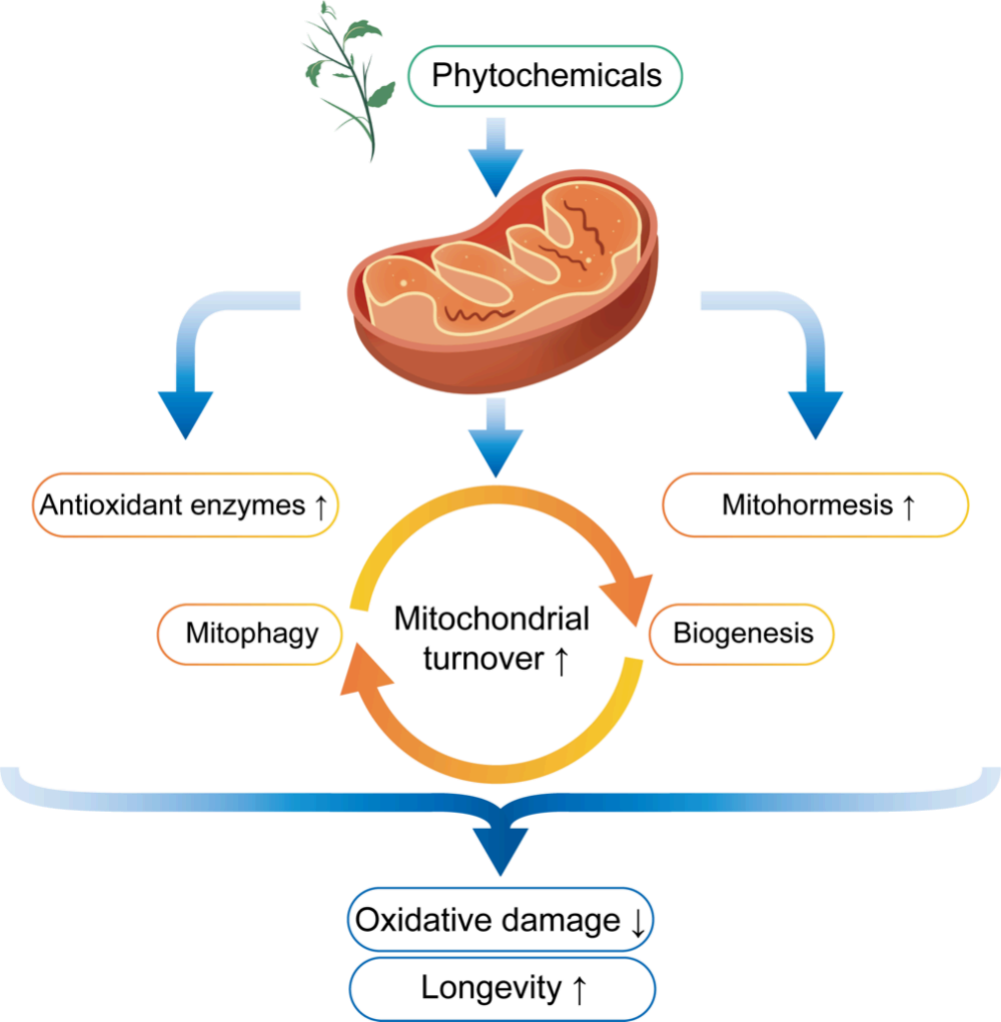
Phytochemicals regulate antioxidant enzymes and mitochondrial functions. Phytochemicals enhance the activities of antioxidant enzymes, induce mitohormesis effects, and maintain the balance of mitophagy and biogenesis, ultimately reducing oxidative damage and promoting the longevity of organisms.

### 3.1 Phytochemicals Regulate Longevity through ROS Levels

ROS is an umbrella term for an array of derivatives of molecular oxygen that occur as a normal attribute of aerobic life. Elevated formation of the different ROS leads to molecular damage. Two species, hydrogen peroxide (H_2_O_2_) and the superoxide anion radical (O^2–^), are key redox signaling agents generated under the control of growth factors and cytokines by more than 40 enzymes, prominently including NADPH oxidases and the mitochondrial electron transport chain.^117^ The damaging roles of oxidants are consistent with the hallmarks of aging, which include mitochondrial dysfunction, protein denaturation and aggregate formation, altered cell membranes and intercellular communication, loss of regenerative cell populations owing to cell death and senescence, and genomic instability.^118,68^ The intracellular concentration of H_2_O_2_ is maintained in the low nanomolar range (approximately 1–100 nM), being under tight control: the generation of H_2_O_2_ is stimulated by metabolic cues or by various stressors, such as growth factors, chemokines or physical stressors,^119^ while its removal is achieved by efficient reducing systems. Steady-state physiological flux of H_2_O_2_ to specific protein targets leads to reversible oxidation, thereby altering protein activity, localization and interactions, which contributes to orchestration of various processes in cells and organs, including cell proliferation, differentiation, migration and angiogenesis.^120,121^ Supraphysiological concentration of H_2_O_2_ (roughly estimated to be above 100 nM) leads to unspecific oxidation of proteins and altered response patterns as well as to reversible and irreversible damage to biomolecules, causing growth arrest and cell death, with associated pathological states.^122^ This review includes approximately 55 phytochemicals that can regulate ROS levels, covering almost all types of phytochemicals (Supplementary Table 1–8). The antioxidant function of polyphenols and flavonoids is the most commonly and deeply studied and is achieved through multiple mechanisms, such as chelation of metals (iron and copper ions), enhancement of the activity and expression of antioxidant enzymes, and inhibition of ROS-producing enzymes.^123^ For example, Curcumin can reduce ferric ions (Fe^3+^) and chelate ferrous ions (Fe^2+^) to prevent hydroxyl radical generation from hydrogen peroxide through the Fenton reaction.^124^ Apigenin, Catechin, Kaempferol, Luteolin, and Quercetin are also found to inhibit ROS-producing enzymes such as xanthine oxidase, monoamine oxidase, and NADPH oxidase.^125^ Notably, 4 garlic-derived organosulfur compounds that have a common thioallyl structure and disulfide bond could selectively induce SKN-1 targets involved in oxidative stress defense in worms, which emphasizes the importance of phytochemical structure.^113−115^

### 3.2 Phytochemicals Regulate Longevity through a Nutrient-Sensing Network

Nutrient-sensing signaling is an integrated network of multiple pathways, including the IIS (insulin/IGF-1) pathway, AMPK (adenosine monophosphate (AMP)-activated protein kinase), mTOR, Sirtuins, and Forkhead Box O (FOXO). They are interlinked and comodulate the growth, metabolism, and longevity of organisms. How phytochemicals connect to this network has been elegantly summarized in several recent reviews;^10,126,127^ thus, here, we only give a brief overview of their roles in longevity extension.

A main nutrient-sensing pathway involves insulin/IGF-1 signaling, which regulates anabolism and energy storage and promotes cell growth and protein synthesis, respectively.^128^ It is well-known that the reduction in IIS increases lifespan in a variety of model organisms, and its downstream targets FOXO transcription factors and mTOR are considered to play crucial roles. The FOXO family was demonstrated to modulate hundreds of downstream targets, including many genes involved in the oxidative stress response and metabolism.^129^ Various phytochemicals, such as quinonoids, terpenoids, and alkaloids, have been reported to modulate the insulin/IGF-1/FOXO pathway to exhibit antiaging effects in worms, flies, and rodents (Supplementary Table 3, Supplementary Table 5, and Supplementary Table 7), reflecting its conservatism in evolution.

AMPK and mTOR are opposing signaling pathways involved in sensing nutrients and energy while modulating cell growth and overall metabolism. mTOR can be activated by growth factor stimulation or by increased intracellular amino acid levels, thereby promoting cell growth.^130^ Although AMPK, as its name suggests, is thought to be activated by AMP, it is now clear that the ancestral role of AMPK is to sense low glucose. Glucose has been argued to be a messenger. Glucose starvation induced by CR or phytochemicals can activate AMPK and inhibit mTOR, thereby suppressing anabolism and meeting glucose demand by promoting lipid oxidation, utilizing nonessential amino acids for gluconeogenesis, or regulating autophagy through phosphorylation of ULK1.^131,132^ During this process, limited energy may be shifted away from growth and reproduction to maintenance and repair. Two well-known compounds, that is, rapamycin and metformin, that exert longevity-extending effects can inhibit mTOR and activate AMPK, respectively.^133^ Recent studies have also screened for some novel phytochemicals with similar effects, such as a sesquiterpenoid named Thapsigargin and another diterpenoid named Rebaudioside A.^134−136^

Sirtuins are NAD^+^-dependent histone deacetylases that modulate cellular functions by removing acyl groups on histones and other proteins.^137^ Considering that NAD^+^ and NADH take part in nutrient oxidation, sirtuins can sense the energy state of the cell. Moreover, the activation of AMPK elevates the levels of NAD^+^, suggesting that AMPK can also induce the activation of sirtuins, although how NAD^+^ is increased by AMPK remains unknown.^138^ Sirtuins deacetylate and activate PGC-1α, thereby stimulating mitochondrial biogenesis while also deacetylating FOXO proteins.^139^ Resveratrol, for instance, can induce the activation of sirtuins and promote longevity from yeast to flies while exerting beneficial effects in healthy aging, but its target(s) is still unclear.^139,140^

In brief, some phytochemicals act as mild biological stressors that modulate nutrient-sensing networks and starve cells and induce subsequent cellular catabolic processes such as autophagy, which utilizes excess products of cellular metabolism to provide nutrients back to the organism and to reduce damaged molecules and organelles while stimulating stress responses.^43^ Therefore, phytochemicals may shift nutrients from reproductive and biosynthetic processes to processes involved in maintenance and repair, eventually reducing the pace of aging and promoting longevity.^141^

### 3.3 Phytochemicals Regulate Longevity through Mitochondria

Mitochondria are crucial organelles, as they play key roles in cellular metabolism and are also regarded as potential central regulators of aging.^142^ Mitochondria regulate many physiological functions, such as adenosine triphosphate (ATP) synthesis, free radical generation, fatty acid β-oxidation, and cell survival and death.^143,144^ The production of ATP through oxidative phosphorylation is the most important physiological function of mitochondria. During this process, mitochondria inevitably produce superoxide anions as a byproduct, which can be further converted into ROS.^145^

Mitochondria are the main source of intracellular ROS. Mitochondrial ROS, especially hydroxyl radicals, can react with and damage macromolecules such as proteins, nucleic acids, and phospholipids, thereby impairing their structures and functions.^146^ Damaged mitochondria induce the mitochondrial unfolded protein response (UPR^*mt*^), which functions to maintain proteostasis and slow down the pace of oxidative damage to mitochondria.^147^ UPR^*mt*^ can enhance the activities of certain antioxidant enzymes, such as CAT, SOD, and GSH-PX,^148^ and trigger a broad transcriptional antioxidant response.^149,150^ However, the ROS scavenging system cannot completely prevent excessive ROS-mediated mitochondrial damage, while damaged and dysfunctional mitochondria will be scavenged through mitophagy, a process for mitochondrial degradation.^151^ Moreover, damaged mitochondria are replaced with newly synthesized mitochondria to achieve a balance between mitophagy and mitochondrial biogenesis to maintain normal mitochondrial functions.^141,152^ Interestingly, with age, UPR^*mt*^ tends to gradually lose adaptive signaling potency and is no longer able to address various stresses from age-related cellular damage.^153^ Furthermore, there is also evidence that the levels of mitophagy and mitochondrial biogenesis significantly decline in mammalian tissues during aging,^154^ which are thought to cocontribute to various age-related pathologies. The accumulation of damaged mitochondria and mitochondrial ROS can reinforce each other, forming a vicious cycle that eventually leads to mitochondrial dysfunction and promotes the aging process.^143^

More interestingly, a mild mitochondrial stress named mitohormesis is an attractive concept, which means that coordinated responses to slight mitochondrial stress appear to make cells less vulnerable to later perturbations,^155^ which can contribute to healthy longevity due to conserving cellular and organismal homeostasis during stressful conditions and the aging process.^156,157^ Several phytochemicals, such as quinonoids Juglone and alkaloids Harmol, have been reported to induce mitohormesis.^35,158^*In vitro* treatment with Harmol causes a transient mitochondrial depolarization, which triggers mitohormesis responses. This, in turn, extends the lifespan of worms and flies, as well as improves the healthspan of naturally aged mice. Mechanistically, the combined modulation of harmol’s targets—monoamine-oxidase B (MAO-B) and the GABA-A receptor (GABAAR), reproduces the mitochondrial improvements induced by harmol. This suggests that these two targets play a key role in mitohormesis.^158^ As it might be difficult to clearly define the boundary between mitohormesis and bioenergetic collapse, the dosage of phytochemicals requires careful consideration.

Some studies have shown that the longevity-extending effects of several phytochemicals are accompanied by increased mitochondrial biogenesis, which is largely coordinated by the transcriptional coactivator PGC-1α.^159,160,150^ Both the phenylpropanoid Chicoric acid, a caffeoyl derivative, and the polyphenol Epigallocatechin-3-gallate have been identified to promote longevity by upregulating the AMPK pathway in worms. AMPK upregulation further elevates the NAD^+^-to-NADH ratio and activates SIRT1, thereby promoting the accumulation of PGC-1α in the nucleus and the transcription of genes that are essential for mitochondrial biogenesis.^160,51^ Therefore, many phytochemicals that depend on AMPK and SIRT1 for their antiaging effects may act at least in part by activating PGC-1α,^161^ which suggests that PGC-1α may be a promising target for longevity.

Clearly, as the supreme metabolic entities, mitochondria are significantly multifunctional and intimately involved in numerous cellular processes, including aging.^145^ Many phytochemicals restore mitochondrial function by reducing mitochondrial oxidative damage and/or regulating the balance of mitophagy and mitochondrial biogenesis, namely, mitochondrial turnover, thereby maintaining the healthy metabolic state of organisms and promoting their longevity (Figure 1).

**Figure 2:**
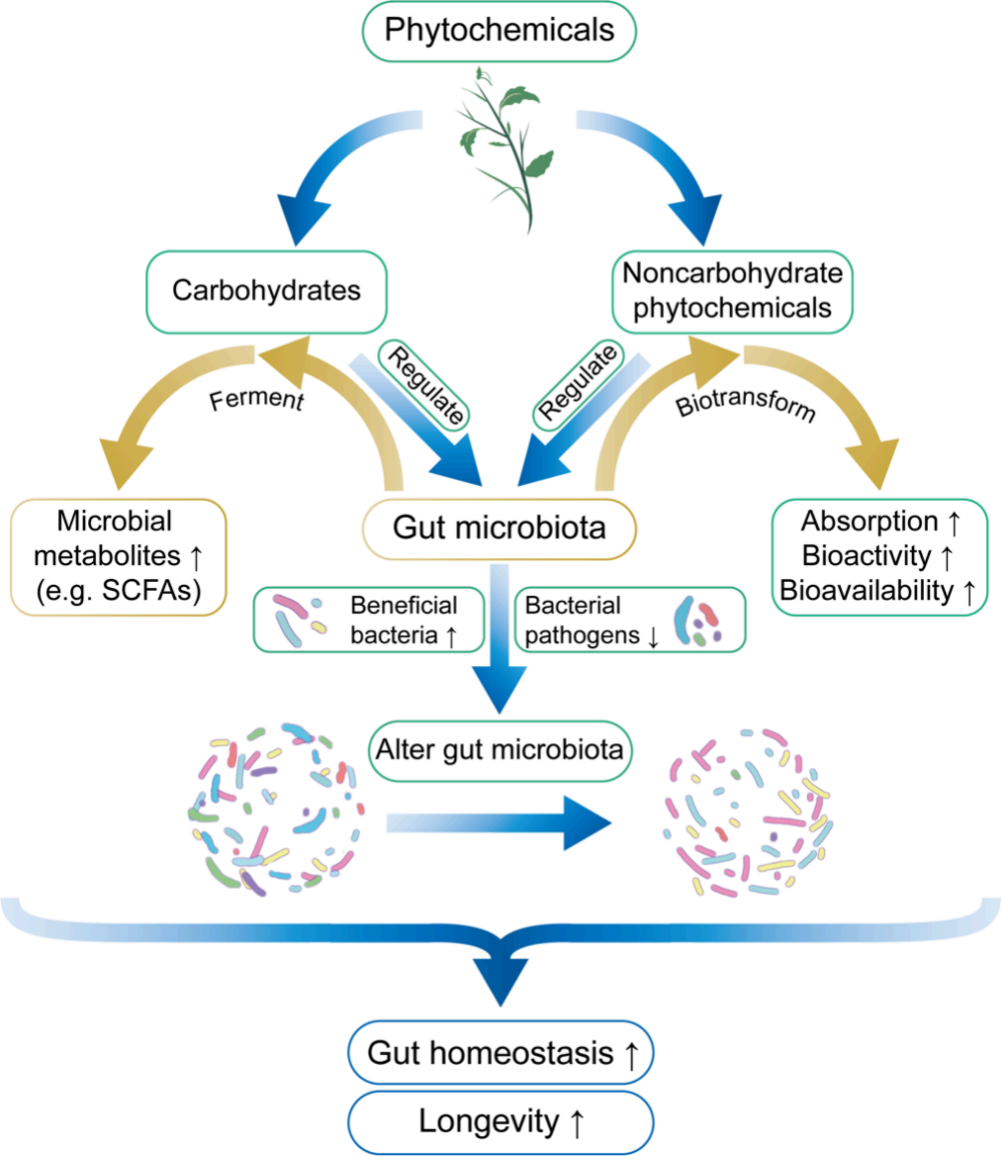
Phytochemicals regulate longevity through the gut microbiota. Phytochemicals can regulate the gut microbiota and alter its composition, stimulate the growth of beneficial bacteria and inhibit the reproduction of intestinal bacterial pathogens. Moreover, the gut microbiota ferments carbohydrates such as polysaccharides and dietary fiber to yield beneficial microbial metabolites, and noncarbohydrate small molecule phytochemicals can be biotransformed to metabolites with better absorption and bioactivity. Together, these effects maintain gut homeostasis and promote longevity.

### 3.4 Phytochemicals Regulate Longevity through the Autophagy

Autophagy is a process that involves the sequestration of cytoplasmic material into two-membrane vesicles, known as autophagosomes. These subsequently fuse with lysosomes to digest their luminal content. This process not only plays a role in maintaining proteostasis but also impacts nonproteinaceous macromolecules such as ectopic cytosolic DNA, lipid vesicles, and glycogen. It also affects entire organelles, including dysfunctional mitochondria targeted by ’mitophagy’, and other organelles leading to ’lysophagy’, ’reticulophagy’, or ’pexophagy’. Additionally, it plays a role in the digestion of invading pathogens, a process known as ’xenophagy’.^162^ In humans, the expression of autophagy-related genes such as *ATG5*, *ATG7*, and *BECN1* decreases with age.^163^ CD4+ T lymphocytes isolated from the offspring of parents with exceptional longevity exhibit enhanced autophagic activity compared to age-matched controls.^164^ A decline in autophagy in circulating B and T lymphocytes from aging donors is accompanied by a reduction of the pro-autophagic metabolite spermidine.^165,166^ The age-related decline in autophagy is one of the most significant mechanisms contributing to reduced organelle turnover, justifying its discussion as a hallmark of aging.^68^

Autophagy induction via small molecules, such as rapamycin, has typically been achieved through the inhibition of mTORC1 or the activation of AMPK. The inhibition of mTORC1 prevents its phosphorylation of ATG13, ULK1 and ULK2 within the ULK1 complex. This allows for the phosphorylation and activation of ULK1 by AMPK, leading to an increase in autophagy levels.^167^ Rapamycin and its related rapalogs induce autophagy through formation of a complex with FK506-binding protein (FKBP12), which acts as an allosteric inhibitor of mTORC1, thereby blocking its kinase activity.^168,169^ The emerging antiaging isoflavone glycoside Puerarin, can enhance lysosome-involved autophagy by promoting the expression of β-galactosidase and lysosomal associated membrane protein 1 (LAMP1), and increasing the levels of autophagy-related genes, which results in the extension of the lifespan of *D. melanogaster*.^170^

Mitophagy is another mechanism for mitochondrial protection that can be induced by some phytochemicals,^151^ such as an alkaloid Tomatidine and a polyphenol Catechin. However, the mechanisms of their lifespan-extending effects are quite different. PTEN-induced kinase 1 (PINK1) on the mitochondrial outer membrane plays a key role in recruiting autophagy receptors, combining damaged and/or dysfunctional mitochondria and autophagosomes, and subsequent mitochondrial degradation.^171^ Tomatidine moderately increases ROS levels and induces mitohormesis and then activates the SKN-1/Nrf2 pathway, which in turn upregulates the PINK1/DCT-1 (an ortholog of human BCL2 interacting protein 3, BNIP3) pathway and stimulates mitophagy,^172^ while Catechin may induce mitophagy through direct regulation of the genes *beclin 1* (*bec-1*) and *pink-1*,^75^ as it does not exhibit mitohormetic effects despite its high dose.

### 3.5 Phytochemicals Regulate Longevity through the Gut Microbiota

From worms to humans, the gut microbiota carries out a series of metabolic activities that affect the local intestinal environment as well as host metabolism.^173^ The healthy gut microbiota plays a crucial role in the control of metabolism, resistance to infection and inflammation, and the regulation of the brain-gut axis.^174^

Here, we highlight the antiaging effect of gut microbiota-involved phytochemical metabolism. The gut microbiota contains more metabolic enzymes than the host genome and thus has stronger metabolic ability. Many dietary phytochemicals, such as some polysaccharides, Ginsensoide Rb1, and Ellagitannins (or Ellagic acid), which are indigestible by the human body and/or possess limited intestinal absorption, can be metabolized by the gut microbiota. During this process, different kinds of phytochemicals can alter the composition of the gut microbial community and regulate microbial metabolites to exert multiple physiological functions.^175^ Moreover, phytochemicals can stimulate the growth of beneficial bacteria while inhibiting the reproduction of intestinal bacterial pathogens,^176^ thereby maintaining gut homeostasis and indirectly regulating longevity (Figure 2).

**Figure 3:**
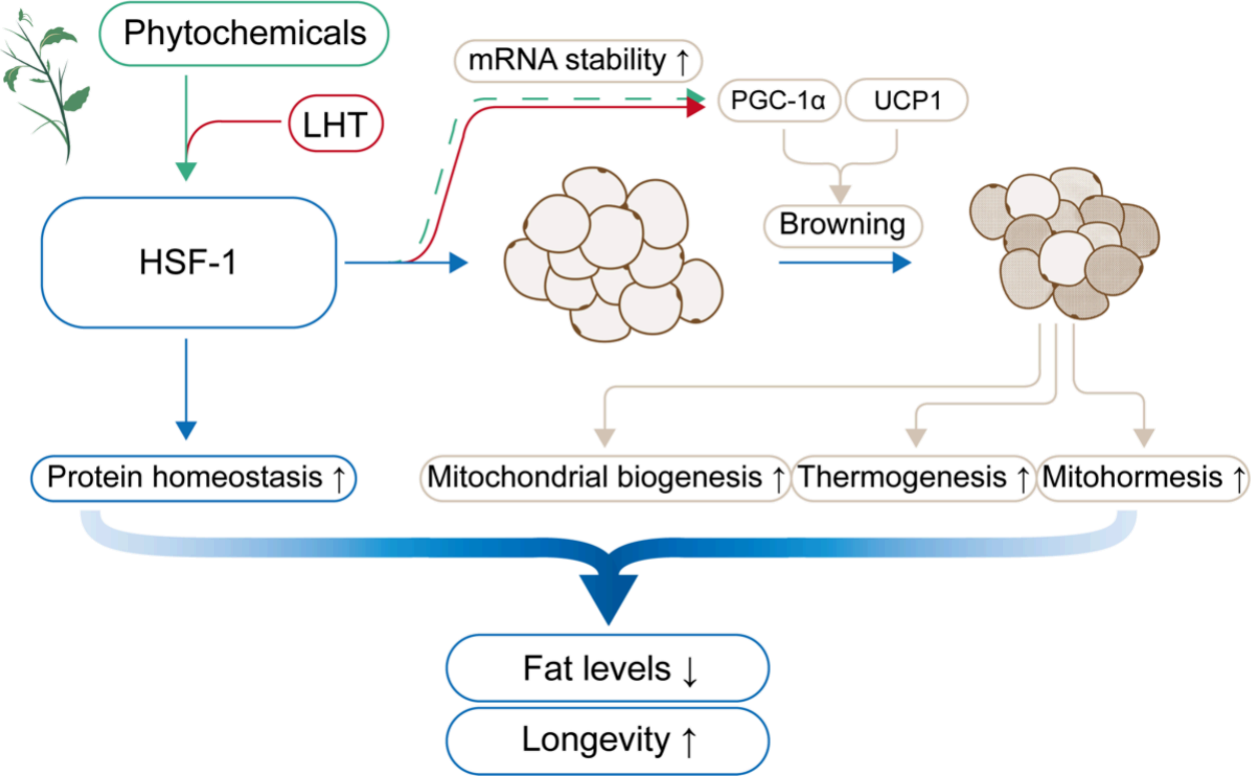
Phytochemicals regulate lipid metabolism partially through HSF-1-mediated reduction of fat levels. The activation of HSF-1 induced by LHT increases the mRNA stability of key browning genes, and mitochondrial biogenesis, thermogenesis, and mitohormesis are triggered by these genes, ultimately reducing fat levels. Some phytochemicals can also activate HSF-1 to maintain protein homeostasis and may decrease fat levels through the same mechanism, which can ultimately promote longevity.

Phytochemicals, especially carbohydrates, such as various polysaccharides and oligosaccharides, have beneficial effects on the host via the regulation of the gut microbiota composition without being directly assimilated by the body.^177^ For example, after successive degradation, gut microbial fermentation of indigestible plant polysaccharides yields short-chain fatty acids (SCFAs). Once SCFAs are absorbed by colonocytes, they can be transported into the metabolic cycle to reach other tissues as energy substrates, contributing to intestinal homeostasis, systemic metabolism, and brain function.^178^ Children who consume a large number of plant polysaccharides in rural Africa have quite different gut microbiota compositions from those of Italian children, especially the levels of *Prevotella* and *Xylanibacter*. These microbiota can degrade cellulose and xylan and are associated with elevated levels of fecal SCFAs, suggesting that more plant polysaccharides and less sugar and fat can increase the proportion of gut microbiota that produce SCFAs to protect African children from inflammation and noninfectious colonic diseases.^175,179^ Interestingly, the levels of microbial SCFAs decrease with aging.^180^ Moreover, SCFAs supplementation can protect the host from age-related pathologies, and direct intake of SCFAs can prolong the lifespan of worms and flies by inhibiting histone deacetylation.^181^ A recent investigation revealed that the dietary intake of Genistein, which has been found in almost all leguminous plants including soybeans and coffee beans, increases *Lachnospira* abundance and SCFAs production within the mice gut. Additionally, this dietary intake enhances the health and longevity of the mice while also modulates homeostasis of aging gut.^182^ Concurrently, healthier plant-based dietary patterns, characterized by higher consumption of whole grains, fruits, vegetables, nuts, legumes, and tea—which are thought to be rich in plant polysaccharides and dietary fiber—are correlated with reduced mortality rates among older adults, implying an extended longevity.^5^ The additional longevity may be partially attributable to the physiological function of SCFAs. While further evidence is necessary, this could elucidate why certain seemingly indigestible plant polysaccharides contribute to the increased longevity of the host organisms.

The gut microbiota can also biotransform noncarbohydrate phytochemicals into microbial metabolites with different bioavailability and bioactivity/toxicity from their precursors, which contributes to better absorption and utilization by the organism^176^ (Figure 2). For example, because of the increased number of hydrogen bonds and increased polar surface area, glycosides (e.g., triterpene glycosides and flavonoid glycosides) are limited in intestinal absorption.^183^ Since abundant bacterial phyla (dominated by Bacteroidetes and Firmicutes) encode abundant glycoside hydrolase genes, these gut microbiota are specialized for glycosidase hydrolysis.^184^ Through deglycosylation catalyzed by the gut microbiota, glycosyl or glucuronosyl moieties can be gradually cleaved from the backbone, and the resulting secondary glycoside(s) and/or glycogen usually have better intestinal absorption and therefore better bioavailability. For example, Ginsensoide Rb1, the major dammarane-type tetracyclic saponin in ginseng, can protect rat neural progenitor cells against oxidative injury by enhancing the Nrf2/HO-1 pathway and thereby alleviating oxidative stress in aged mice when administered orally.^185,186^ Ginsensoide Rb1 can be metabolized by stepwise deglycosylation to generate compounds such as 20(S)-protopanaxadiol, which is considered to be the effective ingredient of orally administered Ginsensoide Rb1, rather than the compound itself. The gut microbiota also biotransforms Ellagitannins into Ellagic acid and finally into Urolithins (including Urolithins A, B, C, and D), which possess good systemic exposure and can be better absorbed.^187^ A recent study showed that Ellagic acid can prolong the lifespan of *D. melanogaster*.^90^ Likewise, Urolithin A has been proven to enhance mitochondrial and muscle functions by inducing mitophagy in rodents and extending the longevity of *C. elegans* models.^188^ Therefore, the benefits of Ellagitannins (or Ellagic acid) on health and longevity are more likely to be due to the function of Urolithins produced by gut microbiota metabolism. The limitations of previous studies also exist, to confirm the role of gut microbiota or phytochemicals on lifespan, the effects of phytochemicals should be performed using axenic animals. While recent studies have shown that rapamycin treatment improves gut health and modulates microbiota composition during aging,^189,190^ its longevity-extending effects are proven independently of the gut microbiota in axenic *Drosophila*.^190^ Therefore, it is necessary for germ-free models as blank control to explore the regulation of longevity mediated by gut microbiota and/or phytochemicals.

### 3.6 Phytochemicals Regulate Longevity through Lipid Metabolism

It seems to be a coincidence that controlling dietary calories without malnutrition and regular exercise help to reduce fat and promote the healthspan and/or lifespan in various organisms, including humans, mice, and monkeys.^2^ Furthermore, the phytochemical Epigallocatechin gallate (EGCG), a polyphenol from tea, significantly reduced the total cholesterol and low-density lipoprotein plasma levels of 115 women with central obesity at a high daily dose, without any side effects or adverse events.^191^ Similarly, Resveratrol can also reduce fat levels in rodents.^192^ Both EGCG and Resveratrol promote the longevity of various model organisms. In fact, lipids that are closely associated with fat accumulation and obesity can also play crucial roles in regulating aging and longevity. For instance, EGCG prolongs the lifespan of both healthy and high-fat diet-fed obese rats partially by improving lipid metabolism, with activation of fatty acid transport and decrease of levels of total free fatty acids.^193,194^ A recent investigation revealed that the natural phytochemical rotundic acid treats both aging and obesity by inhibiting protein tyrosine phosphatase 1B (PTP1B). This inhibition leads to increased energy expenditure, enhanced brown adipose tissue (BAT) thermogenesis, and improved glucose metabolism in diet-induced obese (DIO) mice. Furthermore, Rotundic acid extends the lifespan of yeast and naturally aged mice, suggesting that PTP1B is a promising target for interventions against aging and obesity.^195^ How lipid metabolism affects longevity has been systematically discussed in recent reviews,^196−198^ and here, we briefly summarize their views and mainly highlight the ways in which phytochemicals regulate lipid metabolism.

Lipids are key biomolecules involved in cellular function and organism metabolism and are usually divided into three categories: fatty acids (FAs), phospholipids, and neutral lipids.^198^ Several studies have revealed that the composition of FAs is related to longevity. For instance, centenarians contain a high ratio of monounsaturated (MU) FA/polyunsaturated (PU) FA, as MUFAs are essential for resistance to peroxidation and can reduce lipid oxidation and damage.^199,200^ High levels of Δ9 desaturase, which transforms saturated FAs into MUFAs, are also found in longer-lived worms.^201^ In addition, two highly conserved longevity-promoting signaling pathways, IIS and mTOR, are associated with lipid metabolism.^202,203^ The activation of mTOR leads to the cessation of lipid catabolic processes, such as autophagy, lipolysis, and β-oxidation.^204^ Moreover, specific enhancement of FOXO activity in *Drosophila* head and peripheral adipose tissue increases lifespan.^205,206^ Consistent with this, the FOXO ortholog DAF-16 promotes lipid degradation and MUFA synthesis in worms, and despite its ubiquitous expression, its activity in adipose tissue is responsible for its longevity effects.^207^ Moreover, SCFAs, produced by the fermentation of plant fibers in the colon, have longevity-extending effects and are also involved in host lipid metabolism.^208^

According to recent studies, we speculate that HSF-1 may be the intersection of lipid metabolism and longevity (Figure 3). HSF-1 is an essential longevity transcription factor that intersects both the IIS and TOR signaling pathways and regulates the expression of a set of HSPs.^209^ Its longevity-promoting effect is thought to promote proteostasis by protecting cells from protein misfolding and aggregation caused by endogenous and exogenous stressors.^210^ A recent study indicated that pharmacological activation of HSF-1 by a pentacyclic triterpenoid named Celastrol elevates energy expenditure in fat tissue through upregulation of PGC-1α, subsequently protecting mice on a high-fat diet (HFD) from obesity and metabolic dysfunction.^211^ Thus, HSF-1 also plays crucial roles in lipid metabolism, thereby affecting lifespan.

**Figure 4:**
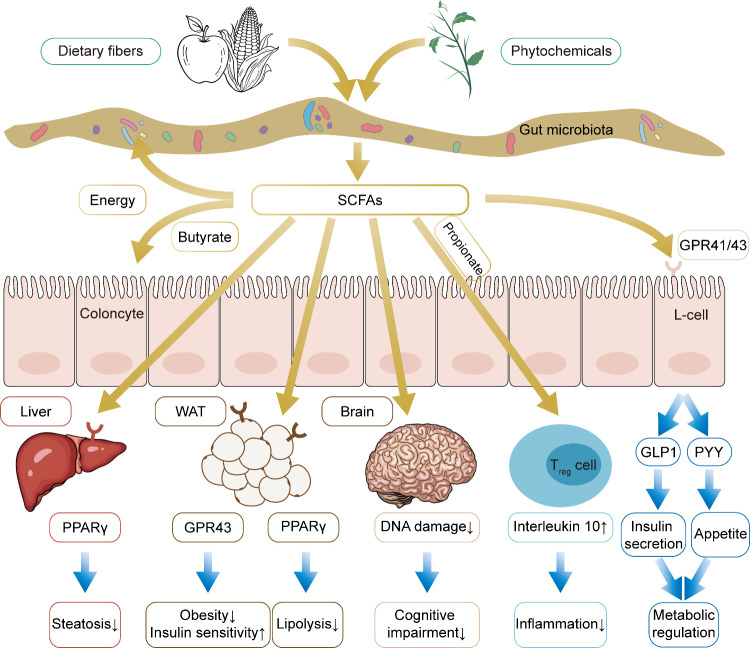
Mechanism linking gut microbiota and lipid metabolism highlights the health benefits of high levels of SCFAs to the host. SCFAs regulate host lipid metabolism by supplying the host with energy, improving peripheral tissue metabolism and stimulating incretin hormone production. SCFAs meanwhile delay brain aging through microbiota-gut-brain axis and regulates T_reg_ cell function, thereby exerting anti-inflammatory effects. These health benefits may ultimately lead to longevity.

Three novel antiaging phytochemicals, namely, dipeptide Tyr-Ala, Ferulic acid, and Glaucarubinone, can reduce fat levels in model organisms,^212,27,50^ suggesting that they may induce a CR effect and/or participate in lipid metabolism. Interestingly, Ferulic acid prolongs the lifespan of worms without significantly affecting their food intake, which means that Ferulic acid does not induce a CR effect.^50^ However, in this process, HSF-1 is required.^50^ Similarly, Tyr-Ala also activates HSF-1 and enhances the expression of heat shock protein 16.2 (HSP 16.2).^27^ Consistent with this, it has been reported recently that the activation of HSF-1 by local hyperthermia therapy (LHT) regulates the browning of white and beige fat by controlling the transcription of the *Hnrnpa2b1* gene, which encodes a protein that can bind to key browning genes, including *Pgc-1α* and uncoupling protein 1 (*Ucp1*), to enhance their mRNA stability^213^ (Figure 3). These two genes are also markers of mitochondrial biogenesis and thermogenesis in mice and human adipocytes, the increased expression of which results in the reduction of lipid accumulation and smaller adipocytes.^214,215^ Therefore, HSF-1-involved lipid metabolism regulation by phytochemicals may depend on PGC-1α and UCP1 (Figure 3). Both heat stress caused by UCP1-mediated thermogenesis in fat tissue and persistent energy overload by lipid oxidation induce mitochondrial ROS overproduction.^216^ UCP1 has been proven to increase mitochondrial proton leakage and inhibit mitochondrial ROS production through a feedback mechanism.^217,218^ Furthermore, elevated PGC-1α levels promote mitochondrial biogenesis and increase antioxidant gene expression through Nrf2 activation.^150^ Therefore, HSF-1 activation may maintain the levels of mitochondrial ROS in an appropriate range by regulating PGC-1α and UCP1, which induces a mitohormesis response, resulting in decreased levels of fat and long-term beneficial effects for cellular and organismal health and longevity (Figure 3).

In summary, these studies suggest a broader, previously unappreciated role for HSF-1 in linking lipid metabolism to longevity. At least 30 phytochemicals summarized in this review can promote longevity by regulating HSF-1 and/or its target genes, although few researchers have measured fat levels that may be affected by HSF-1. Further studies should explore whether phytochemicals that reduce fat levels can promote longevity, considering that there are already several examples.

## 4 Discussion

### 4.1 Gut Microbiota Interacts with Lipid Metabolism

Multiple longevity mechanisms may interact with one another, with phytochemicals playing a crucial role in this process. The phytochemicals we consume can be metabolized by gut microbiota and absorbed by the human body. Therefore, to a certain extent, gut microbiota can influence other longevity pathways, for example, affecting lipid metabolism in the human body. Therapies that target the gut microbiota have been proven to enhance metabolic function in humans. Furthermore, transplanting the fecal microbiota from patients with obesity, steatosis, or type 2 diabetes can partially replicate the donor’s metabolic phenotype in mouse recipients.^219−221^ Lipid metabolism is primarily regulated by nutrients such as sugars and fatty acids. However, several studies have indicated that lipid levels are correlated with the composition of the gut microbiota. Mechanistic links between lipid metabolism and microbial metabolites have also been identified in mouse models.^208^

Research on germ-free (GF) mice exhibit a protective effect against diet-induced obesity due to a combination of several mechanisms. These include an increase in fatty acid oxidation and a decrease in triglyceride deposition in adipocytes when compared to conventionally raised (CONV-R) mice.^222^ Additionally, a lipidomics analysis of GF and CONV-R mice fed a standard chow diet revealed that the gut microbiota impacts lipid composition in host tissues and serum, as well as enhances the clearance of triglycerides from the circulation.^223^ Conversely, the gut microbiota elevates circulating triglycerides, HDL, and total cholesterol levels in mice consuming a high-fat diet.^224^ Comparative studies between CONV-R and GF mice have also indicated that the gut microbiota stimulates hepatic production of MUFA and elongation of PUFA. Furthermore, it was found that acetate produced by the gut microbiota serves as a precursor in hepatic fatty acid synthesis.^225^

Studies in mice administered probiotics further substantiates the role of gut microbiota in modulating host lipid homeostasis. In a study involving mice fed a high-fat high-cholesterol diet, *Lactobacillus curvatus* alone or in conjunction with *Lactobacillus plantarum* was found to decrease cholesterol levels in both plasma and liver. Furthermore, these two strains demonstrated a synergistic effect on hepatic triglycerides.^226^ A similar observation was made in obese rats fed a high-fat diet, where Bifidobacterium spp. reduced circulating triglycerides and LDL levels, while increasing levels of HDL.^227^ Overall, these studies in mouse models underscore the fact that the gut microbiota, in tandem with the diet, plays a significant role in regulating host lipid metabolism and lipid levels in serum and tissues.

SCFAs such as acetate, propionate and butyrate are bacterial metabolites produced through the fermentation of fibers in the colon. These SCFAs play a crucial role in host metabolism, serving as substrates for energy production, lipogenesis, gluconeogenesis, and cholesterol synthesis.^228^ Specifically, butyrate serves as an energy source for colonocytes, while propionate is primarily metabolized by the liver. Beyond their metabolic functions, SCFAs also act as signaling molecules, notably through the G-protein coupled receptors GPR43/FFAR2 and GPR41/FFAR3. For instance, GPR43 has been found to protect against diet-induced obesity in mice.^229,230^ Activation of GPR43 on L-cells leads to an increase in the secretion of glucagon-like peptide-1 (GLP-1),^231,232^ and acetate has been shown to induce antilipolytic activity^233^ and improve glucose and lipid metabolism^229^ through GPR43 in white adipose tissue (WAT). GRP41 has also been implicated in regulating metabolism through its interaction with the gut microbiota. CONV-R *Gpr41* knockout mice exhibit a leaner physique and lower body weight compared to wild-type littermates, a difference that is not observed in GF mice. Additionally, the microbiota enhances peptide YY (PYY) production through GPR41.^234^ Both butyrate and propionate have also been demonstrated to activate PPARγ,^235^ and SCFA-induced activation of PPARγ modulates lipid metabolism by increasing energy expenditure,^236^ reducing body weight, and decreasing liver triglyceride accumulation.^237^

The significance of dietary fibers in shaping gut microbiota composition and function has been the subject of extensive research. Dietary fibers serve as suitable substrates for the bacterial production of SCFAs.^238^ A diet rich in fiber, such as the Mediterranean diet-characterized by regular consumption of fruits, vegetables, cereals, legumes, high intake of olive oil and seafood, and limited consumption of red meat and confectionery-has been linked to a direct increase in intestinal SCFA levels.^239^

Elevated levels of SCFAs are also associated with the promotion of longevity. Dietary oligosaccharides can influence gut microbiota, conferring significant health benefits. A newly discovered functional oligosaccharide, neoagarotetraose (NAT), has been shown to extend the lifespan of naturally aged mice by up to 33.3%. These mice also exhibited improved aging characteristics and reduced damage to cerebral neurons. Following NAT treatment, a significant increase was observed at the gut bacterial genus level (such as *Lactobacillus*, *Butyricimonas*, and *Akkermansia*), along with an increase in SCFAs concentrations in cecal contents.^240^

Some phytochemicals exhibit similar effects. A unique alteration in gut microbiota, mediated by Genistein, was observed through an increase in *Lachnospira* abundance and SCFAs production. Further experiments involving fecal microbiota transplantation and dirty cage sharing suggested that the gut microbiota from Genistein-fed mice could rejuvenate the aging gut and extend the lifespan of progeroid mice. Additionally, Genistein-associated propionate was found to promote the production of regulatory T cell-derived interleukin 10, which in turn alleviated inflammation derived from macrophages.^182^

Overall, a balanced gut microbiota has been demonstrated to positively influence lipid metabolism.^241^ The consumption of dietary fibers, encompassing a variety of plant polysaccharides and oligosaccharides, as well as specific natural phytochemicals, can induce alterations of gut microbiota and elevate the SCFAs levels. These SCFAs play a crucial role in various organs functions and metabolic processes within the host, thereby potentially yielding potential antiaging effects (Figure 4).

### 4.2 Effective Levels and Dietary Intake

Many studies on various model organisms have linked phytochemicals to aging, although our current knowledge of how they act as metabolic interventions to promote longevity in elderly individuals is still in its infancy. While a sizable fraction of the aforementioned studies has mainly focused on polyphenols and their glucosides, emerging evidence suggests that almost all the groups of phytochemicals have potential antiaging effects and deserve attention.

Furthermore, we must emphasize that the antiaging functions of most of the phytochemicals in this review have been tested in yeast, worms, or flies, but they have not been validated in mammals. None of the antiaging phytochemicals described above has been proven in clinical trials to delay the onset or progression of age-associated disorders and the pace of aging. Nevertheless, these substances are chiefly ingested through normal diet, leading to a widespread perception of their safety among humans.

For instance, Quercetin and its various glycosides (QG) are prevalent in fruits and vegetables such as onion, fennel leaves, tea, cranberries, cherries, Tartary Buckwheat, *Capparis spinosa*, among others.^242^ These compounds have been approved for extensive use as food supplements or functional food in Europe and the United States. Studies have demonstrated that oral administration of Quercetin at a dosage of 4 g/day does not induce side effects in humans.^243^ A randomized, double-blind, placebo-controlled crossover clinical trial revealed that a 12-week treatment with Quercetin at a dosage of 500 mg/day could decrease intrahepatic lipid contents, body weight, and body mass index in patients with Nonalcoholic fatty liver disease (NAFLD).^244^

The absorption and metabolism of various food components with quercetin/QG influence the concentration and duration of different Quercetin/QG metabolites in plasma or organs. Onions, rich in Quercetin/QG, have been reported to contain 45 mg Quercetin per 100 g fresh weight.^245^ Dietary intake of 129 g fried onions could provide approximately 13 mg Quercetin.^246^ In a single-blind, diet-controlled crossover study, soup made from 100 g fresh red onion was found to provide 47 mg of Quercetin, while a supplement of 166 mg of Quercetin dihydrate tablet supplement would be equivalent to about 10 mg of Quercetin aglycone derived from onions.^247^ Based on simple calculations, an effective level of 500 mg/d of Quercetin could be achieved through the consumption of approximately 300 g of fried onions or soup made by 64 g frish red onions. Similarly, consuming 130 mg of Quercetin-rich cereal bars resulted in a 5-fold increase in plasma Quercetin concentrations compared to the same amount of Quercetin capsules.^248^

These experiments suggest that Quercetin/QG is dispersed within a solid food matrix, which provides a larger surface area and facilitates its transfer to the absorption site, thereby enhancing bioavailability. Furthermore, it is posited that the daily consumption of Quercetin, rather than supplementation, can potentially achieve effective levels.

### 4.3 Perspective

CR is considered to be the only intervention that promotes longevity in all species investigated. Therefore, some researchers regard phytochemicals as CR mimetics, which refer to pharmaceutical compounds or dietary supplements that produce CR-like effects, without the challenges of maintaining a CR diet.^127^ However, in addition to being CR mimetics, we believe that phytochemicals have at least two other promising advantages: (1) phytochemicals can modulate the composition of the gut microbiota, and (2) they have various targets that may contribute to the synergistic antiaging effects.

(1) Phytochemicals can stimulate the growth of beneficial bacteria while inhibiting the reproduction of intestinal bacterial pathogens. The gut microbiota produces a large number of metabolites that regulate physiological processes in the host, such as immunity, metabolism, and brain functions.^249^ For example, bacterial peptidoglycan plays a role in mediating gut-brain communication via cytosolic Nod-like 2 (Nod2) receptors, subsequently reducing appetite and body temperature of mice.^250^ Given that less food intake and lower body temperature have been linked to a longer lifespan,^251^ it is of great significance to explore how phytochemicals can selectively and feasibly regulate host metabolism and ultimately affect longevity by regulating the composition of the gut microbiota and the production of corresponding metabolites.
(2) It is complicated but important to find the direct molecular targets of phytochemicals. A recent study revealed that PEN2, a component of γ-secretase tethered by the ATP6AP1 subunit, can inhibit v-ATPase activity and then activate lysosomal AMPK when it binds to metformin to explain the antiaging mechanisms of metformin.^133^ However, this field needs further exploration, as the direct molecular targets of most phytochemicals remain unclear, and research on their functional mechanisms is mostly limited to nutrient-sensing pathways. Given the wide range of targets in mitochondria or lipid metabolism-related pathways, it remains to be investigated whether phytochemicals could act on them to affect longevity. Growing evidence has shown that whole foods (fruits, vegetables, legumes, and grains) exhibit stronger health effects than a single pure phytochemical, partially because of the interactive effects of these complex components.^252^ Rapamycin coupled with CR exerts distinct and additive effects in aged skeletal muscle of mice,^253^ suggesting that the known targets may be the cornerstone for the investigation of synergistic effects between phytochemicals and CR or among different phytochemicals.

It is clear that aging can be affected by slight biological stresses such as CR, phytochemicals, and exercise, which may cause global metabolic reprogramming, redistributing nutrients and other resources from anabolism to catabolism. Catabolism switches off cell growth and enhances cytoplasmic turnover, metabolic flexibility, and stress resistance. Anabolism supportes cell growth and proliferation. An accrued calorie intake diet coupled with a sedentary lifestyle cocontributes to an overage of anabolism that leads to insulin resistance, obesity, or metabolic disorder, eventually fueling aging. On the other hand, CR, phytochemicals, and exercise promote catabolism by reducing nutrient intake or increasing energy consumption, which favors a healthy cell or organism.

## Supporting Information Available

Supplementary Table 1 Summary of the antiaging and/or lifespan-extending effects of Saccharides and glycosides. Supplementary Table 2 Summary of the antiaging and/or lifespan-extending effects of Amino acid and peptides. Supplementary Table 3 Summary of the antiaging and/or lifespan-extending effects of Quinones. Supplementary Table 4 Summary of the antiaging and/or lifespan-extending effects of Polyphenols. Supplementary Table 5 Summary of the antiaging and/or lifespan-extending effects of Terpenoids. Supplementary Table 6 Summary of the antiaging and/or lifespan-extending effects of Steroids. Supplementary Table 7 Summary of the antiaging and/or lifespan-extending effects of Alkaloids. Supplementary Table 8 Summary of the antiaging and/or lifespan-extending effects of Others (PDF)
