# Supplementary material for: Unveiling the Longevity Potential of Natural Phytochemicals: A Comprehensive Review of Active Ingredients in Dietary Plants and Herbs
Source: J Agric Food Chem. 2024 Oct 31;72(45):24908–27. doi: 10.1021/acs.jafc.4c07756 (PMC11565747; doi:10.1021/acs.jafc.4c07756)
Supplement: Supplementary file 1 — jf4c07756_si_001.pdf [file jf4c07756_si_001.pdf]

Unveiling the Longevity Potential of Natural Phytochemicals: A Comprehensive Review of Active Ingredients in Dietary Plants and Herbs

Yu Wang[1#], Xiuling Cao[1#], Jin Ma[1], Shenkui Liu[1], Xuejiao Jin[1*], and Beidong Liu[1,2*]

1 State Key Laboratory of Subtropical Silviculture, School of Forestry and Biotechnology, Zhejiang A&F University, Hangzhou 311300, China.
2 Department of Chemistry and Molecular Biology, University of Gothenburg, Gothenburg 41390, Sweden
# These authors contributed equally.
* Correspondence: Xuejiao Jin jinxuejiao1991@cau.edu.cn and Beidong Liu beidong.liu@cmb.gu.se.

Supplementary Table 1 Summary of the anti-aging and/or lifespan-extending effects of Saccharides and glycosides

Type	Active ingredient	Source	Experimental model	Effect	Mechanism	References
Monosaccharide	Sorbitol	*Rosaceae* family	*Caenorhabditis elegans*	①induces an adaptive osmotic response.<br>② prolongs maximum lifespan by about 35%.	① requires the glycerol biosynthetic enzymes glycerol-3-phosphate dehydrogenase (GPDH) -1 and GPDH-2 for the osmotic response.<br>② partially overlapping with CR.	1
	D-Allulose	*Ficus carica*	*Caenorhabditis elegans*	① prolongs mean lifespan both under monoxenic and axenic culture conditions.<br>② does not reduce food intake.	① depends on AAK-2/AMPK.<br>② increases mRNA expression and enzyme activities of SOD and CAT.	2
Polysaccharide	*Astragalus* polysaccharide	*Astragalus*	D-gal-induced	① protects mitochondria by scavenging ROS.	increases activities of CAT, SOD, and GSH-Px.	3

		membranaceus	aging mice	② inhibits mitochondrial permeability transition.		
		Astragalus membranaceus	*Caenorhabditis elegans*	① reduces polyQ aggregation and alleviates associated neurotoxicity.<br>② prolongs maximum lifespan of wild-type and polyQ worms.	depends on DAF-16/FOXO to regulate DAF-16 downstream genes.	4
		Astragalus membranaceus	*Bombyx mori*	① prolongs adult stage and maximum lifespan.<br>② reduces ROS levels.<br>③ increases body weight without affecting food intake and fecundity.	① significantly increases activities of Glutathione S-transferase (GST), SOD, and lysozyme.<br>② balances ER homeostasis by partner-bound immunoglobulin-protein kinase R-like ER kinase (Bip-PERK) signaling pathway.	5
		Astragalus membranaceus	*Drosophila melanogaster*	① prolongs maximum lifespan.<br>② increases reproduction.<br>③ increases locomotor activity.<br>④ increases starvation resistance.<br>⑤ reduces mortality rate induced by hydrogen peroxide.	① increases activities of SOD and CAT.<br>② significantly increases expressions of antioxidant genes including *dFoxO* and *4E−BP*.<br>③ decreases expressions of insulin-like peptides and longevity gene *MTH*.<br>④ depends on insulin/IGF-1 signaling pathway.	6
	Bletilla striata polysaccharide	*Bletilla striata*	*Caenorhabditis elegans*	① prolongs maximum lifespan.<br>② increases its locomotor activity and stress resistance.	① reduces mRNA levels of *age-1* and *hcf-1*.<br>② might depend on *daf-16* through insulin/IGF signaling pathway.	7
	Main root polysaccharide	*Panax notoginseng*	*Caenorhabditis elegans*	① significantly prolongs mean lifespan by 21%.<br>② reduces lipid peroxidation.	increases activities of SOD and CAT.	8
	O-acetyl glucomannan	*Lilium davidii var. unicolor Cotton*	*Caenorhabditis elegans*	① prolongs mean lifespan under normal culture, heat, and oxidative stress conditions.	increases activities of SOD and CAT.	9

				② increases reproduction duration.<br>③ decreases levels of ROS, lipofuscin, and MDA.		
	polysaccharide R-PL	Rose buds	*Drosophila melanogaster*	① prolongs mean and maximum lifespan of female and male fruit flies.<br>② decreases MDA levels.	increases activities of SOD and CAT.	10
	Lycium barbarum polysaccharide LBP, LBP-1, and LBP-3	*Lycium barbarum*	*Drosophila melanogaster*	① prolongs mean lifespan.<br>② decreases MDA levels.<br>③ significantly reduces mortality rate induced by oxidative stress.	① increases activities of SOD and CAT.<br>② at least in part mediated by an age-related signaling pathway (MAPK, TOR, S6K) and expression of longevity genes (*Hep, MTH, and Rpn11*).	11
	Glucomannan hydrolysate	*Amorphophallus konjac*	*Drosophila melanogaster*	① prolongs mean lifespan.<br>② inhibits age-associated hyperproliferation of intestinal stem cells and delays deterioration of gut integrity.	① increases expression of genes encoding antimicrobial peptides.<br>② suppresses both EGFR/MAPK and JAK/STAT signaling pathways.	12
	Yulangsan polysaccharide	*Millettia pulchra*	D-gal-induced aging mice	① increases general appearance of aging mice.<br>② increases interleukin-2 levels while decreases interleukin-6 levels.<br>③ decreases levels of MDA in liver, brain, and serum.<br>④ inhibits advanced glycation end product formation.	① increases activities of antioxidant enzymes.<br>② decreases aging relating genes expression such as *p53* and *p21*.	13
	DCP-1 and DCP-2	*Dicliptera chinensis*	D-gal-induced aging mice	① increases antioxidant capacity, prolongs healthspan.<br>② alleviates inflammatory response.	① decrease activities of LPO and CAT in brain tissue.<br>② increase activities of GSH-Px and SOD.	14

				③ reduces amyloid deposition and levels of MDA.		
	Polysaccharide	*Paris polyphylla*	D-gal-induced aging mice	① increases total antioxidant capacity (TAOC) in serum and liver.<br>② significantly decreases levels of MDA	increases activities of antioxidant enzymes.	15
	Glycoprotein	*Rubus chingii*	D-gal-induced aging mice	① increases SOD and CAT expression in mice kidney and serum.<br>② decreases MDA level.	might by improving klotho gene expression and repairing the renal function.	16
	Angelica polysaccharide	*Angelica sinensis*	D-gal-induced aging mice	① delays aging speed by protecting NSCs (neural stem cell).<br>② decreases levels of IL-1b, IL-6, TNF-a, ROS and MDA.	① increases activity of SOD.<br>② decreases expression of aging-associated genes including *p53* and *p21* through p53 signaling pathway in aging NSCs.	17
	MWP-2	*Malus × micromalus* Makino fruit wine	D-gal-induced aging mice	① inhibits apoptosis of cerebral cortex cells.<br>② decreases levels of MDA in cerebral cortex.	increases activities of SOD, CAT and GSH-Px in cerebral cortex.	18
	Polysaccharide	*Athyrium multidentatum*	D-gal-induced aging mice	attenuates D-galactose-induced oxidative stress and cell apoptosis.	① activates PI3K/Akt/Nrf2 and FOXO3a pathways.<br>② increases and/or inhibits expression of their downstream antioxidants and factors.	19
	Polysaccharide	*Hedysarum polybotrys*	*Drosophila melanogaster*	① prolongs mean lifespan.<br>② ameliorates age-related symptoms such as imbalanced intestinal homeostasis, sleep disturbances, and Aβ induced AD.	mainly by improving antioxidant capacity.	20
	Crude polysaccharide RG70	*Rehmannia glutinosa*	*Caenorhabditis elegans*	① prolongs maximum lifespan.	① depends *daf-16* and *skn-1* and their downstream related genes *sod-3* and *gcs-1*, while decreased the expression of *age-1*.	21

				② increases activities of antioxidant enzymes and reduces accumulation of MDA, ROS and lipofuscin..	② regulate gut microbiota and change abundance of beneficial bacteria.	

Supplementary Table 2 Summary of the anti-aging and/or lifespan-extending effects of Amino acid and peptides

**Type**	**Active ingredient**	**Source**	**Experimental model**	**Effect**	**Mechanism**	**References**
Amino acid	L-theanine	*Camellia sinensis*	*Caenorhabditis elegans*	prolongs maximum lifespan under normal culture and oxidative stress condition.	needs further research.	22
Peptide	Peptides from sesame cake	*Sesamum indicum*	*Caenorhabditis elegans*	① prolongs mean lifespan under normal culture and oxidative stress condition.<br>② decreases ROS levels.	① increases SOD activity.<br>② depends on SKN-1.	23
	Peptides from sesame cake	*Sesamum indicum*	*Caenorhabditis elegans*	① prolongs maximum lifespan under normal culture and oxidative stress condition.<br>② decreases amyloid-β-induced toxicity.	upregulates SKN-1.	24
	Recombinant buckwheat Grx	Buckwheat	*Caenorhabditis elegans*	① prolongs mean lifespan under normal culture and oxidative stress condition.<br>② prolongs healthspan.	upregulates HSF-1.	25
	Recombinant buckwheat trypsin inhibitor	Buckwheat	*Caenorhabditis elegans*	① prolongs maximum lifespan.<br>② decreases Aβ-induced toxicity.	① promotes DAF-16 nuclear localization.<br>② promotes autophagy-lysosomal degradation pathway.	26
	Angelica sinensis peptides	*Angelica sinensis*	*Caenorhabditis elegans*	① prolongs maximum lifespan.<br>② decreases MDA, ROS, and pigments levels.<br>③ does not affect pharyngeal pumping rate.	increases activities of antioxidant enzymes SOD and CAT.	27

	Dipeptide Tyr-Ala	Hydrolyzed maize protein	*Caenorhabditis elegans*	① prolongs maximum lifespan under normal culture, heat, and oxidative stress conditions. ② reduces fat levels. ③ increases locomotor activity.	upregulates genes expression such as *daf-2*, *daf-16*, *sod-3*, *hsp-16.2*, and *skn-1*.	28
	Protein hydrolysates of soybean	*Glycine max*	D-gal-induced aging mice	reverses aging-associated learning and memory impairments.	increases activities of antioxidant enzymes SOD and GSH-px, need further research.	29

Supplementary Table 3 Summary of the anti-aging and/or lifespan-extending effects of Quinones

**Active ingredient**	**Source**	**Experimental model**	**Effect**	**Mechanism**	**References**
Juglone	*Juglans regia*	*Caenorhabditis elegans*	① low concentrations prolong maximum lifespan. ② high concentrations lead to premature death.	① induces hormesis effects. ② depends on DAF-16 and SIR-2.1 andir target genes, such as *hsp-16.2*.	30
Plumbagin	*Iridaceae, Plumbaginaceae, Ebenceae, Drosophyllaceae, Nepenthaceae, Droseraceae.*	*Caenorhabditis elegans*	prolongs mean lifespan.	① depends on both *skn-1* and *daf-16*. ② may induce hormesis effects.	31
Emodin	*Rheum palmatum, Reynoutria japonica, Fallopia multiflora*	*Caenorhabditis elegans*	prolongs maximum lifespan under normal culture and oxidative stress condition.	depends on DAF-16 and SIR-2.1 *via* IIS pathway.	32
Ehretiquinone	*Onosma bracteatum*	yeasts, mammal cells, and mice.	① prolongs replicative lifespan and chronological lifespan of yeast and yeast-like chronological lifespan of mammal cells. ② reduces ROS and MDA levels.	① upregulates *SIR2* genen increases SOD, CAT, and GSH-Px activities. ② increases autophagy activity.	33

Supplementary Table 4 Summary of the anti-aging and/or lifespan-extending effects of Polyphenols

Group	Type	Active ingredient	Source	Experimental model	Effect	Mechanism	References
Phenylpropanoids	phenylpropanoid acid	Chlorogenic acid	Fruits, vegetables, and some TCM	D-gal-induced aging mice	① decreases levels of MDA, TNF-a, and IL-6. ② increases function of liver and kidney.	① increases antioxidant enzymes activities. ②suppresses inflammatory response, need further research.	[34]
				Caenorhabditis elegans	① prolongs maximum lifespan under normal culture, heat, and oxidative stress conditions. ② decreases rate of locomotor activity decline.	① activates DAF-16, HSF-1, SKN-1, and HIF-1. ② depend on DAF-16 *via* IIS pathway.	[35]
		Caffeic acid	Tea, coffee, vegetables, fruits, and herbs	*Caenorhabditis elegans*	① prolongs maximum lifespan under normal culture, heat, and oxidative stress conditions. ② decreases ROS levels. ③ reduces formation of polyQ aggregation. ④ alleviates Aβ-induced toxicity.	① upregulates *hsf-1*, *hsp-16.2*, and *lgg-1* mRNA expression. ② activates DAF-16 and its targets SOD-3 and GST-4	[36]
		Caffeic acid and dihydrocaffeic acid	Tea, coffee, vegetables, fruits, and herbs	*Caenorhabditis elegans*	both increase maximum lifespan under normal culture and heat stress condition.	① upregulate *daf-16* and its genes expression such as *sod-3* and *hsf-1 via* IIS pathway. ② upregulate *sir-2.1* gene expression.	[37]

		6 Caffeoylquinic acids	Tea, coffee, vegetables, fruits, and herbs	*Caenorhabditis elegans*	① all prolong mean lifespan.<br>② 3,5-diCQA increases locomotor activity, pharyngeal pumping rates.<br>③ 3,5-diCQA reduces intestinal lipofuscin levels.<br>④ 3,5-diCQA does not affect body and brood size.	3,5-diCQA inhibits activity of DAF-2, thus upregulates DAF-16 and its target genes expression.	38
		Rosmarinic acid	*Rosmarinus officinalis*	*Caenorhabditis elegans*	prolongs maximum lifespan under normal culture and heat stress condition.	upregulates genes expression such as *daf-16* and *hsp-16.2*.	39
					① prolongs maximum lifespan under normal culture, heat, and oxidative stress conditions. ② reduces fat accumulation.<br>③ decreases glutathione and MDA levels.<br>④ suppresses intestinal lipofuscin accumulation.<br>⑤ does not affect brood size.	① might activate downstream genes expression *via* IIS and MAPK pathways.<br>② might not depend on DAF-16 nuclear localization.	40
		Chicoric acid	*Echinacea purpurea*	*Caenorhabditis elegans*	prolongs maximum lifespan.	activates AMPK pathway.	41
					① prolongs maximum lifespan under normal culture and oxidative stress condition.	partially depends on regulation of *aak-2* and *skn-1*.	42

					② decreases rates of locomotor activity and pharyngeal pumping decline.		
		Ferulic acid	Corn, wheat, and flax	*Caenorhabditis elegans*	① prolongs mean lifespan under normal culture, heat, and oxidative stress conditions.<br>② reduces fat accumulation.<br>③ reduces formation of polyQ aggregation.<br>④ decreases levels of ROS.<br>⑤ increases locomotor activity.	① depends on IIS pathway and mitochondrial signaling pathway.<br>② increases autophagy activity.	43
		Sonneradon A	*Sonneratia apetala*	*Caenorhabditis elegans*	① prolongs maximum lifespan under normal culture and oxidative stress condition.<br>② delays progression of AD.	① regulate mitochondrial function.<br>② regulate IIS pathway.	44,45
	Coumarin	Ferulsinaic acid	*Ferula* genus	*Caenorhabditis elegans*	① prolongs maximum lifespan under normal culture, heat, and oxidative stress conditions. ② decreases both MDA and AGEs (e.g.CML) levels.	might partially depend on expression of genes associated with stress resistance and needs further studies.	46
		Mitophagy-inducing coumarin (MIC)	various edible plants and vegetables	*Caenorhabditis elegans*	① prolongs maximum lifespan and reduces neuropathologies.<br>② improves mitochondrial health.	inhibits ligand-induced activation of nuclear hormone receptor DAF-12/FXR, which in turn induces mitophagy.	47

	Lignan	Sesamin	*Sesamum indicum*	*Drosophila melanogaste*	① prolongs mean lifespan by 12 %.<br>② alleviates neurodegeneration.	partially depends on regulation of genes SOD1, SOD2, CAT, and Rpn11.	48
				Caenorhabditis elegans	① prolongs maximum lifespan under normal culture and oxidative stress condition.<br>② does not affect body and brood size.	① partially depends on activation of both *skn-1* and *daf-16*.<br>② might suppress TORC1 without inducing side effects.	49
					prolongs maximum lifespan.	depends on SIRT1, AMPK, TOR, and IIS pathways.	50
		Sesamin,sesamolin, and sesamol	*Sesamum indicum*	*Caenorhabditis elegans*	① Sesamin prolongs maximum lifespan.<br>② Both Sesamin and Sesamolin produce protective activity against Aβ toxicity	may ameliorate Aβ toxicity by inhibiting Aβ oligomerization.	51
		Arctigenin, Matairesinol, Arctiin, Isolappaol A, Lappaol C, and Lappaol F	*Arctium lappa*	*Caenorhabditis elegans*	all can prolong mean lifespan under normal culture and oxidative stress condition.	all upregulate expression of *jnk-1*, and upregulate DAF-16 *via* JNK-1 pathway.	52
		Nectandrin B	*Myristica fragrans*	*Drosophila melanogaste*	① prolongs healthspan and maximum lifespan.<br>② improves age-related symptoms including locomotor activity deterioration, body weight gain, eye degeneration, and neurodegeneration.	not mentions.	53

		Magnolol	*Magnolia ofcinalis*	*Caenorhabditis elegans*	① prolongs maximum lifespan under normal culture, oxidative, and heat stress conditions.<br>② reduces ROS and MDA levels, and increases SOD and CAT activities.<br>③ improves the age-related neurodegeneration in PD and AD models.	depends on IIS and *sir-2.1* pathways.	54
Flavonoids	Flavone	Baicalein	*Scutellaria baicalensis*	*Caenorhabditis elegans* and Hct116 human colon carcinoma cells	① prolongs maximum lifespan of worm.<br>② decreases ROS levels in Hct116 cells.	① upregulates SKN-1/Nrf2 signaling pathway in worms.<br>② promotes Nrf2 nuclear localization.<br>③ increases expression of heme oxygenase-1 in Hct116 cells.	55
		Apigenin	fruits, vegetables, beans and tea.	D-gal-induced aging mice	① rescues aging-related changes such as behavioral impairment, decreased organic index, histopathological injury, increased SA-β-gal activity and AGEs level.<br>② decreases MDA level.	May increase activities of antioxidant enzymes SOD and CAT through activating Nrf2 pathway and downstream gene targets,<br>including HO-1 and NQO1.	56
		Chrysin, 6-hydroxyflavone, and Baicalein	Fruits and vegetables	*Caenorhabditis elegans*	respectively prolongs mean lifespan by up to 8.5%, 11.8% and 18.6%.	① Chrysin depends on DAF-16.	57

						② both Baicalein and 6-hydroxyflavone depend on SKN-1 pathway.	
		5′-Hydroxy-6, 7, 8, 3′, 4′-pentamethoxyflavone	*Murraya paniculata*	*Caenorhabditis elegans*	①prolongs maximum lifespan under normal culture and oxidative stress condition.<br>② reduces ROS levels, and brood size.	① upregulates CR pathway thus activates autophagy in *eat-2*/*let-363*/*pha-4*-dependent manner.<br>② also depends on SKN-1.	58
		Tectochrysin	*Alpinia oxyphylla*	*Caenorhabditis elegans*	① prolongs maximum lifespan under normal culture, oxidative, and heat stress conditions.<br>② decreases rate of locomotor activity decline.<br>③ protects against Aβ1-42-induced toxicity.	① upregulates DAF-16 and its target genes expression.<br>② requires DAF-16 and HSF-1.	59
		Nobiletin	*Citrus genus*	*Caenorhabditis elegans*	① prolongs maximum lifespan under normal culture, heat, ultraviolet radiation, and oxidative stress conditions.<br>② decreases rate of locomotor activity decline.<br>③ decreases ROS levels.	upregulates DAF-16, HSF-1, SKN-1. and their target genes expression.	60
		Acacetin	*Vachellia farnesiana*	D-gal-induced aging mice	rescues increased serum AGEs, myocardial telomere length shortening, increased protein expression of p21 and p53, and	activates Sirt1-mediated Sirt6/AMPK signaling pathway.	61

					reduced mitophagy signaling proteins PINK1/Parkin and Sirt6 expression in aging mice.		
		Tangeretin	*Citrus genus*	*Caenorhabditis elegans*	① prolongs maximum lifespan under normal culture and heat stress condition.<br>② decreases rate of locomotor activity decline.<br>③ does not affect pharyngeal pumping rate and brood size.	regulates genes expression of IIS pathway and *hsp* family.	62
		Morusin and Mulberrin	*Morus alba*	*Saccharomyces cerevisiae* and *Caenorhabditis elegans*	① both prolong chronological lifespan of yeast.<br>② prolongs healthspan and maximum lifespan of worm.	① at least partially targets *SCH9* in yeast.<br>② depends on full functions of genes *akt-1* or *akt-2*.	63
	Flavonol	Quercetin	Fruits, vegetables, wines and teas.	*Caenorhabditis elegans*	① prolongs maximum lifespan.<br>② decreases rate of locomotor activity decline.<br>③ decreases both intercellular and mitochondrial ROS levels.	① upregulates DAF-16 and SKN-1 *via* IIS and MAPK pathway.<br>② upregulates HSF-1 expression and activity.	64
		Quercetin、Isorhamnetin and Tamarixetin	*Hippophae rhamnoides*	*Caenorhabditis elegans*	① all prolong lifespan under normal culture, heat, and oxidative stress conditions.<br>② Quercetin increases body and brood size.	needs further studies.	65
		Myricetin	*Myricaceae, Polygonaceae,*	*Caenorhabditis elegans*	① prolongs mean lifespan.<br>② decreases lipofuscin levels.	① promote DAF-16 nuclear localization.	66

			Primulaceae, Pinaceae, Anacardiaceae.				
		Tambulin	*Zanthoxylum armatum*	*Caenorhabditis elegans*	① prolongs maximum lifespan under normal culture and oxidative stress condition.<br>② reduces lipofuscin and protein carbonyl.<br>③ increases locomotor activity and pharyngeal pumping rate.	① promotes DAF-16 nuclear localization.<br>② partially *via* IIS pathway.	67
		Fisetin	Fruits and vegetables	naturally aged mice	① prolongs median and maximum lifespan.<br>② restores tissue homeostasis.<br>③ reduces age-related pathology.<br>④ prolongs healthspan.	① reduces percent of senescent cells in *vivo*.<br>② reduces expression of senescence markers in multiple organs, needs further studies.	68
	Flavanone	Naringenin	Grapefruit and tomato	*Drosophila melanogaster*	① prolongs mean lifespan in a dose-dependent manner.	needs further studies.	69
		Hesperetin	*Camellia sinensis* and *Citrus* fruits	naturally aged mice	① prolongs median and maximum lifespan.<br>② prolongs healthspan.	activates Cisd2 and enhances its expression.	70
	Flavanonol	2,3-dehydrosilybin A/B	*Silybum marianum*	*Caenorhabditis elegans*	① prolongs maximum lifespan under normal culture and oxidative condition.<br>② increases resistance to aggregation-related proteotoxic stress.	① depends on facilitative glucose transporter isoform (FGT)-1 and DAF-16.<br>② inhibits FGT-1 and promotes DAF-16.	71

		Dihydromyricetin	*Ampelopsis grossedentata*	*Drosophila melanogaster*	① prolongs maximum lifespan.<br>② increases stress tolerance, lipid levels, and climbing ability.<br>③ slows down gut dysfunction.	① downregulates AKT and ERK signaling.<br>② increases activity of FOXO and Anterior open (AOP).<br>③ increases autophagy activity.	72
		Taxifolin	Fruits and herbs	D-gal-induced aging mice	① restores spatial learning and memory impairment.<br>② reverses cholinergic dysfunction incited by long-term D-gal treatment.<br>③ mitigates oxidative stress injury by decreasing levels of ROS and MDA.	① increases activities of antioxidant enzymes.<br>② decreases apoptosis of aging brain by regulating phosphorylation levels of PI3K, AKT.<br>③ upregulates Nrf2, nuclear heme oxygenase (HO)-1, and NADH dehydrogenase quinone (NQO) 1 to moderate oxidative stress injury.<br>④ regulates composition of gut microbiota and abundance of beneficial bacteria.	73
	Isoflavone	Genistein	*Vigna angularis*	*Caenorhabditis elegans*	① prolongs maximum lifespan under normal culture, heat, and oxidative conditions.<br>② increases locomotor activities.	increases expressions of stress resistance proteins such as SOD-3 and HSP-16.2.	74

					③ does not affect pharyngeal pumping rate, brood and body size.		
				Naturally aged mice and *Zmpste24*$^{-/-}$ progeroid mice	① prolongs healthspan and lifespan.<br>② modulates homeostasis of aging gut.<br>③ promotes regulatory T cell-derived interleukin 10 production, which alleviates macrophage-derived inflammation.	increases *Lachnospira* abundance and short-chain fatty acid (SCFA) production.	75
		Calycosin	*Astragalus mongholicus*	*Caenorhabditis elegans*	① prolongs maximum lifespan under normal culture and oxidative stress condition.<br>② decreases ROS level.	① promotes DAF-16 nuclear localization *via* IIS pathway.<br>② upregulates DAF-16 target genes such as *sod-3*, *ctl-1*, and *hsp-16.2*.	76
		Prunetin	*Trifolium pratense*	*Drosophila melanogaster*	① prolongs maximum lifespan.<br>② increases locomotor activity.	① depends on activation of AMPK.<br>② upregulates *Sir2*.<br>③ results in feminization of males in flies.	77
		Corylin	*Psoralea corylifolia*	Yeast and high-fat fed mice	① prolongs replicative lifespan of yeast.<br>② prolongs lifespan of aged HFD-fed mice.	① targets Gtr1 protein in yeast.	78

		Puerarin	*Puerariae lobatae*	*Drosophila melanogaster*	① prolongs healthspan and maximum lifespan.<br>② increases ATP content.<br>③ promotes autophagy.	① upregulates expression of Shaker, CAT, and SOD1, and downregulates PARP-1 and HSP70.<br>② increases AMPK levels.<br>③ promotes LAMP1, and elevates levels of autophagy-related genes, including ATG1, ATG5, and ATG8b.	79
	Isoflavanone	Boeravinone B	*Boerhavia diffusa*	*Caenorhabditis elegans*	① prolong maximum lifespan under normal culture and oxidative stress condition.<br>② decreases ROS levels.<br>③ decreases brood size, and pharyngeal pumping rate.	① promotes DAF-16 nuclear localization.<br>② upregulates SKN-1.<br>③ might affect feeding behavior and induce CR effects.	80
	Chalcone	Xanthohumol	*Humulus lupulus*	*Drosophila melanogaster*	① prolongs mean lifespan under normal culture, heat, starvation, acetic acid-induced, cold, and oxidative stress conditions.<br>② increases locomotor activity.	increases antioxidant enzyme activities, needs more studies.	81
		4,4′-dimethoxychalcone (DMC)	*Angelica keiskei*	yeast, worms, flies, human cell, and mice	① prolongs maximum lifespan of yeast, worms and flies.<br>② decelerates senescence of human cell cultures.<br>③ protects mice from prolonged myocardial ischaemia.	① depends on specific GATA transcription factors.<br>② increases autophagy activity.	82

		Butein	*Spatholobus suberectus*	*Caenorhabditis elegans*	① prolongs maximum lifespan.<br>② improves the age-related neurodegeneration in PD and AD models.	depends autophagic protein BEC-1 and transcription factor DAF-16.	83
	Aurone	Damaurone D	The damask rose	*Caenorhabditis elegans*	① prolongs maximum lifespan under normal culture, heat, osmotic, and oxidative conditions.<br>② decreases food intake, body length, and lipofuscin level.<br>③ decreases rate of locomotor activity decline.	① upregulates expressions of stress-response proteins such as SOD-3 and HSP-16.2.<br>② promotes DAF-16 nuclear localization<br>③ induces CR effects.	84
		Hispidol	*Glycine max*	*Caenorhabditis elegans*	① prolongs maximum lifespan under normal culture and heat stress condition.<br>② decreases lipofuscin level.<br>③ decreases rate of pharyngeal pumping.<br>④ increases locomotor activity.	① promotes DAF-16 nuclear localization.<br>② upregulates expressions of HSP-16.2.<br>③ may induce CR effects.	85
	Homo-isoflavone	Sappanone A	*Caesalpinia sappan*	*Caenorhabditis elegans*	① prolongs maximum lifespan under normal culture and heat stress condition.<br>② increases locomotor activity.<br>③ reduces ROS and lipofuscin levels.<br>④ does not affect pharyngeal pumping rate and brood size.	① upregulates gene expression of *daf-16* and *hsp-90*.<br>② promotes DAF-16 nuclear localization *via* IIS pathway.	86

		Brazilin	*Caesalpinia sappan*	*Caenorhabditis elegans*	① prolongs maximum lifespan under normal culture, heat, osmotic, and oxidative stress conditions.<br>② decreases ROS level.<br>③ does not affect food intake, body, and brood size.	increases activities of stress resistance proteins such as HSP-16.2 and SOD-3, needs further study.	87
	Flavonolignan	Silymarin	*Silybum marianum*	*Caenorhabditis elegans*	① prolongs healthspan and maximum lifespan under normal culture and oxidative stress condition.<br>② reduces proteotoxicity mediated paralysis phenotype in Alzheimer's disease models.	Not mentioned.	88
Phenolic glycoside	-	Polydatin	*Reynoutria japonica*	*Caenorhabditis elegans*	① prolongs maximum lifespan under normal culture and acute stress condition.<br>② increases oxidative stress resistance.	① increases activities of protein SOD-3.<br>② increases *daf-16* expression at protein and mRNA levels in IIS pathway.	89
				Male Sprague−Dawley rats and male Kunming mice	decreases levels of MDA.	promotes antioxidant enzyme activities of total T-SOD, CAT and GSH-Px in plasma.	90
		Rutin	Citrus fruits and buckwheat seeds	*Drosophila melanogaster*	prolongs median lifespan of flies in a dose dependent manner.	may have a negative regulatory effect on IIS and Tor pathway.	91

				Caenorhabditis elegans	① prolongs maximum lifespan under normal culture and oxidative stress condition.<br>② reduces polyQ protein aggregation in muscle.	① regulate IIS pathway including DAF-16 and its downstream genes. ② increases autophagy activity.	92
		Icariside II	*Epimedium brevicornu*	*Caenorhabditis elegans*	① prolongs maximum lifespan under normal culture, heat, and oxidative stress conditions.<br>② decreases rate of locomotor activity decline in late adulthood.<br>③ ameliorates protein aggregation.	depends on DAF-2, DAF-16, and HSF-1 *via* IIS pathway.	93
		Icariin	*Epimedium brevicornu*	C57BL/6 mice	① prolongs healthspan and mean lifespan.<br>② decreases levels of MDA.<br>③ maintains genomic stability.	① increases activities of antioxidant enzymes.<br>② downregulates expression of genes related to DNA damage response.	94
		Phloridzin	Branches of dwarf apple JM7	K6001 yeast	prolongs replicative lifespan.	① significantly increases *SOD1*, *SOD2*, and *SIR2* genes expression.<br>② increases activities of SOD and SIR2.	95
		Hesperidin	*Citrus genus*	K6001 yeast	① prolongs replicative lifespan.<br>② increases tolerance to ROS.	① increases *SOD* gene expression and SIR2 activity.<br>② inhibits *UTH1* gene expression.	96

				Caenorhabditis elegans	① prolongs maximum lifespan under normal culture and oxidative stress conditions.. ② protects against Aβ-induced toxicity.	depends on *acr-16* and autophagy genes *lgg-1* & *bec-1*.	97
		Parishin	*Gastrodia elata*	K6001 yeast	① prolongs replicative lifespan. ② increases survival rate under oxidative stress condition. ③ significantly decreases ROS and MDA levels.	① increases *SIR2* gene expression and SOD activity. ② inhibits downstream genes expression of TOR signaling pathway.	98
		Bis (4-hydroxybenzyl) ether mono-β-L-galactopyranoside	*Gastrodia elata*	K6001 and YOM36 yeast	① prolongs replicative lifespan of K6001 and chronological lifespan of YOM36 yeast strains. ② increases survival rate of yeast under oxidative stress condition. ③ reduces ROS and MDA levels.	① increases *SIR2*. ② decreases *UTH1* genes expression. ③ increases CAT and GSH-Px activities.	99
		Neohesperidin	*Citrus genus*	BY4742 yeast	prolongs chronological lifespan in a concentration-dependent manner.	may due to decrease of ROS, needs further studies.	100
		Pentagalloyl glucose	*Eucalyptus genus*	*Caenorhabditis elegans*	① prolongs maximum lifespan. ② does not affect pharyngeal pumping rate and brood size.	may regulate IIS pathway, CR, SIR-2.1/SIRT signaling pathways, and mitochondrial electron transport chain.	101
		Acacetin 7-O-α-l-rhamnopyranosyl (1–2) β-D-xylopyranoside	*Premna serratifolia*	*Caenorhabditis elegans*	① prolongs maximum lifespan under normal culture, heat, and oxidative stress conditions. ②	① affects bacterial metabolism and in turn leads to CR.	102

					decreases lipofuscin and intracellular ROS levels.	② partially depends on DAF-16.	
		2,3,5,4'-Tetrahydroxystilbene 2-O-β-D-glucoside	*Polygonum multiflorum*	*Caenorhabditis elegans*	① prolongs mean lifespan under normal culture and heat stress condition.<br>② decreases aging pigment lipofuscin levels.	decreases expression of antioxidative enzymes SOD-3 and GST-4 under oxidative stress condition.	103
		Vitexin	*Vigna angularis*	*Caenorhabditis elegans*	① prolongs maximum lifespan under normal culture, heat, and oxidative stress conditions.<br>② decreases ROS levels.	① increases antioxidant enzyme activities such as SOD and CAT.<br>② increases stress resistance proteins SOD-3 and HSP-16.2 expressions.	104
		Vitexin and Isovitexin	*Vigna angularis*	*Caenorhabditis elegans*	① both prolong and maximum lifespan.<br>② both rescue the declines of pharyngeal pumping and body bending rates, and the increase of intestinal lipofuscin accumulation.	might inhibit IIS by binding to the crucial amino acid residue ARG1003 in the pocket of IGFR.	105
		Verminoside	*Stereospermum suaveolens*	*Caenorhabditis elegans*	① prolongs maximum lifespan under normal culture, heat, and oxidative stress conditions. ② decreases ROS levels.<br>③ does not affect pharyngeal pumping rate.	① increases expression of SOD-3.<br>② promotes DAF-16 nuclear localization.<br>③ depends on IIS pathway.	106

		Echinacoside	*Cistanche deserticola*	*Caenorhabditis elegans*	① prolongs maximum lifespan under normal culture, heat, and oxidative stress conditions. ② does not affect pharyngeal pumping rate and brood size.	① increases genes expression of *daf-16* and its target genes such as *sod-3*, *hsp-16.2*, and *ctl-1*. ② promotes DAF-16 nuclear localization.	107
		Arbutin	*Arctostaphylos uva-ursi*	*Caenorhabditis elegans*	① prolongs mean lifespan under normal culture, heat, and oxidative stress conditions. ② decreases ROS levels. ③ does not affect brood size.	① increases genes expression of *daf-16* and its target genes such as *sod-3* and *hsp-16.2*. ② promotes DAF-16 nuclear localization. ③ might depend on DAF-16 pathways.	108
		Naringin	*Citrus genus*	*Caenorhabditis elegans*	① prolongs maximum lifespan under normal culture, heat, and oxidative stress conditions. ② decreases lipofuscin levels. ③ decreases rate of locomotor activity decline. ④ delays progression of Alzheimer's and Parkinson's diseases.	① increases genes expression of *daf-16* and its target gene. ② promotes DAF-16 nuclear localization. ③ depends on IIS pathway.	109
				Drosophila melanogaster	① prolongs maximum lifespan. ② enhances abilities of climbing and resistance to stress.	may depend on inhibition of IIS pathway and activation of autophagy pathway.	110

					③ improves the activities of antioxidant enzymes, and decreases MDA content.		
		Secoisolariciresinol diglucoside	*Linum usitatissimum*	*Caenorhabditis elegans*	① prolongs maximum lifespan under normal culture, heat, and oxidative stress conditions. ② decreases rate of locomotor activity decline.<br>③ decreases toxicity of Aβ protein.<br>④ inhibits adipogenesis.	① might depend on DAF-16 and HSF-1 to upregulateir target genes<br>② inhibits adipogenesis by activating AMPKα.	111
		Trilobatin	*Lithocarpus polystachyrus*	*Caenorhabditis elegans*	① prolongs mean lifespan under normal culture, heat, and oxidative stress conditions.<br>② significantly decreases ROS and MDA levels.<br>③ does not affect brood size.	① promotes DAF-16 nuclear localization.<br>② increases expression of DAF-16, SIRT3, and SKN-1.<br>② at least partially depends on SKN1/SIRT3/DAF16 signaling pathway.	112
		Orientin	Nasturtium, bamboo leaves, and black fern	*Caenorhabditis elegans*	① prolongs maximum lifespan under normal culture, heat, and oxidative stress conditions. ② decreases accumulation of toxic proteins.<br>③ prolongs healthspan.	① regulates AMPK, DAF-16, SKN-1, and HSF-1.<br>② increases autophagy activity.	113
		Tiliroside	*Platanus orientalis*	*Drosophila melanogaster*	① prolongs maximum lifespan.	① activates proteostatic genes.	114

					② decreases ROS levels in somatic tissues. ③ decreases rate of locomotor activity decline.	② increases proteasome and lysosomal cathepsin activities.	
		Acteoside	*Osmanthus fragrans*	D-gal-induced aging mice	① exhibits neuroprotective activities.<br>② provides protection against DNA damage.<br>③ inhibits oxidative damage.	① increases levels of Nrf-2 and antioxidant enzyme activities of GSH-Px.<br>② decreases aging-related enzyme activity of monoamine oxidase (MAO).	115
		Tetrahydroxystilbene glucoside	*Fallopia multiflora*	SAMP8 mice	① prolongs maximum lifespan.<br>② significantly increases memory ability.	① increases protein levels of neural klotho.<br>② depends on IIS pathway in brain.	116
		XAp-G	*Capsicum frutescens*	*Caenorhabditis elegans*	① prolongs maximum lifespan under normal culture, and oxidative stress condition.<br>② decreases ROS levels.	depends on IIS pathway.	117
		Isoquercitrin	*Apocynum venetum*	K6001 yeast	① prolongs replicative and chronological lifespan.<br>② increases survival rate under oxidative and heat stress.<br>③ decreases ROS and MDA levels.	depends on Sch9/Rim15/Msn signaling pathway	118
		Rosavin	*Rhodiola rosea*	*Caenorhabditis elegans*	① prolongs maximum lifespan under normal culture, heat, and oxidative stress conditions.	activates the IIS pathway by downregulating the upstream components daf-2 and age-1.	119

					② decreases both MDA and ROS levels.		
		Cynaroside	many medical plants and vegetables	*Caenorhabditis elegans*	① prolongs healthspan and maximum lifespan.<br>② improves neurodegeneration diseases such as Alzheimer's and polyglutamine disease.	depends on IIS pathway.	120
Other polyphenols	Diarylheptanoid	Curcumin	*Curcuma longa*	*Drosophila melanogaster*	① prolongs maximum lifespan under normal culture and oxidative stress condition.<br>② increases locomotor activity.	① regulates expression genes such as *mth*, *thor*, *InR*, and *JNK*.<br>② downregulates expression of proteins in insulin, JNK, and methuselah signaling pathways.	121
				Caenorhabditis elegans	① prolongs maximum lifespan.<br>② decreases ROS and lipofuscin levels.<br>③ decreases body size and pharyngeal pumping rate.<br>④ does not affect brood size.	at least in part by shifting metabolism to a CR-like state that is modulated by SIR-2.1 and SKN-1.	122
				Drosophila melanogaster	① prolongs maximum lifespan under normal culture and oxidative stress condition.<br>② decreases MDA and lipid peroxidation levels.<br>③ increases locomotor activity.	regulate stress responsive genes such as antioxidant enzyme SOD.	123

				Drosophila melanogaster	prolongs mean lifespan.	① increases SOD activity while does not enhance SOD gene expression.<br>② downregulates acetylcholinesterase (AChE) mRNA expression, which in turn inhibits AChE activity.	124
				Saccharomyces cerevisiae	① prolongs chronological and replicative lifespan in dose-dependent manner,<br>② promotes aging of yeast cells lacking antioxidant enzymes and DNA repair mechanisms.	may induce hormesis effects.	125
	Stilbenoid	Resveratrol	*Vitis* genus	*Caenorhabditis elegans*	prolongs maximum lifespan.	increases autophagy activity through activation of SIR-1 and depend on an essential autophagic modulator Beclin-1.	126
				Annual fish *Nothobranchius guentheri*	① prolongs maximum lifespan.<br>② increases cognitive ability and locomotor activity.<br>③ decreases aging-related histological markers such as lipofuscin formation and expression of SA-β-Gal.<br>④ does not affect body size.	not mentions.	127

				Apis mellifera	① prolongs mean, maximum, and median lifespan.<br>② affects gustatory responsiveness.	may depend on CR effects caused by reduction of food consumption.	128
		Resveratrol, Oxyresveratrol	*Artocarpus lakoocha*	*Caenorhabditis elegans*	both prolong maximum lifespan.	induces CR effects that depend on SIR-2.1 - and AMPK pathway.	129
		Resveratrol	*Vitis* genus	*Caenorhabditis elegans*	① prolongs lifespan.<br>② prevents age-associated loss of mtotic germ cells, brood size, and reproductive span.	depends on SIR-2.1/DAF-16 and MAPK-1/SKN-1 pathway while both are independent.	130
		Piceatannol	Passion fruit seed	*Caenorhabditis elegans*	① prolongs maximum lifespan under normal culture, heat, and oxidative stress conditions.<br>② decreases rate of locomotor activity decline.<br>③ decreases pharyngeal pumping rate.<br>④ does not affect growth rate, body, and brood size.	① promotes DAF-16 nuclear localization.<br>② depends on IIS and SIR-*2.1*-dependent pathways.	131
	Hydrolysable tanin	Tannic acid	Tea, red wine, grapes, and nuts	*Caenorhabditis elegans*	① prolongs mean lifespan under normal culture and heat stress condition.<br>② does not affect brood size and pharyngeal pumping rate.	induces mitohemesis effects and depends on MAPK-1.	132

		Epigallocatechin gallate	*Camellia sinensis*	Wistar rats	① increases median lifespan and delays death by approximately 8–12 weeks in healthy rats .<br>② reduces liver and kidney damage and improves age-associated inflammation and oxidative stress	① activates longevity factors FoxO3a and SIRT1.<br>② decreases mRNA and protein expressions of transcription factor NF-κB.	133
					① prolongs healthspan and lifespan of obese rats.<br>② reduces serum glucose, serum lipids.<br>③decreases serum-free fatty acid levels but increases N-3 FFA levels.	① increases protein expression of FOXO1, Sirt1, CAT, FABP1, GSTA2, ACSL1 and CPT2 in livers.<br>② decreases NF-κB, ACC1 and FAS protein levels in livers.	134
				Caenorhabditis elegans	prolongs mean lifespan in an inverted U-shaped dose-response manner.	① induces mitohemesis effects.<br>② triggers mitochondrial biogenesis.<br>③ depends on AMPK/SIRT1/FOXO pathway.	135
		Epigallocatechin gallate and epicatechin gallate	*Camellia sinensis*	*Caenorhabditis elegans*	increases mean lifespan, fitness, and stress resistance when applied at low doses.	① induces mitohemesis effects.<br>② activates SKN-1 and DAF-16 and downstream SOD and	136

						CAT activities. ③ depends on AMPK, SIRT1, and MAPK.	
		Ellagic acid	Strawberry, blackcurrant, pomegranate, walnut, and grapes	*Drosophila melanogaster*	prolongs maximum lifespan under normal culture, heat, starvation, and oxidative stress conditions.	① upregulates expressions of CAT, dFOXO, ATG1, and SOD2 in male flies<br>② upregulates expressions of dFOXO, CAT, and SOD2 in female flies.	137
		Protocatechuic acid	*Veronica peregrina*	*Caenorhabditis elegans*	① prolongs maximum lifespan under normal culture, heat, osmotic, and oxidative stress conditions.<br>② decreases ROS levels.<br>③ decreases pharyngeal pumping rate and brood size.	① upregulates antioxidant enzyme activities.<br>② might induce CR effects.	138
		Methyl 3,4-Dihydroxybenzoate	*Hedyotis diffusa*	*Caenorhabditis elegans*	① prolongs maximum lifespan under normal culture and oxidative stress condition.<br>② decreases rate of locomotor activity decline.<br>③ does not affect in fat storage, pharyngeal pumping rate, and brood size.	① at least partially regulates *daf-2*/*daf-16*.<br>② promotes DAF-16 nuclear localization	139
		3,4-dihydroxybenzaldehyde	*Sasa senanensis, Pinellia ternata*	*Drosophila melanogaster*	prolongs mean lifespan of female.	might inhibit 2-oxoglutarate binding sites of prolyl 4-	140

						hydroxylase then downregulate IIS pathway.	
	Condensed tannins	Catechin, Epicatechin, 3'-O-methylepicatechin and 4'-O-methylepicatechin	*Acacia catechu*	*Caenorhabditis elegans*	① all increases resistance against heat and oxidative stress. ② only two methylated epicatechin derivatives prolong mean lifespan.	need further studies.	141
		Catechin	*Acacia catechu*	*Caenorhabditis elegans*	① prolongs maximum lifespan. ② decreases rate of locomotor activity decline.	upregulates autophagy-related genes such as *bec-1* and *pink-1* to activate mitophagy.	142
		Epicatechin	*Camellia sinensis*	*Drosophila melanogaster*	prolongs mean lifespan.	needs further studies.	143
				Naturally aged mice	① increases survival rate. ② attenuates deterioration of skeletal muscles. ③ protects against decline in nicotinate and nicotinamide metabolism.	reverses age-related changes in genes associated with extracellular matrix and PPAR pathways and protein expression in skeletal muscle.	144
		Procyanidin B2	widely distributed in plants and daily foods	D-gal-induced aging mice	possesses potential prevention of cognitive and oxidative impairment.	may through regulate composition of gut microbiota.	145
		Procyanidin C1	*Vitis* genus	Naturally aged mice	① prolongs healthspan and maximum lifespan of mice through its action on senescent cells.	possibly to mediate senotherapeutic activity by promoting production of ROS and mitochondrial dysfunction of senescent cell.	146

					② inhibits SASP formation at low concentrations.<br>③ selectively kills senescent cells at higher concentrations.		
	Monomethoxybenzene	6-shogaol	*Zingiber officinale*	*Caenorhabditis elegans*	① prolongs maximum lifespan under normal culture, osmotic, heat, and oxidative stress conditions.<br>② does not affect pharyngeal pumping rate, body and brood size.	increases SOD-3 and HSP-16.2 expressions, needs further studies.	147
	Cannabinoid	Cannabidiol	*Cannabis sativa*	*Caenorhabditis elegans*	prolongs maximum lifespan and increases neuronal health.	depends on autophagy and requires SIRT1.	148

Supplementary Table 5 Summary of the anti-aging and/or lifespan-extending effects of Terpenoids

**Type**	**Active ingredient**	**Source**	**Experimental model**	**Effect**	**Mechanism**	**References**
Hemiterpene	Prenol	*Citrus* genus	*Caenorhabditis elegans*	① prolongs maximum lifespan under normal culture and oxidative stress conditions.<br>② delays amyloid-β induced paralysis.<br>③ reduces α-synuclein aggregation and ROS levels.	increases expression of DAF-16, HSF-1, and SKN-1 and their target genes.	149
Iridoid	4-hydroxy-E-globularinin	*Premna integrifolia*	*Caenorhabditis elegans*	① prolongs mean lifespan under normal culture and oxidative stress	① promotes DAF-16 nuclear.<br>② localization increases DAF-16	150

				condition.<br>② decreases ROS levels and fat accumulation.	target genes such as *hsp-16.2* and *sod-3*.	
	10-O-trans-p-Coumaroylcatalpol (OCC)	*Premna serratifolia*	*Caenorhabditis elegans*	① prolongs mean lifespan.<br>② decreases ROS levels and α-synuclein aggregation.	promotes DAF-16 nuclear localization.	151
	Catalpol	*Rehmannia glutinosa*	*Caenorhabditis elegans*	① prolongs maximum lifespan under normal culture, heat, and oxidative stress conditions.<br>② increases locomotor activity,<br>③ decreases pharyngeal pumping rate and lipofuscin levels.	① promotes DAF-16 nuclear localization.<br>② depends on IIS and JNK pathway then activates DAF-16 and SKN-1. ③ may induce CR effects.	152
	Specioside	*Stereospermum suaveolens*	*Caenorhabditis elegans*	① prolongs maximum lifespan under normal culture, heat, and oxidative stress conditions.	increases activities of antioxidant enzymes such as SOD and CAT.	153
Seco-iridoid	Gentiopicroside	*Gentiana rigescens*	K6001 yeast	① prolongs replicative and chronological lifespan.<br>② increases survival rate under oxidative stress condition.	① increases activities of CAT, SOD, and GSH-Px.<br>② induces autophagy, especially mitophagy.<br>③ requires *SOD1*, *SOD2*, *UTH1*, and *SKN7* genes.	154
	Oleuropein	*Oleaceae* family	*Caenorhabditis elegans*	① prolongs maximum lifespan under normal culture, heat, and oxidative stress conditions.<br>② decreases MDA levels.	① promotes DAF-16 nuclear localization and increases expression of target genes *via* IIS pathway.<br>② might partially depends on SKN-1.	155

Sesquiterpenoid	β-caryophyllene	edible plants	*Caenorhabditis elegans*	① prolongs maximum lifespan,<br>② decreases intestinal lipofuscin and ROS levels.<br>③ decreases food intake.<br>④ does not affect brood and body size.	① regulate expression of SIR-2.1, SKN-1 and their target genes.<br>② interacts with DAF-16 but might does not depend on it.<br>③ induces CR effects.	156
	SZMT01	Shenzhou honey peach fruit	K6001 yeast	① prolongs replicative lifespan.<br>② increases survival rate under oxidative stress.	at least partially needs genes *SOD1* and *SOD2*.	157
	α-santalol and β-santalol	*Santalum album*	*Caenorhabditis elegans*	① prolongs maximum lifespan.<br>② decreases ROS levels and apoptotic features induced by 6-OHDA.<br>③ reduces 6-OHDA and α-synuclein-induced Parkinson's disease associated pathologies.<br>④ increases physiological functions.	depends on mitochondrial electron transport chain, SKN-1 signaling pathway.	158
	Thapsigargin	*Thapsia garganica*	*Caenorhabditis elegans*	prolongs lifespan.	① reduces ER-mitochondria Ca^{2+} transfer that would activate AMPK and inhibit TORC1.<br>② increases autophagy activity.<br>③ at least partially depends on DAF-16.	159
	9 β-dihydroagarofuran-type Sesquiterpenes	*Celastrusv monospermus*	*Caenorhabditis elegans*	all prolong mean and maximum lifespan.	need further studies.	160

	Handelin	*Chrysanthemum indicum*	*Caenorhabditis elegans*	① prolongs healthspan and maximum lifespan.<br>② reduces ROS level and maintained the number and morphology of mitochondria.	need further studies.	161
Diterpenoid	Cryptotanshinone	*Salvia miltiorrhiza*	*Saccharomyces cerevisiae*	prolongs chronological lifespan in a dose- and the-time-of-addition-dependent manner without disruption of cell growth.	might be involved in regulation of Tor1, Sch9, Gcn2, and Sod2.	162
	Dehydroabietic acid	*Pinus densiflora, Pinus sylvestris, grandfir Abies grandis*	*Caenorhabditis elegans* and Dermal fibroblasts from adult	① prolongs maximum lifespan.<br>② decreases lipofuscin levels.<br>③ prevents collagen secretion in human dermal fibroblasts.	binds directly to SIRT1 protein independent of SIRT1 substrate NAD^+ levels, and activates SIRT1.	163
	Rebaudioside A	*Stevia rebaudiana*	*Caenorhabditis elegans*	① prolongs maximum lifespan under normal culture and oxidative stress condition.<br>② decreases ROS levels and neutral lipid accumulation.<br>③ does not affect food intake, brood and body size.	increases autophagy activity by inhibiting TOR and PI3K/Akt signaling pathways.	164
	Oridonin	*Rabdosia rubescens*	Yeast and naturally aged mice	① prolongs replicative lifespan of yeast.<br>② prolongs healthspan and mean lifespan of mice.	depends on AKT signaling pathway.	165
	Kahweol	Coffee	*Caenorhabditis elegans*	prolongs maximum lifespan.	depends on IIS and AKT signaling pathway.	166

Tricyclic diterpenoid	Triptolide	*Tripterygium wilfordii*	*Caenorhabditis elegans*	① prolongs maximum lifespan under normal culture, heat, UV irradiation, and oxidative stress conditions.<br>② decreases ROS levels.<br>③ does not affect brood size.	upregulates genes expression of *hsp-16.2* and *sod-3*.	167
	Carnosol	*Rosmarinus officinalis*	*Caenorhabditis elegans*	① prolongs maximum lifespan under normal culture and stress conditions.<br>② slows aging-related declines such as locomotor activity, age pigmentation, and neurodegenerative disease.<br>③ decreases ROS and MDA levels.<br>④ does not affect brood size and fat deposition.	① upregulates genes expression of *sod-3*, *sod-5*, *hsf-1*, *hsp-16.1*, and *hsp-16.2*.<br>② promotes DAF-16 nuclear localization.<br>③ depends on *hsf-1* rather than *daf-16*.	168
	Carnosic acid	*Rosmarinus officinalis*	*Caenorhabditis elegans*	① prolongs maximum lifespan under normal culture, heat, and oxidative stress conditions.<br>② increases locomotor activity.<br>③ reduces accumulation of age pigment, ④ delays Aβ-induced and polyQ-dependent paralysis.	① upregulates expression of SOD-3.<br>② might depend on MAPK and HSF-1 pathways.	169
Quassinoid triterpenoid	Glaucarubinone	*Simarouba glauca*	*Caenorhabditis elegans*	① prolongs maximum lifespan.<br>② promotes mitochondrial metabolism.	might induce mitohemesis effects.	170

				③ reduces body fat levels.		
Dammarane-type tetracyclic triterpenoid	Ginsenoside Rg1	*Panax ginseng*	D-gal-induced aging Sprague-Dawley rats	① increases cognitive ability.<br>② protects neural stem cells/progenitor cells (HSC/HPCs).<br>③ promotes neurogenesis.<br>④ decreases proinflammatory cytokines levels.<br>⑤ lengthens telomere。	① upregulates antioxidant enzymes and telomerase activities.<br>② downregulates mRNA expression of cellular senescence associated genes.	171
			D-gal-induced aging mice	① increases resistance of Sca-1^{+}<br>② HSC/HPCs. inhibits oxidative stress.<br>③ reduces DNA damage.<br>④ decreases MDA levels.	① downregulates Wnt/β-catenin signaling pathway activities.<br>② inhibits p16Ink4a-Rb and p53-p21Cip1/Waf1 pathways.	172
			Acetate-induced aging Sprague-Dawley rats	① ameliorates HSCs aging and aging associated inflammation response.<br>② reduces inflammation levels in blood plasma induced by acetate.	regulates aging associated p53-p21-Rb signaling network in hematopoietic system.	173
	Gentirigeoside B	*Gentiana rigescens*	K6001 yeast	① prolongs replicative and chronological lifespan.<br>② increases survival rate under oxidative stress.<br>③ decreases ROS and MDA levels.	inhibits TORC1/Sch9/Rim15/Msn signaling pathway and enhances autophagy.	174
Cucurbitane-type tetracyclic triterpenoid	Momordicoside G	*Momordica charantia*	K6001 yeast	① prolongs replicative lifespan.<br>② increases survival rate under oxidative stress.	① decreases *UTH1* and *SKN7* genes expression.	175

				③ decreases ROS levels.	② increases *SOD1* and *SOD2* genes expression.	
	Cucurbitacin B	Pedicellus melo	K6001 yeast	① prolongs replicative and chronological lifespan.<br>② decreases ROS and MDA levels.	① increases autophagy activity.<br>② regulates *SOD1*, *SOD2*, *UTH1*, and *SKN7* genes expression.	176
Cycloartane-type tetracyclic triterpenoid	Astragaloside IV	*Astragalus mongholicus*	*Caenorhabditis elegans*	① prolongs maximum lifespan under normal culture, oxidative, and heat stress conditions. ② increases locomotor ability.	① at least partially regulates IIS pathway activity.<br>② may induce hormesis effects.	177
Tetranortriterpenoids	Nomilin	*Citrus* fruits	*Caenorhabditis elegans,* D-gal-induced aging mice, doxorubicin-induced aging mice, and SAMP8 mice	① prolongs lifespan, healthspan, and toxin resistance of worm.<br>② prolongs healthspan and lifespan of D-galactose- and doxorubicin-induced<br>aging mice, and male SAMP8 mice.	① depends on IIS DAF-2/DAF-16 and nuclear hormone receptors NHR-8/DAF-12.<br>② directly binds with human pregnane X receptor (hPXR).	178
Oleanane-type pentacyclic triterpenoid	Oleanolic acid	*Olea europaea, Viscum album, Aralia chinensis*	*Caenorhabditis elegans*	① prolongs maximum lifespan under normal culture and oxidative stress conditions.<br>② decreases ROS levels.<br>③ does not affect pharyngeal pumping rate.	promotes DAF-16 nuclear localization and upregulates its target genes such as *sod-3*, *hsp-16.2*, and *ctl-1*.	179
	18α-Glycyrrhetinic acid	*Glycyrrhiza uralensis*	*Caenorhabditis elegans*, SHSY5Y human cell and Murine neurons.	① prolongs maximum lifespan of worms.<br>② decreases Aβ toxicity and aggregation in worms.	depends on SKN-1 and proteasome activation.	180

				③ decreases Aβ-induced neuronal death of SH-SY5Y neuroblastoma cells.		
Ursane-type pentacyclic triterpenoid	Ursolic acid	*Arctostaphylos uva-ursi*	naturally aged mice	delays aging or ameliorates aging.	① upregulates SIRT1 and SIRT6 activities.<br>② upregulates protein levels of PGC-1β and α-Klotho in hypothalamus.	181
			Drosophila melanogaster	① prolongs healthspan and maximum lifespan.<br>② affects gut microbiota of males.<br>③ does not reduce fecundity or gut integrity.	upregulates *srl* gene expression.	182
	Rotundic acid	*Ilex rotunda*	Yeast and naturally aged mice	① prolongs replicative lifespan of yeast.<br>② increased energy expenditure, BAT thermogenesis, and glucose metabolism in diet-induced obese mice.<br>③ prolongs healthspan and mean lifespan of mice.	inhibits leptin negative regulators protein tyrosine phosphatase 1B (PTP1B) noncompetitively.	183
Lupane-type pentacyclic triterpenoids	Betulinic acid	Widely in plants	*Drosophila melanogaster*	prolongs mean lifespan of both males and females.	depends on Sir2 and FoxO activation.	184

Supplementary Table 6 Summary of the anti-aging and/or lifespan-extending effects of Steroids

Type	Active ingredient	Source	Experimental model	Effect	Mechanism	References
Steroidal saponin	Nolinospiroside F	*Ophiopogon japonicus*	K6001 yeast	① prolongs replicative lifespan.<br>② increases survival under oxidative stress condition.<br>③ decreases MDA levels.	① increases antioxidant genes expression of *SOD1* and *SOD2*.<br>② increases activity of SIRT1.<br>③ inhibits expression of *UTH1*.	185
	Inokosterone	*Gentiana rigescens*	K6001 yeast	① prolongs replicative lifespan.<br>② decreases ROS and MDA levels.	① increases *SOD1* gene expression.<br>② increases mitophagy activity.	186
	Shatavarin IV	*Asparagus racemosus*	*Caenorhabditis elegans*	① prolongs maximum lifespan under normal culture and oxidative stress condition.<br>② decreases α-synuclein aggregation thus improving PD symptoms.<br>③ prevents ROS mediated oxidative damage.	① increases expression of stress responsive genes such as *sod-1*, *gst-4*, and *ctl-2*.<br>② upregulates PD associated genes *pdr-1*, *ubc-12*, and *pink-1*.<br>③ might utilize ubiquitin mediated proteasomal system.	187
C-21 steroidal glycoside	Otophylloside B	*Cynanchum otophyllum*	*Caenorhabditis elegans*	① prolongs maximum lifespan under normal culture and heat stress condition.<br>② decreases rate of locomotor activity decline.	① promotes DAF-16 nuclear localization.<br>② requires SIR-2.1 and CLK-1.<br>③ might reduce mitochondrial respiration.	188
			Caenorhabditis elegans	① prolongs maximum lifespan under normal culture and heat stress condition.<br>② protects against Aβ toxicity.	① activates DAF-16 and increases *sod-3* expression.<br>② upregulates expression of *hsf-1* and its target genes.	189
Cardiac glycoside	Convallatoxin	*Convallaria majalis*	*Caenorhabditis elegans*	① prolongs maximum lifespan under normal culture, heat, and oxidative stress conditions.<br>② decreases lipofuscin and ROS levels.	① promotes DAF-16 nuclear localization.<br>② upregulates stress responsive proteins such as SOD-3 and HSP-16.1.<br>③ depends on *sir-2.1* activity to protect against oxidative stress.	190

				③ increases pharyngeal pumping rate, brood size, and locomotor activity.		
Steroidal sapogenin	Diosgenin	*Dioscorea* genus	*Caenorhabditis elegans*	① prolongs maximum lifespan under normal culture, heat, glucose stress, and oxidative stress conditions.<br>② reduces ROS and lipid levels.	① promotes DAF-16 nuclear localization.<br>② regulate DAF-16 and SKN-1.<br>③ upregulate SOD-3 and GST-4 expression levels.	191
Steroidal lactone	Withaferin A	*Acnistus arborescens*	*Drosophila melanogaster*	① prolongs median and maximum lifespan of male.<br>② increases intestinal barrier permeability in older flies.	regulates genes involved in antioxidant defense expression *PrxV*, recognition of DNA damage *Gadd45*, repair of double-strand breaks *Ku80*, and heat shock proteins *Hsp68*, *Hsp83*.	192

Supplementary Table 7 Summary of the anti-aging and/or lifespan-extending effects of Alkaloids

**Type**	**Active ingredient**	**Source**	**Experimental model**	**Effect**	**Mechanism**	**References**
Methylxanthines	Caffeine	Tea and Coffee	*Caenorhabditis elegans*	① prolongs maximum lifespan.<br>② delays its larval development.<br>③ decreases body and brood size.	partially depends on IIS pathway while DAF-16 is not essential.	193
	Caffeine and Theophylline	Tea and Coffee	*Caenorhabditis elegans*	① prolongs maximum lifespan under normal culture and oxidative stress condition.<br>② high concentrations decreases body and brood size.	① does not depend on DAF-16/FOXO and SKN-1,<br>② may induce hormesis effects, and needs further research.	194
Yohimbine	Reserpine	*Rauwolfia serpentina*	*Caenorhabditis elegans*	① prolongs maximum lifespan.	regulates acetylcholine levels, needs further study.	195

				② alleviates age-dependent Aβ proteotoxicity acting independent of alteration of Aβ aggregation.		
				prolongs maximum lifespan.	① regulates of neurotransmitter especially acetylcholine release.<br>② requires both D2-type dopamine receptor *dop-3*, and exoribonuclease *eri-1*, which act in independent parallel pathways.	196
Steroidal alkaloid	Tomatidine	*Solanum* genus	*Caenorhabditis elegans*	① prolongs maximum lifespan.<br>② increases behaviors related to healthspan and muscle health.	increases mitophagy activity by activation of PINK1/DCT-1 pathway *via* SKN-1 activation.	197
Piperidine	Arecoline	*Areca catechu*	*Caenorhabditis elegans*	① prolongs maximum lifespan.<br>② decreases rate of locomotor activity decline with short-term treatment during early stage of aging.	① promotes synaptic exocytosis at neuromuscular junctions by acting on GAR-2/PLCβ pathway in motor neurons.<br>② DAF-16 might be partially required.	198
Nitrogen-containing pigment	4 betalains	*Caryophyllales* order	*Caenorhabditis elegans*	① prolongs mean lifespan up to 16.82%, 16.65%, 16.53%, and 12.93%, respectively.<br>② increases resistance to oxidative stress.	① upregulate DAF-16 and SKN-1.<br>② lead to overexpression of *HSPs* genes.	199
Pyridine	Trigonelline	*Trigonella foenum-graecum*	*Caenorhabditis elegans*	① prolongs maximum lifespan under normal culture, heat, pathogenic, and oxidative stress.<br>stress conditions.	① upregulates *daf-16*, *hsf-1*, and *aak-2*.<br>② upregulates expression of *daf-16* and *hsf-1* target genes such as *sod-3*, *gst-4*, *hsp-16.1*, and *hsp-12.6*.	200

				② delays development of neurodegenerative diseases such as AD, PD, and HD.		
Morphine	Morphine	*Papaver somniferum*	*Drosophila melanogaster*	prolongs mean lifespan in a dose-dependent manner.	needs further studies.	201
Vanilloid	Capsaicin	*Capsicum* genus	*Drosophila melanogaster*	prolongs mean lifespan in a sex specific way.	needs further studies.	202
pyrazine	Tetramethylpyrazine	*Ligusticum sinense 'Chuanxiong'*	Sprague–Dawley rats	① increases cell viability.<br>② delays bone marrow mesenchymal stem cell senescence.	① inhibits NF-κB signaling.<br>② decreases levels of pro-inflammatory factors.	203
Protoberberine	Berberine	*Coptis chinensis*	Doxorubicin-induced aging mice and naturally aged mice	① prolongs maximum lifespan of Doxorubicin-induced aging mice and naturally aged mice.<br>② increases healthspan, furdensity, as well as behavioral activity.	promotes cellular transition from G1 to S phase during early mitosis of cell cycle by downregulating expression of *p16*.	204
β-carbolines	Harmol	*Peganum harmala*	Worm, fly, and naturally aged mice	① prolongs lifespan of hermaphrodite worm and female fly.<br>② prolongs healthspan and induces mitohormesis of mice.	targets MAO-B and GABAAR.	205

Supplementary Table 8 Summary of the anti-aging and/or lifespan-extending effects of Others

**Active ingredient**	**Source**	**Experimental model**	**Effect**	**Mechanism**	**References**
Quinic acid	Widely in plants especially tea and	*Caenorhabditis elegans*	① prolongs maximum lifespan under normal culture, heat, and oxidative stress conditions.	① upregulates expression of *daf-16* and *sod-3*.	206

	coffee		② decreases ROS levels.	② depends on DAF-16.	
Allicin	*Allium sativum*	C57BL/6 mice	① decreases TBARS, ROS and carbonyl levels.<br>② ameliorates aging-induced cognitive dysfunction.	increases activities of GPx and levels of GSH-px *via* Nrf2/ARE signaling pathways.	207
Diallyl trisulfide	*Allium sativum*	*Caenorhabditis elegans*	prolongs mean lifespan even when treatment is started during young adulthood.	upregulates SKN-1 and its target gene *gst-4*.	208
S-allylcysteine, S-allylmercaptocysteine	*Allium sativum*	*Caenorhabditis elegans*	① both prolong mean lifespan and increase stress resistance.<br>② decrease ROS levels under oxidative and heat stress conditions.<br>③ do not affect body and brood size, but increase food intake.	upregulate SKN-1 activity and selectively activate its target gene.	209
Sulforaphane	*Brassica* genus	*Caenorhabditis elegans*	① prolongs maximum lifespan under normal culture and oxidative stress condition.<br>② increases locomotor activity and food intake.<br>③ decreases lipofuscin levels.<br>④ does not affect brood size.	① inhibits DAF-2 and its targets AGE-1, AKT-1/AKT-2.<br>② promotes DAF-16 nuclear localization.	210
Vanillic Acid	*Angelina sinensis*	*Caenorhabditis elegans*	① prolongs maximum lifespan under normal culture and heat stress condition.<br>② decreases the paralysis phenotype associated with β-amyloid and poly Q aggregation.	requires HSF-1 and is associated with increase of HSP-4 and hsp-16.2 expression.	211
Polyacetylene isofalcarintriol (IFT)	*Daucus carota*	*Caenorhabditis elegans* and naturally aged C57BL/6NRj mice	① prolongs healthspan and maximum lifespan of worm, reduces protein accumulation in worm models of neurodegeneration.<br>② improves glucose metabolism, increased exercise endurance, and attenuated parameters of frailty of mice.	interacts with α-subunit of mitochondrial ATP synthase to promote mitochondrial biogenesis.	212

Reference:

(1) Chandler-Brown, D.; Choi, H.; Park, S.; Ocampo, B. R.; Chen, S.; Le, A.; Sutphin, G. L.; Shamieh, L. S.; Smith, E. D.; Kaeberlein, M. Sorbitol Treatment Extends Lifespan and Induces the Osmotic Stress Response in Caenorhabditis Elegans. *Front. Genet.* **2015**, *6*, 316. https://doi.org/10.3389/fgene.2015.00316.

(2) Shintani, T.; Sakoguchi, H.; Yoshihara, A.; Izumori, K.; Sato, M. D-Allulose, a Stereoisomer of D-Fructose, Extends Caenorhabditis Elegans Lifespan through a Dietary Restriction Mechanism: A New Candidate Dietary Restriction Mimetic. *Biochem. Biophys. Res. Commun.* **2017**, *493* (4), 1528–1533. https://doi.org/10.1016/j.bbrc.2017.09.147.

(3) Li, X.-T.; Zhang, Y.-K.; Kuang, H.-X.; Jin, F.-X.; Liu, D.-W.; Gao, M.-B.; Liu, Z.; Xin, X.-J. Mitochondrial Protection and Anti-Aging Activity of Astragalus Polysaccharides and Their Potential Mechanism. *Int. J. Mol. Sci.* **2012**, *13* (2), 1747–1761. https://doi.org/10.3390/ijms13021747.

(4) Zhang, H.; Pan, N.; Xiong, S.; Zou, S.; Li, H.; Xiao, L.; Cao, Z.; Tunnacliffe, A.; Huang, Z. Inhibition of Polyglutamine-Mediated Proteotoxicity by Astragalus Membranaceus Polysaccharide through the DAF-16/FOXO Transcription Factor in Caenorhabditis Elegans. *Biochem. J.* **2012**, *441* (1), 417–424. https://doi.org/10.1042/BJ20110621.

(5) Song, J.; Chen, M.; Li, Z.; Zhang, J.; Hu, H.; Tong, X.; Dai, F. Astragalus Polysaccharide Extends Lifespan via Mitigating Endoplasmic Reticulum Stress in the Silkworm, Bombyx Mori. *Aging Dis.* **2019**, *10* (6), 1187. https://doi.org/10.14336/AD.2019.0515.

(6) Yang, F.; Xiu, M.; Yang, S.; Li, X.; Tuo, W.; Su, Y.; He, J.; Liu, Y. Extension of Drosophila Lifespan by Astragalus Polysaccharide through

a Mechanism Dependent on Antioxidant and Insulin/IGF-1 Signaling. *Evid. Based Complement. Alternat. Med.* **2021**, *2021*, 1–12. https://doi.org/10.1155/2021/6686748.

(7) Zhang, Y.; Lv, T.; Li, M.; Xue, T.; Liu, H.; Zhang, W.; Ding, X.; Zhuang, Z. Anti-Aging Effect of Polysaccharide from Bletilla Striate on Nematode Caenorhabditis Elegans. *Pharmacogn. Mag.* **2015**, *11* (43), 449–454. https://doi.org/10.4103/0973-1296.1604471.

(8) Feng, S.; Cheng, H.; Xu, Z.; Shen, S.; Yuan, M.; Liu, J.; Ding, C. Thermal Stress Resistance and Aging Effects of Panax Notoginseng Polysaccharides on Caenorhabditis Elegans. *Int. J. Biol. Macromol.* **2015**, *81*, 188–194. https://doi.org/10.1016/j.ijbiomac.2015.07.057.

(9) Hui, H.; Xin, A.; Cui, H.; Jin, H.; Yang, X.; Liu, H.; Qin, B. Anti-Aging Effects on Caenorhabditis Elegans of a Polysaccharide, O-Acetyl Glucomannan, from Roots of Lilium Davidii Var. Unicolor Cotton. *Int. J. Biol. Macromol.* **2020**, *155*, 846–852. https://doi.org/10.1016/j.ijbiomac.2020.03.206.

(10) Tang, Y.; Zhu, Z.-Y.; Liu, Y.; Sun, H.; Song, Q.-Y.; Zhang, Y. The Chemical Structure and Anti-Aging Bioactivity of an Acid Polysaccharide Obtained from Rose Buds. *Food Funct.* **2018**, *9* (4), 2300–2312. https://doi.org/10.1039/C8FO00206A.

(11) Tang, R.; Chen, X.; Dang, T.; Deng, Y.; Zou, Z.; Liu, Q.; Gong, G.; Song, S.; Ma, F.; Huang, L.; Wang, Z. Lycium Barbarum Polysaccharides Extend the Mean Lifespan of Drosophila Melanogaster. *Food Funct.* **2019**, *10* (7), 4231–4241. https://doi.org/10.1039/C8FO01751D.

(12) Si, Y.; Liu, X.; Ye, K.; Bonfini, A.; Hu, X. Y.; Buchon, N.; Gu, Z. Glucomannan Hydrolysate Promotes Gut Proliferative Homeostasis and Extends Life Span in Drosophila Melanogaster. *J. Gerontol. Ser. A* **2019**, *74* (10), 1549–1556. https://doi.org/10.1093/gerona/gly189.

(13) Doan, V. M.; Chen, C.; Lin, X.; Nguyen, V. P.; Nong, Z.; Li, W.; Chen, Q.; Ming, J.; Xie, Q.; Huang, R. Yulangsan Polysaccharide Improves

Redox Homeostasis and Immune Impairment in D-Galactose-Induced Mimetic Aging. *Food Funct.* **2015**, *6* (5), 1712–1718. https://doi.org/10.1039/C5FO00238A.

(14) Xu, Y.; Gao, Y.; Zhong, M.; Li, J.; Cao, H.; Huang, S.; Wei, R.; Zhang, K. Isolation, Characterization and Bioactivities of the Polysaccharides from Dicliptera Chinensis (L.) Juss. *Int. J. Biol. Macromol.* **2017**, *101*, 603–611. https://doi.org/10.1016/j.ijbiomac.2017.03.112.

(15) Shen, S.; Xu, Z.; Feng, S.; Wang, H.; Liu, J.; Zhou, L.; Yuan, M.; Huang, Y.; Ding, C. Structural Elucidation and Antiaging Activity of Polysaccharide from Paris Polyphylla Leaves. *Int. J. Biol. Macromol.* **2018**, *107*, 1613–1619. https://doi.org/10.1016/j.ijbiomac.2017.10.026.

(16) Zeng, H.; Liu, Z.; Wang, Y.; Yang, D.; Yang, R.; Qu, L. Studies on the Anti-Aging Activity of a Glycoprotein Isolated from Fupenzi (Rubus Chingii Hu.) and Its Regulation on Klotho Gene Expression in Mice Kidney. *Int. J. Biol. Macromol.* **2018**, *119*, 470–476. https://doi.org/10.1016/j.ijbiomac.2018.07.157.

(17) Cheng, X.; Yao, H.; Xiang, Y.; Chen, L.; Xiao, M.; Wang, Z.; Xiao, H.; Wang, L.; Wang, S.; Wang, Y. Effect of Angelica Polysaccharide on Brain Senescence of Nestin-GFP Mice Induced by D-Galactose. *Neurochem. Int.* **2019**, *122*, 149–156. https://doi.org/10.1016/j.neuint.2018.09.003.

(18) Hui, Y.; Jun-li, H.; Chuang, W. Anti-Oxidation and Anti-Aging Activity of Polysaccharide from Malus Micromalus Makino Fruit Wine. *Int. J. Biol. Macromol.* **2019**, *121*, 1203–1212. https://doi.org/10.1016/j.ijbiomac.2018.10.096.

(19) Jing, L.; Jiang, J.-R.; Liu, D.-M.; Sheng, J.-W.; Zhang, W.-F.; Li, Z.-J.; Wei, L.-Y. Structural Characterization and Antioxidant Activity of Polysaccharides from Athyrium Multidentatum (Doll.) Ching in D-Galactose-Induced Aging Mice via PI3K/AKT Pathway. *Molecules* **2019**,

24 (18), 3364. https://doi.org/10.3390/molecules24183364.

(20) Yang, S.; Xiu, M.; Li, X.; Shi, Y.; Wang, S.; Wan, S.; Han, S.; Yang, D.; Liu, Y.; He, J. The Antioxidant Effects of Hedysarum Polybotrys Polysaccharide in Extending Lifespan and Ameliorating Aging-Related Diseases in Drosophila Melanogaster. *Int. J. Biol. Macromol.* **2023**, *241*, 124609. https://doi.org/10.1016/j.ijbiomac.2023.124609.

(21) Liang, L.; Yue, Y.; Zhong, L.; Liang, Y.; Shi, R.; Luo, R.; Zhao, M.; Cao, X.; Yang, M.; Du, J.; Shen, X.; Wang, Y.; Shu, Z. Anti-Aging Activities of Rehmannia Glutinosa Libosch. Crude Polysaccharide in Caenorhabditis Elegans Based on Gut Microbiota and Metabonomic Analysis. *Int. J. Biol. Macromol.* **2023**, *253*, 127647. https://doi.org/10.1016/j.ijbiomac.2023.127647.

(22) Zarse, K.; Jabin, S.; Ristow, M. L-Theanine Extends Lifespan of Adult Caenorhabditis Elegans. *Eur. J. Nutr.* **2012**, *51* (6), 765–768. https://doi.org/10.1007/s00394-012-0341-5.

(23) Wang, Z.; Ma, X.; Li, J.; Cui, X. Peptides from Sesame Cake Extend Healthspan of Caenorhabditis Elegans via Upregulation of Skn-1 and Inhibition of Intracellular ROS Levels. *Exp. Gerontol.* **2016**, *82*, 139–149. https://doi.org/10.1016/j.exger.2016.07.001.

(24) Ma, X.; Cui, X.; Li, J.; Li, C.; Wang, Z. Peptides from Sesame Cake Reduce Oxidative Stress and Amyloid-β-Induced Toxicity by Upregulation of SKN-1 in a Transgenic Caenorhabditis Elegans Model of Alzheimer's Disease. *J. Funct. Foods* **2017**, *39*, 287–298. https://doi.org/10.1016/j.jff.2017.10.032.

(25) Li, F.; Ma, X.; Cui, X.; Li, J.; Wang, Z. Recombinant Buckwheat Glutaredoxin Intake Increases Lifespan and Stress Resistance via Hsf-1 Upregulation in Caenorhabditis Elegans. *Exp. Gerontol.* **2018**, *104*, 86–97. https://doi.org/10.1016/j.exger.2018.01.028.

(26) Li, J.; Cui, X.; Ma, X.; Wang, Z. rBTI Reduced β-Amyloid-Induced Toxicity by Promoting Autophagy-Lysosomal Degradation via DAF-

16 in Caenorhabditis Elegans. *Exp. Gerontol.* **2017**, *89*, 78–86. https://doi.org/10.1016/j.exger.2017.01.018.

(27) Wang, Q.; Huang, Y.; Qin, C.; Liang, M.; Mao, X.; Li, S.; Zou, Y.; Jia, W.; Li, H.; Ma, C. W.; Huang, Z. Bioactive Peptides from Angelica Sinensis Protein Hydrolyzate Delay Senescence in Caenorhabditis Elegans through Antioxidant Activities. *Oxid. Med. Cell. Longev.* **2016**, *2016*, 1–10. https://doi.org/10.1155/2016/8956981.

(28) Zhang, Z.; Zhao, Y.; Wang, X.; Lin, R.; Zhang, Y.; Ma, H.; Guo, Y.; Xu, L.; Zhao, B. The Novel Dipeptide Tyr-Ala (TA) Significantly Enhances the Lifespan and Healthspan of Caenorhabditis Elegans. *Food Funct.* **2016**, *7* (4), 1975–1984. https://doi.org/10.1039/C5FO01302J.

(29) Amakye, W. K.; Hou, C.; Xie, L.; Lin, X.; Gou, N.; Yuan, E.; Ren, J. Bioactive Anti-Aging Agents and the Identification of New Anti-Oxidant Soybean Peptides. *Food Biosci.* **2021**, *42*, 101194. https://doi.org/10.1016/j.fbio.2021.101194.

(30) Heidler, T.; Hartwig, K.; Daniel, H.; Wenzel, U. Caenorhabditis Elegans Lifespan Extension Caused by Treatment with an Orally Active ROS-Generator Is Dependent on DAF-16 and SIR-2.1. *Biogerontology* **2010**, *11* (2), 183–195. https://doi.org/10.1007/s10522-009-9239-x.

(31) Hunt, P. R.; Son, T. G.; Wilson, M. A.; Yu, Q.-S.; Wood, W. H.; Zhang, Y.; Becker, K. G.; Greig, N. H.; Mattson, M. P.; Camandola, S.; Wolkow, C. A. Extension of Lifespan in C. Elegans by Naphthoquinones That Act through Stress Hormesis Mechanisms. *PLoS ONE* **2011**, *6* (7), e21922. https://doi.org/10.1371/journal.pone.0021922.

(32) Zhao, X.; Lu, L.; Qi, Y.; Li, M.; Zhou, L. Emodin Extends Lifespan of Caenorhabditis Elegans through Insulin/IGF-1 Signaling Pathway Depending on DAF-16 and SIR-2.1. *Biosci. Biotechnol. Biochem.* **2017**, *81* (10), 1908–1916. https://doi.org/10.1080/09168451.2017.1365592.

(33) Pan, Y.; Liu, Y.; Fujii, R.; Farooq, U.; Cheng, L.; Matsuura, A.; Qi, J.; Xiang, L. Ehretiquinone from Onosma Bracteatum Wall Exhibits Antiaging Effect on Yeasts and Mammals through Antioxidative Stress and Autophagy Induction. *Oxid. Med. Cell. Longev.* **2021**, *2021*, 1–15. https://doi.org/10.1155/2021/5469849.

(34) Feng, Y.; Yu, Y.-H.; Wang, S.-T.; Ren, J.; Camer, D.; Hua, Y.-Z.; Zhang, Q.; Huang, J.; Xue, D.-L.; Zhang, X.-F.; Huang, X.-F.; Liu, Y. Chlorogenic Acid Protects D-Galactose-Induced Liver and Kidney Injury via Antioxidation and Anti-Inflammation Effects in Mice. *Pharm. Biol.* **2016**, *54* (6), 1027–1034. https://doi.org/10.3109/13880209.2015.1093510.

(35) Zheng, S.-Q.; Huang, X.-B.; Xing, T.-K.; Ding, A.-J.; Wu, G.-S.; Luo, H.-R. Chlorogenic Acid Extends the Lifespan of Caenorhabditis Elegans via Insulin/IGF-1 Signaling Pathway. *J. Gerontol. A. Biol. Sci. Med. Sci.* **2017**, glw105. https://doi.org/10.1093/gerona/glw105.

(36) Li, H.; Yu, X.; Li, C.; Ma, L.; Zhao, Z.; Guan, S.; Wang, L. Caffeic Acid Protects against Aβ Toxicity and Prolongs Lifespan in Caenorhabditis Elegans Models. *Food Funct.* **2021**, *12* (3), 1219–1231. https://doi.org/10.1039/D0FO02784G.

(37) Gutierrez-Zetina, S. M.; González-Manzano, S.; Ayuda-Durán, B.; Santos-Buelga, C.; González-Paramás, A. M. Caffeic and Dihydrocaffeic Acids Promote Longevity and Increase Stress Resistance in Caenorhabditis Elegans by Modulating Expression of Stress-Related Genes. *Molecules* **2021**, *26* (6), 1517. https://doi.org/10.3390/molecules26061517.

(38) Li, R.; Tao, M.; Wu, T.; Zhuo, Z.; Xu, T.; Pan, S.; Xu, X. A Promising Strategy for Investigating the Anti-Aging Effect of Natural Compounds: A Case Study of Caffeoylquinic Acids. *Food Funct.* **2021**, *12* (18), 8583–8593. https://doi.org/10.1039/D1FO01383A.

(39) Wang, F.; Liu, Q. D.; Wang, L.; Zhang, Q.; Hua, Z. T. The Molecular Mechanism of Rosmarinic Acid Extending the Lifespan of Caenorhabditis Elegans. *Appl. Mech. Mater.* **2011**, *140*, 469–472. https://doi.org/10.4028/www.scientific.net/AMM.140.469.

(40) Lin, C.; Xiao, J.; Xi, Y.; Zhang, X.; Zhong, Q.; Zheng, H.; Cao, Y.; Chen, Y. Rosmarinic Acid Improved Antioxidant Properties and Healthspan via the IIS and MAPK Pathways in Caenorhabditis Elegans. *BioFactors* **2019**, *45* (5), 774–787. https://doi.org/10.1002/biof.1536.

(41) Schlernitzauer, A.; Oiry, C.; Hamad, R.; Galas, S.; Cortade, F.; Chabi, B.; Casas, F.; Pessemesse, L.; Fouret, G.; Feillet-Coudray, C.; Cros, G.; Cabello, G.; Magous, R.; Wrutniak-Cabello, C. Chicoric Acid Is an Antioxidant Molecule That Stimulates AMP Kinase Pathway in L6 Myotubes and Extends Lifespan in Caenorhabditis Elegans. *PLoS ONE* **2013**, *8* (11), e78788. https://doi.org/10.1371/journal.pone.0078788.

(42) Peng, Y.; Sun, Q.; Gao, R.; Park, Y. AAK-2 and SKN-1 Are Involved in Chicoric-Acid-Induced Lifespan Extension in Caenorhabditis Elegans. *J. Agric. Food Chem.* **2019**, *67* (33), 9178–9186. https://doi.org/10.1021/acs.jafc.9b00705.

(43) Li, H.; Yu, X.; Meng, F.; Zhao, Z.; Guan, S.; Wang, L. Ferulic Acid Supplementation Increases Lifespan and Stress Resistance via Insulin/IGF-1 Signaling Pathway in c. Elegans. *Int. J. Mol. Sci.* **2021**, *22* (8), 4279. https://doi.org/10.3390/ijms22084279.

(44) Yi, X.; Jiang, S.; Qin, M.; Liu, K.; Cao, P.; Chen, S.; Deng, J.; Gao, C. Compounds from the Fruits of Mangrove Sonneratia Apetala: Isolation, Molecular Docking and Antiaging Effects Using a Caenorhabditis Elegans Model. *Bioorganic Chem.* **2020**, *99*, 103813. https://doi.org/10.1016/j.bioorg.2020.103813.

(45) Jiang, S.; Jiang, C.-P.; Cao, P.; Liu, Y.-H.; Gao, C.-H.; Yi, X.-X. Sonneradon a Extends Lifespan of Caenorhabditis Elegans by Modulating Mitochondrial and IIS Signaling Pathways. *Mar. Drugs* **2022**, *20* (1), 59. https://doi.org/10.3390/md20010059.

(46) Sayed, A. A. R. Ferulsinaic Acid Attenuation of Advanced Glycation End Products Extends the Lifespan of Caenorhabditis Elegans. *J. Pharm. Pharmacol.* **2011**, *63* (3), 423–428. https://doi.org/10.1111/j.2042-7158.2010.01222.x.

(47) Chamoli, M.; Rane, A.; Foulger, A.; Chinta, S. J.; Shahmirzadi, A. A.; Kumsta, C.; Nambiar, D. K.; Hall, D.; Holcom, A.; Angeli, S.; Schmidt, M.; Pitteri, S.; Hansen, M.; Lithgow, G. J.; Andersen, J. K. A Drug-like Molecule Engages Nuclear Hormone Receptor DAF-12/FXR to Regulate Mitophagy and Extend Lifespan. *Nat. Aging* **2023**. https://doi.org/10.1038/s43587-023-00524-9.

(48) Zuo, Y.; Peng, C.; Liang, Y.; Ma, K. Y.; Chan, H. Y. E.; Huang, Y.; Chen, Z.-Y. Sesamin Extends the Mean Lifespan of Fruit Flies. *Biogerontology* **2013**, *14* (2), 107–119. https://doi.org/10.1007/s10522-012-9413-4.

(49) Yaguchi, Y.; Komura, T.; Kashima, N.; Tamura, M.; Kage-Nakadai, E.; Saeki, S.; Terao, K.; Nishikawa, Y. Influence of Oral Supplementation with Sesamin on Longevity of Caenorhabditis Elegans and the Host Defense. *Eur. J. Nutr.* **2014**, *53* (8), 1659–1668. https://doi.org/10.1007/s00394-014-0671-6.

(50) Nakatani, Y.; Yaguchi, Y.; Komura, T.; Nakadai, M.; Terao, K.; Kage-Nakadai, E.; Nishikawa, Y. Sesamin Extends Lifespan through Pathways Related to Dietary Restriction in Caenorhabditis Elegans. *Eur. J. Nutr.* **2018**, *57* (3), 1137–1146. https://doi.org/10.1007/s00394-017-1396-0.

(51) Keowkase, R.; Shoomarom, N.; Bunargin, W.; Sitthithaworn, W.; Weerapreeyakul, N. Sesamin and Sesamolin Reduce Amyloid-β Toxicity in a Transgenic Caenorhabditis Elegans. *Biomed. Pharmacother.* **2018**, *107*, 656–664. https://doi.org/10.1016/j.biopha.2018.08.037.

(52) Su, S.; Wink, M. Natural Lignans from Arctium Lappa as Antiaging Agents in Caenorhabditis Elegans. *Phytochemistry* **2015**, *117*, 340–350. https://doi.org/10.1016/j.phytochem.2015.06.021.

(53) Ahn, J.-S.; Mahbub, N. U.; Kim, S.; Kim, H.-B.; Choi, J.-S.; Chung, H.-J.; Hong, S.-T. Nectandrin B Significantly Increases the Lifespan of Drosophila - Nectandrin B for Longevity. *AGING-US* **2023**, *15* (22), 12749–12762.

(54) Yu, J.; Gao, X.; Zhang, L.; Shi, H.; Yan, Y.; Han, Y.; Wu, C.; Liu, Y.; Fang, M.; Huang, C.; Fan, S. Magnolol Extends Lifespan and Improves Age-Related Neurodegeneration in *Caenorhabditis Elegans* via Increase of Stress Resistance. *Sci. Rep.* **2024**, *14* (1), 3158. https://doi.org/10.1038/s41598-024-53374-9.

(55) Havermann, S.; Rohrig, R.; Chovolou, Y.; Humpf, H.-U.; Wätjen, W. Molecular Effects of Baicalein in Hct116 Cells and Caenorhabditis Elegans: Activation of the Nrf2 Signaling Pathway and Prolongation of Lifespan. *J. Agric. Food Chem.* **2013**, *61* (9), 2158–2164. https://doi.org/10.1021/jf304553g.

(56) Sang, Y.; Zhang, F.; Wang, H.; Yao, J.; Chen, R.; Zhou, Z.; Yang, K.; Xie, Y.; Wan, T.; Ding, H. Apigenin Exhibits Protective Effects in a Mouse Model of D-Galactose-Induced Aging via Activating the Nrf2 Pathway. *Food Funct.* **2017**, *8* (6), 2331–2340. https://doi.org/10.1039/c7fo00037e.

(57) Guerrero-Rubio, M. A.; Hernández-García, S.; García-Carmona, F.; Gandía-Herrero, F. Flavonoids' Effects on Caenorhabditis Elegans' Longevity, Fat Accumulation, Stress Resistance and Gene Modulation Involve mTOR, SKN-1 and DAF-16. *Antioxidants* **2021**, *10* (3), 438. https://doi.org/10.3390/antiox10030438.

(58) Trivedi, S.; Pandey, R. 5′-Hydroxy-6, 7, 8, 3′, 4′-Pentamethoxyflavone Extends Longevity Mediated by DR-Induced Autophagy and Oxidative Stress Resistance in C. Elegans. *GeroScience* **2021**, *43* (2), 759–772. https://doi.org/10.1007/s11357-020-00229-6.

(59) Lu, M.; Tan, L.; Zhou, X.-G.; Yang, Z.-L.; Zhu, Q.; Chen, J.-N.; Luo, H.-R.; Wu, G.-S. Tectochrysin Increases Stress Resistance and Extends the Lifespan of Caenorhabditis Elegans via FOXO/DAF-16. *Biogerontology* **2020**, *21* (5), 669–682. https://doi.org/10.1007/s10522-020-09884-w.

(60) Yang, X.; Wang, H.; Li, T.; Chen, L.; Zheng, B.; Liu, R. H. Nobiletin Delays Aging and Enhances Stress Resistance of Caenorhabditis Elegans. *Int. J. Mol. Sci.* **2020**, *21* (1), 341. https://doi.org/10.3390/ijms21010341.

(61) Hong, Y.-X.; Wu, W.-Y.; Song, F.; Wu, C.; Li, G.-R.; Wang, Y. Cardiac Senescence Is Alleviated by the Natural Flavone Acacetin via Enhancing Mitophagy. *Aging* **2021**, *13* (12), 16381–16403. https://doi.org/10.18632/aging.203163.

(62) Liu, Y.; Zhou, Z.; Yin, L.; Zhu, M.; Wang, F.; Zhang, L.; Wang, H.; Zhou, Z.; Zhu, H.; Huang, C.; Fan, S. Tangeretin Promotes Lifespan Associated with Insulin/Insulin-like Growth Factor-1 Signaling Pathway and Heat Resistance in Caenorhabditis Elegans. *BioFactors* **2022**, *48* (2), 442–453. https://doi.org/10.1002/biof.1788.

(63) Xu, P.; Chen, Q.; Chen, X.; Qi, H.; Yang, Y.; Li, W.; Yang, X.; Gunawan, A.; Chen, S.; Zhang, H.; Shen, H.-M.; Huang, D.; Kennedy, B.; Xu, L.; Wu, Z. Morusin and Mulberrin Extend the Lifespans of Yeast and *C. Elegans* via Suppressing Nutrient-Sensing Pathways. *GeroScience* **2023**, *45* (2), 949–964. https://doi.org/10.1007/s11357-022-00693-2.

(64) Sugawara, T.; Sakamoto, K. Quercetin Enhances Motility in Aged and Heat-Stressed Caenorhabditis Elegans Nematodes by Modulating Both HSF-1 Activity, and Insulin-like and P38-MAPK Signalling. *PLOS ONE* **2020**, *15* (9), e0238528. https://doi.org/10.1371/journal.pone.0238528.

(65) Surco-Laos, F.; Cabello, J.; Gómez-Orte, E.; González-Manzano, S.; González-Paramás, A. M.; Santos-Buelga, C.; Dueñas, M. Effects of O-Methylated Metabolites of Quercetin on Oxidative Stress, Thermotolerance, Lifespan and Bioavailability on Caenorhabditis Elegans. *Food Funct.* **2011**, *2* (8), 445. https://doi.org/10.1039/c1fo10049a.

(66) Büchter, C.; Ackermann, D.; Havermann, S.; Honnen, S.; Chovolou, Y.; Fritz, G.; Kampkötter, A.; Wätjen, W. Myricetin-Mediated Lifespan

Extension in Caenorhabditis Elegans Is Modulated by DAF-16. *Int. J. Mol. Sci.* **2013**, *14* (6), 11895–11914. https://doi.org/10.3390/ijms140611895.

(67) Pandey, T.; Sammi, S. R.; Nooreen, Z.; Mishra, A.; Ahmad, A.; Bhatta, R. S.; Pandey, R. Anti-Ageing and Anti-Parkinsonian Effects of Natural Flavonol, Tambulin from Zanthoxyllum Aramatum Promotes Longevity in Caenorhabditis Elegans. *Exp. Gerontol.* **2019**, *120*, 50–61. https://doi.org/10.1016/j.exger.2019.02.016.

(68) Yousefzadeh, M. J.; Zhu, Y.; McGowan, S. J.; Angelini, L.; Fuhrmann-Stroissnigg, H.; Xu, M.; Ling, Y. Y.; Melos, K. I.; Pirtskhalava, T.; Inman, C. L.; McGuckian, C.; Wade, E. A.; Kato, J. I.; Grassi, D.; Wentworth, M.; Burd, C. E.; Arriaga, E. A.; Ladiges, W. L.; Tchkonia, T.; Kirkland, J. L.; Robbins, P. D.; Niedernhofer, L. J. Fisetin Is a Senotherapeutic That Extends Health and Lifespan. *EBioMedicine* **2018**, *36*, 18–28. https://doi.org/10.1016/j.ebiom.2018.09.015.

(69) Chattopadhyay, D.; Sen, S.; Chatterjee, R.; Roy, D.; James, J.; Thirumurugan, K. Context- and Dose-Dependent Modulatory Effects of Naringenin on Survival and Development of Drosophila Melanogaster. *Biogerontology* **2016**, *17* (2), 383–393. https://doi.org/10.1007/s10522-015-9624-6.

(70) Yeh, C.-H.; Shen, Z.-Q.; Wang, T.-W.; Kao, C.-H.; Teng, Y.-C.; Yeh, T.-K.; Lu, C.-K.; Tsai, T.-F. Hesperetin Promotes Longevity and Delays Aging via Activation of Cisd2 in Naturally Aged Mice. *J. Biomed. Sci.* **2022**, *29* (1), 53. https://doi.org/10.1186/s12929-022-00838-7.

(71) Filippopoulou, K.; Papaevgeniou, N.; Lefaki, M.; Paraskevopoulou, A.; Biedermann, D.; Křen, V.; Chondrogianni, N. 2,3-Dehydrosilybin A/B as a pro-Longevity and Anti-Aggregation Compound. *Free Radic. Biol. Med.* **2017**, *103*, 256–267.

https://doi.org/10.1016/j.freeradbiomed.2016.12.042.

(72) Fan, X.; Zeng, Y.; Fan, Z.; Cui, L.; Song, W.; Wu, Q.; Gao, Y.; Yang, D.; Mao, X.; Zeng, B.; Zhang, M.; Ni, Q.; Li, Y.; Wang, T.; Li, D.; Yang, M. Dihydromyricetin Promotes Longevity and Activates the Transcription Factors FOXO and AOP in Drosophila. *Aging-US* **2021**, *13* (1), 460–476. https://doi.org/10.18632/aging.202156.

(73) Liu, X.-L.; Zhao, Y.-C.; Zhu, H.-Y.; Wu, M.; Zheng, Y.-N.; Yang, M.; Cheng, Z.-Q.; Ding, C.-B.; Liu, W.-C. Taxifolin Retards the D-Galactose-Induced Aging Process through Inhibiting Nrf2-Mediated Oxidative Stress and Regulating the Gut Microbiota in Mice. *Food Funct.* **2021**, *12* (23), 12142–12158. https://doi.org/10.1039/D1FO01349A.

(74) Lee, E. B.; Ahn, D.; Kim, B. J.; Lee, S. Y.; Seo, H. W.; Cha, Y.-S.; Jeon, H.; Eun, J. S.; Cha, D. S.; Kim, D. K. Genistein from Vigna Angularis Extends Lifespan in Caenorhabditis Elegans. *Biomol. Ther.* **2015**, *23* (1), 77–83. https://doi.org/10.4062/biomolther.2014.075.

(75) Hou, Q.; Huang, J.; Zhao, L.; Pan, X.; Liao, C.; Jiang, Q.; Lei, J.; Guo, F.; Cui, J.; Guo, Y.; Zhang, B. Dietary Genistein Increases Microbiota-Derived Short Chain Fatty Acid Levels, Modulates Homeostasis of the Aging Gut, and Extends Healthspan and Lifespan. *Pharmacol. Res.* **2023**, *188*, 106676. https://doi.org/10.1016/j.phrs.2023.106676.

(76) Lu, L.; Zhao, X.; Zhang, J.; Li, M.; Qi, Y.; Zhou, L. Calycosin Promotes Lifespan in Caenorhabditis Elegans through Insulin Signaling Pathway via Daf-16, Age-1 and Daf-2. *J. Biosci. Bioeng.* **2017**, *124* (1), 1–7. https://doi.org/10.1016/j.jbiosc.2017.02.021.

(77) Piegholdt, S.; Rimbach, G.; Wagner, A. E. The Phytoestrogen Prunetin Affects Body Composition and Improves Fitness and Lifespan in Male Drosophila Melanogaster. *FASEB J.* **2016**, *30* (2), 948–958. https://doi.org/10.1096/fj.15-282061.

(78) Wang, T.-H.; Tseng, W.-C.; Leu, Y.-L.; Chen, C.-Y.; Lee, W.-C.; Chi, Y.-C.; Cheng, S.-F.; Lai, C.-Y.; Kuo, C.-H.; Yang, S.-L.; Yang, S.-

H.; Shen, J.-J.; Feng, C.-H.; Wu, C.-C.; Hwang, T.-L.; Wang, C.-J.; Wang, S.-H.; Chen, C.-C. The Flavonoid Corylin Exhibits Lifespan Extension Properties in Mouse. *Nat. Commun.* **2022**, *13* (1), 1238. https://doi.org/10.1038/s41467-022-28908-2.

(79) Kang, A.-W.; Sun, C.; Li, H.-T.; Zhong, K.; Zeng, X.-H.; Gu, Z.-F.; Li, B.-Q.; Zhang, X.-N.; Gao, J.-L.; Chen, T.-X. Puerarin Extends the Lifespan of *Drosophila Melanogaster* by Activating Autophagy. *Food Funct.* **2023**, *14* (4), 2149–2161. https://doi.org/10.1039/D2FO02800J.

(80) Rathor, L.; Pandey, R. Age-Induced Diminution of Free Radicals by Boeravinone B in Caenorhabditis Elegans. *Exp. Gerontol.* **2018**, *111*, 94–106. https://doi.org/10.1016/j.exger.2018.07.005.

(81) Wongchum, N.; Dechakhamphu, A. Xanthohumol Prolongs Lifespan and Decreases Stress-Induced Mortality in Drosophila Melanogaster. *Comp. Biochem. Physiol. Part C Toxicol. Pharmacol.* **2021**, *244*, 108994. https://doi.org/10.1016/j.cbpc.2021.108994.

(82) Carmona-Gutierrez, D.; Zimmermann, A.; Kainz, K.; Pietrocola, F.; Chen, G.; Maglioni, S.; Schiavi, A.; Nah, J.; Mertel, S.; Beuschel, C. B.; Castoldi, F.; Sica, V.; Trausinger, G.; Raml, R.; Sommer, C.; Schroeder, S.; Hofer, S. J.; Bauer, M. A.; Pendl, T.; Tadic, J.; Dammbrueck, C.; Hu, Z.; Ruckenstuhl, C.; Eisenberg, T.; Durand, S.; Bossut, N.; Aprahamian, F.; Abdellatif, M.; Sedej, S.; Enot, D. P.; Wolinski, H.; Dengjel, J.; Kepp, O.; Magnes, C.; Sinner, F.; Pieber, T. R.; Sadoshima, J.; Ventura, N.; Sigrist, S. J.; Kroemer, G.; Madeo, F. The Flavonoid 4,4′-Dimethoxychalcone Promotes Autophagy-Dependent Longevity across Species. *Nat. Commun.* **2019**, *10* (1), 651. https://doi.org/10.1038/s41467-019-08555-w.

(83) Kim, S.; Yoon, H.; Park, S.-K. Butein Increases Resistance to Oxidative Stress and Lifespan with Positive Effects on the Risk of Age-Related Diseases in Caenorhabditis Elegans. *ANTIOXIDANTS* **2024**, *13* (2), 155. https://doi.org/10.3390/antiox13020155.

(84) Kim, Y. S.; Han, Y. T.; Jeon, H.; Cha, D. S. Antiageing Properties of Damaurone D in Caenorhabditis Elegans. *J. Pharm. Pharmacol.* **2018**, *70* (10), 1423–1429. https://doi.org/10.1111/jphp.12979.

(85) Lim, H. J.; Han, Y. T.; Ahn, J.; Jeon, Y.; Jeon, H.; Cha, D. S. Longevity Effects of Hispidol in Caenorhabditis Elegans. *BioFactors* **2020**, *46* (6), 1041–1048. https://doi.org/10.1002/biof.1695.

(86) Zhao, J.; Zhu, A.; Sun, Y.; Zhang, W.; Zhang, T.; Gao, Y.; Shan, D.; Wang, S.; Li, G.; Zeng, K.; Wang, Q. Beneficial Effects of Sappanone A on Lifespan and Thermotolerance in Caenorhabditis Elegans. *Eur. J. Pharmacol.* **2020**, *888*, 173558. https://doi.org/10.1016/j.ejphar.2020.173558.

(87) Lee, E. B.; Xing, M. M.; Kim, D. K. Lifespan-Extending and Stress Resistance Properties of Brazilin from Caesalpinia Sappan in Caenorhabditis Elegans. *Arch. Pharm. Res.* **2017**, *40* (7), 825–835. https://doi.org/10.1007/s12272-017-0920-3.

(88) Kumar, J.; Park, K.-C.; Awasthi, A.; Prasad, B. Silymarin Extends Lifespan and Reduces Proteotoxicity in C. Elegans Alzheimer's Model. *CNS Neurol. Disord. Drug Targets* **2015**, *14* (2), 295–302. https://doi.org/10.2174/1871527314666150116110212.

(89) Wen, H.; Gao, X.; Qin, J. Probing the Anti-Aging Role of Polydatin in Caenorhabditis Elegans on a Chip. *Integr Biol* **2014**, *6* (1), 35–43. https://doi.org/10.1039/C3IB40191J.

(90) Wang, H.-L.; Gao, J.-P.; Han, Y.-L.; Xu, X.; Wu, R.; Gao, Y.; Cui, X.-H. Comparative Studies of Polydatin and Resveratrol on Mutual Transformation and Antioxidative Effect in Vivo. *Phytomedicine* **2015**, *22* (5), 553–559. https://doi.org/10.1016/j.phymed.2015.03.014.

(91) Chattopadhyay, D.; Chitnis, A.; Talekar, A.; Mulay, P.; Makkar, M.; James, J.; Thirumurugan, K. Hormetic Efficacy of Rutin to Promote Longevity in Drosophila Melanogaster. *Biogerontology* **2017**, *18* (3), 397–411. https://doi.org/10.1007/s10522-017-9700-1.

(92) Cordeiro, L. M.; Machado, M. L.; da Silva, A. F.; Obetine Baptista, F. B.; da Silveira, T. L.; Soares, F. A. A.; Arantes, L. P. Rutin Protects Huntington's Disease through the Insulin/IGF1 (IIS) Signaling Pathway and Autophagy Activity: Study in Caenorhabditis Elegans Model. *Food Chem. Toxicol.* **2020**, *141*, 111323. https://doi.org/10.1016/j.fct.2020.111323.

(93) Cai, W.-J.; Huang, J.-H.; Zhang, S.-Q.; Wu, B.; Kapahi, P.; Zhang, X.-M.; Shen, Z.-Y. Icariin and Its Derivative Icariside II Extend Healthspan via Insulin/IGF-1 Pathway in C. Elegans. *PLoS ONE* **2011**, *6* (12), e28835. https://doi.org/10.1371/journal.pone.0028835.

(94) Zhang, S.-Q.; Cai, W.-J.; Huang, J.-H.; Wu, B.; Xia, S.-J.; Chen, X.-L.; Zhang, X.-M.; Shen, Z.-Y. Icariin, a Natural Flavonol Glycoside, Extends Healthspan in Mice. *Exp. Gerontol.* **2015**, *69*, 226–235. https://doi.org/10.1016/j.exger.2015.06.020.

(95) Xiang, L.; Sun, K.; Lu, J.; Weng, Y.; Taoka, A.; Sakagami, Y.; Qi, J. Anti-Aging Effects of Phloridzin, an Apple Polyphenol, on Yeast via the SOD and Sir2 Genes. *Biosci. Biotechnol. Biochem.* **2011**, *75* (5), 854–858. https://doi.org/10.1271/bbb.100774.

(96) Sun, K.; Xiang, L.; Ishihara, S.; Matsuura, A.; Sakagami, Y.; Qi, J. Anti-Aging Effects of Hesperidin on Saccharomyces Cerevisiae Viainhibition of Reactive Oxygen Species and Uth1 Gene Expression. *Biosci. Biotechnol. Biochem.* **2012**, *76* (4), 640–645. https://doi.org/10.1271/bbb.110535.

(97) Kumar, S.; Akhila, P. V.; Suchiang, K. Hesperidin Ameliorates Amyloid-β Toxicity and Enhances Oxidative Stress Resistance and Lifespan of Caenorhabditis Elegans through Acr-16 Mediated Activation of the Autophagy Pathway. *Free Radic. Biol. Med.* **2023**, *209*, 366–380. https://doi.org/10.1016/j.freeradbiomed.2023.10.408.

(98) Lin, Y.; Sun, Y.; Weng, Y.; Matsuura, A.; Xiang, L.; Qi, J. Parishin from Gastrodia Elata Extends the Lifespan of Yeast via Regulation of Sir2/Uth1/TOR Signaling Pathway. *Oxid. Med. Cell. Longev.* **2016**, *2016*, 1–11. https://doi.org/10.1155/2016/4074690.

(99) Farooq, U.; Pan, Y.; Lin, Y.; Wang, Y.; Osada, H.; Xiang, L.; Qi, J. Structure Characterization and Action Mechanism of an Antiaging New Compound from Gastrodia Elata Blume. *Oxid. Med. Cell. Longev.* **2019**, *2019*, 1–10. https://doi.org/10.1155/2019/5459862.

(100) Guo, C.; Zhang, H.; Guan, X.; Zhou, Z. The Anti-Aging Potential of Neohesperidin and Its Synergistic Effects with Other Citrus Flavonoids in Extending Chronological Lifespan of Saccharomyces Cerevisiae BY4742. *Molecules* **2019**, *24* (22), 4093. https://doi.org/10.3390/molecules24224093.

(101) Chen, Y.; Onken, B.; Chen, H.; Xiao, S.; Liu, X.; Driscoll, M.; Cao, Y.; Huang, Q. Mechanism of Longevity Extension of Caenorhabditis Elegans Induced by Pentagalloyl Glucose Isolated from Eucalyptus Leaves. *J. Agric. Food Chem.* **2014**, *62* (15), 3422–3431. https://doi.org/10.1021/jf500210p.

(102) Asthana, J.; Yadav, D.; Pant, A.; Yadav, A. K.; Gupta, M. M.; Pandey, R. Acacetin 7-O-α-l-Rhamnopyranosyl (1–2) β-D-Xylopyranoside Elicits Life-Span Extension and Stress Resistance in Caenorhabditis Elegans. *J. Gerontol. A. Biol. Sci. Med. Sci.* **2016**, *71* (9), 1160–1168. https://doi.org/10.1093/gerona/glv173.

(103) Büchter, C.; Zhao, L.; Havermann, S.; Honnen, S.; Fritz, G.; Proksch, P.; Wätjen, W. TSG (2,3,5,4′-Tetrahydroxystilbene-2-O-*β*-D-Glucoside) from the Chinese Herb Polygonum Multiflorum Increases Life Span and Stress Resistance of Caenorhabditis Elegans. *Oxid. Med. Cell. Longev.* **2015**, *2015*, 1–12. https://doi.org/10.1155/2015/124357.

(104) Lee, E. B.; Kim, J. H.; Cha, Y.-S.; Kim, M.; Song, S. B.; Cha, D. S.; Jeon, H.; Eun, J. S.; Han, S.; Kim, D. K. Lifespan Extending and Stress Resistant Properties of Vitexin from Vigna Angularis in Caenorhabditis Elegans. *Biomol. Ther.* **2015**, *23* (6), 582–589. https://doi.org/10.4062/biomolther.2015.128.

(105) Tao, M.; Li, R.; Zhang, Z.; Wu, T.; Xu, T.; Zogona, D.; Huang, Y.; Pan, S.; Xu, X. Vitexin and Isovitexin Act through Inhibition of Insulin Receptor to Promote Longevity and Fitness in Caenorhabditis Elegans. *Mol. Nutr. Food Res.* **2022**, *66* (17), 2100845. https://doi.org/10.1002/mnfr.202100845.

(106) Pant, A.; Asthana, J.; Yadav, A. K.; Rathor, L.; Srivastava, S.; Gupta, M. M.; Pandey, R. Verminoside Mediates Life Span Extension and Alleviates Stress in Caenorhabditis Elegans. *Free Radic. Res.* **2015**, *49* (11), 1384–1392. https://doi.org/10.3109/10715762.2015.1075017.

(107) Wang, X.; Zhang, J.; Lu, L.; Zhou, L. The Longevity Effect of Echinacoside in Caenorhabditis Elegans Mediated through Daf-16. *Biosci. Biotechnol. Biochem.* **2015**, *79* (10), 1676–1683. https://doi.org/10.1080/09168451.2015.1046364.

(108) Zhou, L.; Fu, X.; Jiang, L.; Wang, L.; Bai, S.; Jiao, Y.; Xing, S.; Li, W.; Ma, J. Arbutin Increases Caenorhabditis Elegans Longevity and Stress Resistance. *PeerJ* **2017**, *5*, e4170. https://doi.org/10.7717/peerj.4170.

(109) Zhu, Q.; Qu, Y.; Zhou, X.-G.; Chen, J.-N.; Luo, H.-R.; Wu, G.-S. A Dihydroflavonoid Naringin Extends the Lifespan of C. Elegans and Delays the Progression of Aging-Related Diseases in PD/AD Models via DAF-16. *Oxid. Med. Cell. Longev.* **2020**, *2020*, 1–14. https://doi.org/10.1155/2020/6069354.

(110) Du, X.; Wang, K.; Sang, X.; Meng, X.; Xie, J.; Wang, T.; Liu, X.; Huang, Q.; Zhang, N.; Wang, H. Naringin Ameliorates H2O2-Induced Oxidative Damage in Cells and Prolongs the Lifespan of Female Drosophila Melanogaster via the Insulin Signaling Pathway. *FOOD Sci. Hum. WELLNESS* **2024**, *13* (3), 1231–1245. https://doi.org/10.26599/FSHW.2022.9250103.

(111) Lu, M.; Tan, L.; Zhou, X.-G.; Yang, Z.-L.; Zhu, Q.; Chen, J.-N.; Luo, H.-R.; Wu, G.-S. Secoisolariciresinol Diglucoside Delays the Progression of Aging-Related Diseases and Extends the Lifespan of Caenorhabditis Elegans via DAF-16 and HSF-1. *Oxid. Med. Cell.*

Longev. **2020**, *2020*, 1–13. https://doi.org/10.1155/2020/1293935.

(112) Li, N.; Li, X.; Shi, Y.-L.; Gao, J.-M.; He, Y.-Q.; Li, F.; Shi, J.-S.; Gong, Q.-H. Trilobatin, a Component from Lithocarpus Polystachyrus Rehd., Increases Longevity in C. Elegans through Activating SKN1/SIRT3/DAF16 Signaling Pathway. *Front. Pharmacol.* **2021**, *12*, 655045. https://doi.org/10.3389/fphar.2021.655045.

(113) Qu, Y.; Shi, L.; Liu, Y.; Huang, L.; Luo, H.-R.; Wu, G.-S. Orientin Prolongs the Longevity of Caenorhabditis Elegans and Postpones the Development of Neurodegenerative Diseases via Nutrition Sensing and Cellular Protective Pathways. *Oxid. Med. Cell. Longev.* **2022**, *2022*, 8878923. https://doi.org/10.1155/2022/8878923.

(114) Chatzigeorgiou, S.; Thai, Q. D.; Tchoumtchoua, J.; Tallas, K.; Tsakiri, E. N.; Papassideri, I.; Halabalaki, M.; Skaltsounis, A.-L.; Trougakos, I. P. Isolation of Natural Products with Anti-Ageing Activity from the Fruits of Platanus Orientalis. *Phytomedicine* **2017**, *33*, 53–61. https://doi.org/10.1016/j.phymed.2017.07.009.

(115) Xiong, L.; Mao, S.; Lu, B.; Yang, J.; Zhou, F.; Hu, Y.; Jiang, Y.; Shen, C.; Zhao, Y. Osmanthus Fragrans Flower Extract and Acteoside Protect against D-Galactose-Induced Aging in an ICR Mouse Model. *J. Med. Food* **2016**, *19* (1), 54–61. https://doi.org/10.1089/jmf.2015.3462.

(116) Zhou, X.; Yang, Q.; Xie, Y.; Sun, J.; Hu, J.; Qiu, P.; Cao, W.; Wang, S. Tetrahydroxystilbene Glucoside Extends Mouse Life Span via Upregulating Neural Klotho and Downregulating Neural Insulin or Insulin-like Growth Factor 1. *Neurobiol. Aging* **2015**, *36* (3), 1462–1470. https://doi.org/10.1016/j.neurobiolaging.2014.11.002.

(117) Elkhedir, A. E.; Iqbal, A.; Zogona, D.; Mohammed, H. H.; Murtaza, A.; Xu, X. Apigenin Glycosides from Green Pepper Enhance Longevity

and Stress Resistance in Caenorhabditis Elegans. *Nutr. Res.* **2022**, *102*, 23–34. https://doi.org/10.1016/j.nutres.2022.02.003.

(118) Liu, Y.; Shen, L.; Matsuura, A.; Xiang, L.; Qi, J. Isoquercitrin from Apocynum Venetum L. Exerts Antiaging Effects on Yeasts via Stress Resistance Improvement and Mitophagy Induction through the Sch9/Rim15/Msn Signaling Pathway. *ANTIOXIDANTS* **2023**, *12* (11), 1939. https://doi.org/10.3390/antiox12111939.

(119) Liang, L.; Zheng, T.; Fan, X.; Gao, Y.; Chen, X.; Wang, B.; Liu, Y.; Zhang, Y. Rosavin Extends Lifespan via the Insulin/IGF-1 Signaling Pathway in Caenorhabditis Elegans. *Naunyn. Schmiedebergs Arch. Pharmacol.* **2024**. https://doi.org/10.1007/s00210-024-02952-9.

(120) Xiao, Y.; Zhang, Y.; Li, L.; Jiang, N.; Yu, C.; Li, S.; Zhu, X.; Liu, F.; Liu, Y. Cynaroside Extends Lifespan and Improves the Neurondegeneration Diseases via Insulin/IGF-1 Signaling Pathway in Caenorhabditis Elegans. *Arch. Gerontol. Geriatr.* **2024**, *122*, 105377. https://doi.org/10.1016/j.archger.2024.105377.

(121) Lee, K.-S.; Lee, B.-S.; Semnani, S.; Avanesian, A.; Um, C.-Y.; Jeon, H.-J.; Seong, K.-M.; Yu, K.; Min, K.-J.; Jafari, M. Curcumin Extends Life Span, Improves Health Span, and Modulates the Expression of Age-Associated Aging Genes in Drosophila Melanogaster. *Rejuvenation Res.* **2010**, *13* (5), 561–570. https://doi.org/10.1089/rej.2010.1031.

(122) Liao, V. H.-C.; Yu, C.-W.; Chu, Y.-J.; Li, W.-H.; Hsieh, Y.-C.; Wang, T.-T. Curcumin-Mediated Lifespan Extension in Caenorhabditis Elegans. *Mech. Ageing Dev.* **2011**, *132* (10), 480–487. https://doi.org/10.1016/j.mad.2011.07.008.

(123) Shen, L.-R.; Xiao, F.; Yuan, P.; Chen, Y.; Gao, Q.-K.; Parnell, L. D.; Meydani, M.; Ordovas, J. M.; Li, D.; Lai, C.-Q. Curcumin-Supplemented Diets Increase Superoxide Dismutase Activity and Mean Lifespan in Drosophila. *AGE* **2013**, *35* (4), 1133–1142. https://doi.org/10.1007/s11357-012-9438-2.

(124) Akinyemi, A. J.; Oboh, G.; Ogunsuyi, O.; Abolaji, A. O.; Udofia, A. Curcumin-Supplemented Diets Improve Antioxidant Enzymes and Alter Acetylcholinesterase Genes Expression Level in Drosophila Melanogaster Model. *Metab. Brain Dis.* **2018**, *33* (2), 369–375. https://doi.org/10.1007/s11011-017-0100-7.

(125) Stępień, K.; Wojdyła, D.; Nowak, K.; Mołoń, M. Impact of Curcumin on Replicative and Chronological Aging in the Saccharomyces Cerevisiae Yeast. *Biogerontology* **2020**, *21* (1), 109–123. https://doi.org/10.1007/s10522-019-09846-x.

(126) Morselli, E.; Maiuri, M. C.; Markaki, M.; Megalou, E.; Pasparaki, A.; Palikaras, K.; Criollo, A.; Galluzzi, L.; Malik, S. A.; Vitale, I.; Michaud, M.; Madeo, F.; Tavernarakis, N.; Kroemer, G. Caloric Restriction and Resveratrol Promote Longevity through the Sirtuin-1-Dependent Induction of Autophagy. *Cell Death Dis.* **2010**, *1* (1), e10–e10. https://doi.org/10.1038/cddis.2009.8.

(127) Yu, X.; Li, G. Effects of Resveratrol on Longevity, Cognitive Ability and Aging-Related Histological Markers in the Annual Fish Nothobranchius Guentheri. *Exp. Gerontol.* **2012**, *47* (12), 940–949. https://doi.org/10.1016/j.exger.2012.08.009.

(128) Rascón, B.; Hubbard, B. P.; Sinclair, D. A.; Amdam, G. V. The Lifespan Extension Effects of Resveratrol Are Conserved in the Honey Bee and May Be Driven by a Mechanism Related to Caloric Restriction. *Aging-US* **2012**, *4* (7), 499–508. https://doi.org/10.18632/aging.100474.

(129) Lee, J.; Kwon, G.; Park, J.; Kim, J.-K.; Lim, Y.-H. Brief Communication: SIR-2.1-Dependent Lifespan Extension of Caenorhabditis Elegans by Oxyresveratrol and Resveratrol. *Exp. Biol. Med.* **2016**, *241* (16), 1757–1763. https://doi.org/10.1177/1535370216650054.

(130) Yoon, D. S.; Cha, D. S.; Choi, Y.; Lee, J. W.; Lee, M.-H. MPK-1/ERK Is Required for the Full Activity of Resveratrol in Extended Lifespan and Reproduction. *Aging Cell* **2019**, *18* (1), e12867. https://doi.org/10.1111/acel.12867.

(131) Shen, P.; Yue, Y.; Sun, Q.; Kasireddy, N.; Kim, K.-H.; Park, Y. Piceatannol Extends the Lifespan of Caenorhabditis Elegans via DAF-16.

BioFactors **2017**, *43* (3), 379–387. https://doi.org/10.1002/biof.1346.

(132) Saul, N.; Pietsch, K.; Menzel, R.; Stürzenbaum, S. R.; Steinberg, C. E. W. The Longevity Effect of Tannic Acid in Caenorhabditis Elegans: Disposable Soma Meets Hormesis. 10.

(133) Niu, Y.; Na, L.; Feng, R.; Gong, L.; Zhao, Y.; Li, Q.; Li, Y.; Sun, C. The Phytochemical, EGCG, Extends Lifespan by Reducing Liver and Kidney Function Damage and Improving Age-Associated Inflammation and Oxidative Stress in Healthy Rats. *Aging Cell* **2013**, *12* (6), 1041–1049. https://doi.org/10.1111/acel.12133.

(134) Yuan, H.; Li, Y.; Ling, F.; Guan, Y.; Zhang, D.; Zhu, Q.; Liu, J.; Wu, Y.; Niu, Y. The Phytochemical Epigallocatechin Gallate Prolongs the Lifespan by Improving Lipid Metabolism, Reducing Inflammation and Oxidative Stress in High-Fat Diet-Fed Obese Rats. *Aging Cell* **2020**, *19* (9), e13199. https://doi.org/10.1111/acel.13199.

(135) Xiong, L.-G.; Chen, Y.-J.; Tong, J.-W.; Gong, Y.-S.; Huang, J.-A.; Liu, Z.-H. Epigallocatechin-3-Gallate Promotes Healthy Lifespan through Mitohormesis during Early-to-Mid Adulthood in Caenorhabditis Elegans. *Redox Biol.* **2018**, *14*, 305–315. https://doi.org/10.1016/j.redox.2017.09.019.

(136) Tian, J.; Geiss, C.; Zarse, K.; Madreiter-Sokolowski, C. T.; Ristow, M. Green Tea Catechins EGCG and ECG Enhance the Fitness and Lifespan of Caenorhabditis Elegans by Complex I Inhibition. *Aging-US* **2021**, *13* (19), 22629–22648. https://doi.org/10.18632/aging.203597.

(137) Kharat, P.; Sarkar, P.; Mouliganesh, S.; Tiwary, V.; Priya, V. B. R.; Sree, N. Y.; Annapoorna, H. V.; Saikia, D. K.; Mahanta, K.; Thirumurugan, K. Ellagic Acid Prolongs the Lifespan of Drosophila Melanogaster. *GeroScience* **2020**, *42* (1), 271–285. https://doi.org/10.1007/s11357-019-00135-6.

(138) Kim, Y. S.; Seo, H. W.; Lee, M.-H.; Kim, D. K.; Jeon, H.; Cha, D. S. Protocatechuic Acid Extends Lifespan and Increases Stress Resistance in Caenorhabditis Elegans. *Arch. Pharm. Res.* **2014**, *37* (2), 245–252. https://doi.org/10.1007/s12272-013-0183-6.

(139) Mi, X.-N.; Wang, L.-F.; Hu, Y.; Pan, J.-P.; Xin, Y.-R.; Wang, J.-H.; Geng, H.-J.; Hu, S.-H.; Gao, Q.; Luo, H.-M. Methyl 3,4-Dihydroxybenzoate Enhances Resistance to Oxidative Stressors and Lifespan in C. Elegans Partially via Daf-2/Daf-16. *Int. J. Mol. Sci.* **2018**, *19* (6), 1670. https://doi.org/10.3390/ijms19061670.

(140) Nakagawa-Yagi, Y.; Sato, Y.; Matsumoto, E.; Nakatsuka, S.; Sakaki, T.; Muramatsu, Y.; Hara, T.; Aigaki, T. Pharmacological Modulation of Histone Demethylase Activity by a Small Molecule Isolated from Subcritical Water Extracts of Sasa Senanensis Leaves Prolongs the Lifespan of Drosophila Melanogaster. *BMC Complement. Altern. Med.* **2012**, *12* (1), 101. https://doi.org/10.1186/1472-6882-12-101.

(141) Surco-Laos, F.; Dueñas, M.; González-Manzano, S.; Cabello, J.; Santos-Buelga, C.; González-Paramás, A. M. Influence of Catechins and Their Methylated Metabolites on Lifespan and Resistance to Oxidative and Thermal Stress of Caenorhabditis Elegans and Epicatechin Uptake. *Food Res. Int.* **2012**, *46* (2), 514–521. https://doi.org/10.1016/j.foodres.2011.10.014.

(142) Wu, X.; Al-Amin, M.; Zhao, C.; An, F.; Wang, Y.; Huang, Q.; Teng, H.; Song, H. Catechinic Acid, a Natural Polyphenol Compound, Extends the Lifespan of Caenorhabditis Elegans via Mitophagy Pathways. *Food Funct.* **2020**, *11* (6), 5621–5634. https://doi.org/10.1039/d0fo00694g.

(143) Si, H.; Fu, Z.; Babu, P. V. A.; Zhen, W.; LeRoith, T.; Meaney, M. P.; Voelker, K. A.; Jia, Z.; Grange, R. W.; Liu, D. Dietary Epicatechin Promotes Survival of Obese Diabetic Mice and Drosophila Melanogaster. *J. Nutr.* **2011**, *141* (6), 1095–1100. https://doi.org/10.3945/jn.110.134270.

(144) Si, H.; Wang, X.; Zhang, L.; Parnell, L. D.; Ahmed, B.; LeRoith, T.; Ansah, T.-A.; Zhang, L.; Li, J.; Ordovás, J. M.; Si, H.; Liu, D.; Lai, C.-Q. Dietary Epicatechin Improves Survival and Delays Skeletal Muscle Degeneration in Aged Mice. *FASEB J.* **2019**, *33* (1), 965–977. https://doi.org/10.1096/fj.201800554RR.

(145) Xiao, Y.; Dong, J.; Yin, Z.; Wu, Q.; Zhou, Y.; Zhou, X. Procyanidin B2 Protects against D-Galactose-Induced Mimetic Aging in Mice: Metabolites and Microbiome Analysis. *Food Chem. Toxicol. Int. J. Publ. Br. Ind. Biol. Res. Assoc.* **2018**, *119*, 141–149. https://doi.org/10.1016/j.fct.2018.05.017.

(146) Xu, Q.; Fu, Q.; Li, Z.; Liu, H.; Wang, Y.; Lin, X.; He, R.; Zhang, X.; Ju, Z.; Campisi, J.; Kirkland, J. L.; Sun, Y. The Flavonoid Procyanidin C1 Has Senotherapeutic Activity and Increases Lifespan in Mice. *Nat. Metab.* **2021**. https://doi.org/10.1038/s42255-021-00491-8.

(147) Lee, E. B.; Kim, J. H.; Kim, Y. J.; Noh, Y. J.; Kim, S. J.; Hwang, I. H.; Kim, D. K. Lifespan-Extending Property of 6-Shogaol from Zingiber Officinale Roscoe in Caenorhabditis Elegans. *Arch. Pharm. Res.* **2018**, *41* (7), 743–752. https://doi.org/10.1007/s12272-018-1052-0.

(148) Wang, Z.; Zheng, P.; Chen, X.; Xie, Y.; Weston-Green, K.; Solowij, N.; Chew, Y. L.; Huang, X.-F. Cannabidiol Induces Autophagy and Improves Neuronal Health Associated with SIRT1 Mediated Longevity. *GeroScience* **2022**, *44* (3), 1505–1524. https://doi.org/10.1007/s11357-022-00559-7.

(149) Phulara, S. C.; Pandey, S.; Jha, A.; Chauhan, P. S.; Gupta, P.; Shukla, V. Hemiterpene Compound, 3,3-Dimethylallyl Alcohol Promotes Longevity and Neuroprotection in Caenorhabditis Elegans. *GeroScience* **2021**, *43* (2), 791–807. https://doi.org/10.1007/s11357-020-00241-w.

(150) Shukla, V.; Yadav, D.; Phulara, S. C.; Gupta, M. M.; Saikia, S. K.; Pandey, R. Longevity-Promoting Effects of 4-Hydroxy-E-Globularinin

in Caenorhabditis Elegans. *Free Radic. Biol. Med.* **2012**, *53* (10), 1848–1856. https://doi.org/10.1016/j.freeradbiomed.2012.08.594.

(151) Shukla, V.; Phulara, S. C.; Yadav, D.; Tiwari, S.; Kaur, S.; Gupta, M. M.; Nazir, A.; Pandey, R. Iridoid Compound 10-o-Trans-p-Coumaroylcatalpol Extends Longevity and Reduces Alpha Synuclein Aggregation in Caenorhabditis Elegans. *Cns Neurol. Disord.-Drug Targets* **2012**, *11* (8), 984–992.

(152) Seo, H. W.; Cheon, S. M.; Lee, M.-H.; Kim, H. J.; Jeon, H.; Cha, D. S. Catalpol Modulates Lifespan via DAF-16/FOXO and SKN-1/Nrf2 Activation in Caenorhabditis Elegans. *Evid. Based Complement. Alternat. Med.* **2015**, *2015*, 1–10. https://doi.org/10.1155/2015/524878.

(153) Asthana, J.; Yadav, A. K.; Pant, A.; Pandey, S.; Gupta, M. M.; Pandey, R. Specioside Ameliorates Oxidative Stress and Promotes Longevity in Caenorhabditis Elegans. *Comp. Biochem. Physiol. Part C Toxicol. Pharmacol.* **2015**, *169*, 25–34. https://doi.org/10.1016/j.cbpc.2015.01.002.

(154) Liu, Q.; Cheng, L.; Matsuura, A.; Xiang, L.; Qi, J. Gentiopicroside, a Secoiridoid Glycoside from Gentiana Rigescens Franch, Extends the Lifespan of Yeast via Inducing Mitophagy and Antioxidative Stress. *Oxid. Med. Cell. Longev.* **2020**, *2020*, 1–12. https://doi.org/10.1155/2020/9125752.

(155) Feng, S.; Zhang, C.; Chen, T.; Zhou, L.; Huang, Y.; Yuan, M.; Li, T.; Ding, C. Oleuropein Enhances Stress Eesistance and Extends Lifespan via Insulin/IGF-1 and SKN-1/Nrf2 Signaling Pathway in Caenorhabditis Elegans. *Antioxidants* **2021**, *10* (11), 1697. https://doi.org/10.3390/antiox10111697.

(156) Pant, A.; Saikia, S. K.; Shukla, V.; Asthana, J.; Akhoon, B. A.; Pandey, R. Beta-Caryophyllene Modulates Expression of Stress Response Genes and Mediates Longevity in Caenorhabditis Elegans. *Exp. Gerontol.* **2014**, *57*, 81–95. https://doi.org/10.1016/j.exger.2014.05.007.

(157) Wang, Y.; Lin, Y.; Xiang, L.; Osada, H.; Qi, J. Sesquiterpene Glucosides from Shenzhou Honey Peach Fruit Showed the Anti-Aging Activity in the Evaluation System Using Yeasts. *Biosci. Biotechnol. Biochem.* **2017**, *81* (8), 1586–1590. https://doi.org/10.1080/09168451.2017.1332978.

(158) Mohankumar, A.; Shanmugam, G.; Kalaiselvi, D.; Levenson, C.; Nivitha, S.; Thiruppathi, G.; Sundararaj, P. East Indian Sandalwood (Santalum Album L.) Oil Confers Neuroprotection and Geroprotection in Caenorhabditis Elegans via Activating SKN-1/Nrf2 Signaling Pathway. *RSC Adv.* **2018**, *8* (59), 33753–33774. https://doi.org/10.1039/C8RA05195J.

(159) García-Casas, P.; Alvarez-Illera, P.; Fonteriz, R. I.; Montero, M.; Alvarez, J. Mechanism of the Lifespan Extension Induced by Submaximal SERCA Inhibition in C. Elegans. *Mech. Ageing Dev.* **2021**, *196*, 111474. https://doi.org/10.1016/j.mad.2021.111474.

(160) Gao, L.; Zhang, R.; Lan, J.; Ning, R.; Wu, D.; Chen, D.; Zhao, W. β-Dihydroagarofuran-Type Sesquiterpenes from the Seeds of Celastrus Monospermus and Their Lifespan-Extending Effects on the Nematode Caenorhabditis Elegans. *J. Nat. Prod.* **2016**, *79* (12), 3039–3046. https://doi.org/10.1021/acs.jnatprod.6b00648.

(161) Zhang, H.; Qin, J.; Lan, X.; Zeng, W.; Zhou, J.; Huang, T.-E.; Xiao, W.-L.; Wang, Q.-Q.; Sun, S.; Su, W.; Nie, W.; Yang, S.; Yang, J.; Gao, Q.; Xiang, Y. Handelin Extends Lifespan and Healthspan of Caenorhabditis Elegans by Reducing ROS Generation and Improving Motor Function. *Biogerontology* **2022**, *23* (1), 115–128. https://doi.org/10.1007/s10522-022-09950-5.

(162) Wu, Z.; Song, L.; Liu, S. Q.; Huang, D. Tanshinones Extend Chronological Lifespan in Budding Yeast Saccharomyces Cerevisiae. *Appl. Microbiol. Biotechnol.* **2014**, *98* (20), 8617–8628. https://doi.org/10.1007/s00253-014-5890-5.

(163) Kim, J.; Kang, Y.-G.; Lee, J.; Choi, D.; Cho, Y.; Shin, J.-M.; Park, J. S.; Lee, J. H.; Kim, W. G.; Seo, D. B.; Lee, T. R.; Miyamoto, Y.; No,

K. T. The Natural Phytochemical Dehydroabietic Acid Is an Anti-Aging Reagent That Mediates the Direct Activation of SIRT1. *Mol. Cell. Endocrinol.* **2015**, *412*, 216–225. https://doi.org/10.1016/j.mce.2015.05.006.

(164) Li, P.; Wang, Z.; Lam, S. M.; Shui, G. Rebaudioside a Enhances Resistance to Oxidative Stress and Extends Lifespan and Healthspan in Caenorhabditis Elegans. *Antioxidants* **2021**, *10* (2), 262. https://doi.org/10.3390/antiox10020262.

(165) An, Y.; Zhu, J.; Wang, X.; Sun, X.; Luo, C.; Zhang, Y.; Ye, Y.; Li, X.; Abulizi, A.; Huang, Z.; Zhang, H.; Yang, B.; Xie, Z. Oridonin Delays Aging through the AKT Signaling Pathway. *Front. Pharmacol.* **2022**, *13*, 888247. https://doi.org/10.3389/fphar.2022.888247.

(166) Cho, J.; Park, Y. Kahweol, a Coffee Diterpene, Increases Lifespan via Insulin/Insulin-like Growth Factor-1 and AMP-Activated Protein Kinase Signaling Pathways in *Caenorhabditis Elegans*. *Curr. Res. FOOD Sci.* **2023**, *7*, 100618. https://doi.org/10.1016/j.crfs.2023.100618.

(167) Kim, S.-J.; Beak, S.-M.; Park, S.-K. Supplementation with Triptolide Increases Resistance to Environmental Stressors and Lifespan in C. Elegans. *J. Food Sci.* **2017**, *82* (6), 1484–1490. https://doi.org/10.1111/1750-3841.13720.

(168) Lin, C.; Zhang, X.; Su, Z.; Xiao, J.; Lv, M.; Cao, Y.; Chen, Y. Carnosol Improved Lifespan and Healthspan by Promoting Antioxidant Capacity in Caenorhabditis Elegans. *Oxid. Med. Cell. Longev.* **2019**, *2019*, 1–13. https://doi.org/10.1155/2019/5958043.

(169) Lin, C.; Zhang, X.; Xiao, J.; Zhong, Q.; Kuang, Y.; Cao, Y.; Chen, Y. Effects on Longevity Extension and Mechanism of Action of Carnosic Acid in Caenorhabditis Elegans. *Food Funct.* **2019**, *10* (3), 1398–1410. https://doi.org/10.1039/C8FO02371A.

(170) Zarse, K.; Bossecker, A.; Müller-Kuhrt, L.; Siems, K.; Hernandez, M. A.; Berendsohn, W. G.; Birringer, M.; Ristow, M. The Phytochemical Glaucarubinone Promotes Mitochondrial Metabolism, Reduces Body Fat, and Extends Lifespan of Caenorhabditis Elegans. *Horm. Metab. Res.* **2011**, *43* (04), 241–243. https://doi.org/10.1055/s-0030-1270524.

(171) Zhu, J.; Mu, X.; Zeng, J.; Xu, C.; Liu, J.; Zhang, M.; Li, C.; Chen, J.; Li, T.; Wang, Y. Ginsenoside Rg1 Prevents Cognitive Impairment and Hippocampus Senescence in a Rat Model of D-Galactose-Induced Aging. *PLoS ONE* **2014**, *9* (6), e101291. https://doi.org/10.1371/journal.pone.0101291.

(172) Li, J.; Cai, D.; Yao, X.; Zhang, Y.; Chen, L.; Jing, P.; Wang, L.; Wang, Y. Protective Effect of Ginsenoside Rg1 on Hematopoietic Stem/Progenitor Cells through Attenuating Oxidative Stress and the Wnt/β-Catenin Signaling Pathway in a Mouse Model of D-Galactose-Induced Aging. *Int. J. Mol. Sci.* **2016**, *17* (6), 849. https://doi.org/10.3390/ijms17060849.

(173) Cai, S.-Z.; Zhou, Y.; Liu, J.; Li, C.-P.; Jia, D.-Y.; Zhang, M.-S.; Wang, Y.-P. Alleviation of Ginsenoside Rg1 on Hematopoietic Homeostasis Defects Caused by Lead-Acetate. *Biomed. Pharmacother.* **2018**, *97*, 1204–1211. https://doi.org/10.1016/j.biopha.2017.10.148.

(174) Xiang, L.; Disasa, D.; Liu, Y.; Fujii, R.; Yang, M.; Wu, E.; Matsuura, A.; Qi, J. Gentirigeoside B from Gentiana Rigescens Franch Prolongs Yeast Lifespan via Inhibition of TORC1/Sch9/Rim15/Msn Signaling Pathway and Modification of Oxidative Stress and Autophagy. *ANTIOXIDANTS* **2022**, *11* (12), 2373. https://doi.org/10.3390/antiox11122373.

(175) Cao, X.; Sun, Y.; Lin, Y.; Pan, Y.; Farooq, U.; Xiang, L.; Qi, J. Antiaging of Cucurbitane Glycosides from Fruits of Momordica Charantia L. *Oxid. Med. Cell. Longev.* **2018**, *2018*, 1–10. https://doi.org/10.1155/2018/1538632.

(176) Lin, Y.; Kotakeyama, Y.; Li, J.; Pan, Y.; Matsuura, A.; Ohya, Y.; Yoshida, M.; Xiang, L.; Qi, J. Cucurbitacin B Exerts Antiaging Effects in Yeast by Regulating Autophagy and Oxidative Stress. *Oxid. Med. Cell. Longev.* **2019**, *2019*, 1–15. https://doi.org/10.1155/2019/4517091.

(177) Zhang, J.; Xue, X.; Qiao, Y.; Li, D.; Wei, Q.; Zhang, F.; Qin, X. Astragaloside IV Extends Lifespan of Caenorhabditis Elegans by Improving Age-Related Functional Declines and Triggering Antioxidant Responses. *Rejuvenation Res.* **2021**, *24* (2), 120–130.

https://doi.org/10.1089/rej.2020.2312.

(178) Fan, S.; Yan, Y.; Xia, Y.; Zhou, Z.; Luo, L.; Zhu, M.; Han, Y.; Yao, D.; Zhang, L.; Fang, M.; Peng, L.; Yu, J.; Liu, Y.; Gao, X.; Guan, H.; Li, H.; Wang, C.; Wu, X.; Zhu, H.; Cao, Y.; Huang, C. Pregnane X Receptor Agonist Nomilin Extends Lifespan and Healthspan in Preclinical Models through Detoxification Functions. *Nat. Commun.* **2023**, *14* (1), 3368. https://doi.org/10.1038/s41467-023-39118-9.

(179) Zhang, J.; Lu, L.; Zhou, L. Oleanolic Acid Activates Daf-16 to Increase Lifespan in Caenorhabditis Elegans. *Biochem. Biophys. Res. Commun.* **2015**, *468* (4), 843–849. https://doi.org/10.1016/j.bbrc.2015.11.042.

(180) Papaevgeniou, N.; Sakellari, M.; Jha, S.; Tavernarakis, N.; Holmberg, C. I.; Gonos, E. S.; Chondrogianni, N. 18α-Glycyrrhetinic Acid Proteasome Activator Decelerates Aging and Alzheimer's Disease Progression in Caenorhabditis Elegans and Neuronal Cultures. *Antioxid. Redox Signal.* **2016**, *25* (16), 855–869. https://doi.org/10.1089/ars.2015.6494.

(181) Bahrami, S. A.; Bakhtiari, N. Ursolic Acid Regulates Aging Process through Enhancing of Metabolic Sensor Proteins Level. *Biomed. Pharmacother.* **2016**, *82*, 8–14. https://doi.org/10.1016/j.biopha.2016.04.047.

(182) Staats, S.; Wagner, A. E.; Lüersen, K.; Künstner, A.; Meyer, T.; Kahns, A. K.; Derer, S.; Graspeuntner, S.; Rupp, J.; Busch, H.; Sina, C.; Ipharraguerre, I. R.; Rimbach, G. Dietary Ursolic Acid Improves Health Span and Life Span in Male Drosophila Melanogaster. *BioFactors* **2019**, *45* (2), 169–186. https://doi.org/10.1002/biof.1467.

(183) Zhu, J.; An, Y.; Wang, X.; Huang, L.; Kong, W.; Gao, M.; Wang, J.; Sun, X.; Zhu, S.; Xie, Z. The Natural Product Rotundic Acid Treats Both Aging and Obesity by Inhibiting PTP1B. *Life Med.* **2022**, *1* (3), 372–386. https://doi.org/10.1093/lifemedi/lnac044.

(184) Lee, H.-Y.; Min, K.-J. Betulinic Acid Increases the Lifespan of Drosophila Melanogaster via Sir2 and FoxO Activation. *Nutrients* **2024**, *16*

(3), 441. https://doi.org/10.3390/nu16030441.

(185) Sun, K.; Cao, S.; Pei, L.; Matsuura, A.; Xiang, L.; Qi, J. A Steroidal Saponin from Ophiopogon Japonicus Extends the Lifespan of Yeast via the Pathway Involved in SOD and UTH1. *Int. J. Mol. Sci.* **2013**, *14* (3), 4461–4475. https://doi.org/10.3390/ijms14034461.

(186) Liu, Y.; Liu, Q.; Chen, D.; Matsuura, A.; Xiang, L.; Qi, J. Inokosterone from Gentiana Rigescens Franch Extends the Longevity of Yeast and Mammalian Cells via Antioxidative Stress and Mitophagy Induction. *Antioxidants* **2022**, *11* (2), 214. https://doi.org/10.3390/antiox11020214.

(187) Smita, S. S.; Raj Sammi, S.; Laxman, T. S.; Bhatta, R. S.; Pandey, R. Shatavarin IV Elicits Lifespan Extension and Alleviates Parkinsonism in Caenorhabditis Elegans. *Free Radic. Res.* **2017**, *51* (11–12), 954–969. https://doi.org/10.1080/10715762.2017.1395419.

(188) Yang, J.; Wan, Q.-L.; Mu, Q.-Z.; Wu, C.-F.; Ding, A.-J.; Yang, Z.-L.; Qiu, M.-H.; Luo, H.-R. The Lifespan-Promoting Effect of Otophylloside B in Caenorhabditis Elegans. *Nat. Prod. Bioprospecting* **2015**, *5* (4), 177–183. https://doi.org/10.1007/s13659-015-0064-4.

(189) Yang, J.; Huang, X.-B.; Wan, Q.-L.; Ding, A.-J.; Yang, Z.-L.; Qiu, M.-H.; Sun, H.-Y.; Qi, S.-H.; Luo, H.-R. Otophylloside B Protects against Aβ Toxicity in Caenorhabditis Elegans Models of Alzheimer's Disease. *Nat. Prod. Bioprospecting* **2017**, *7* (2), 207–214. https://doi.org/10.1007/s13659-017-0122-1.

(190) Xu, J.; Guo, Y.; Sui, T.; Wang, Q.; Zhang, Y.; Zhang, R.; Wang, M.; Guan, S.; Wang, L. Molecular Mechanisms of Anti-Oxidant and Anti-Aging Effects Induced by Convallatoxin in Caenorhabditis Elegans. *Free Radic. Res.* **2017**, *51* (5), 529–544. https://doi.org/10.1080/10715762.2017.1331037.

(191) Shanmugam, G.; Mohankumar, A.; Kalaiselvi, D.; Nivitha, S.; Murugesh, E.; Shanmughavel, P.; Sundararaj, P. Diosgenin a Phytosterol

Substitute for Cholesterol, Prolongs the Lifespan and Mitigates Glucose Toxicity via DAF-16/FOXO and GST-4 in Caenorhabditis Elegans. *Biomed. Pharmacother.* **2017**, *95*, 1693–1703. https://doi.org/10.1016/j.biopha.2017.09.096.

(192) Koval, L.; Zemskaya, N.; Aliper, A.; Zhavoronkov, A.; Moskalev, A. Evaluation of the Geroprotective Effects of Withaferin A in Drosophila Melanogaster. *Aging-US* **2021**, *13* (2), 1817–1841. https://doi.org/10.18632/aging.202572.

(193) Bridi, J. C.; Barros, A. G. de A.; Sampaio, L. R.; Ferreira, J. C. D.; Antunes Soares, F. A.; Romano-Silva, M. A. Lifespan Extension Induced by Caffeine in Caenorhabditis Elegans Is Partially Dependent on Adenosine Signaling. *Front. Aging Neurosci.* **2015**, *7*. https://doi.org/10.3389/fnagi.2015.00220.

(194) Li, H.; Roxo, M.; Cheng, X.; Zhang, S.; Cheng, H.; Wink, M. Pro-Oxidant and Lifespan Extension Effects of Caffeine and Related Methylxanthines in Caenorhabditis Elegans. *Food Chem. X* **2019**, *1*, 100005. https://doi.org/10.1016/j.fochx.2019.100005.

(195) Saharia, K.; Arya, U.; Kumar, R.; Sahu, R.; Das, C. K.; Gupta, K.; Dwivedi, H.; Subramaniam, J. R. Reserpine Modulates Neurotransmitter Release to Extend Lifespan and Alleviate Age-Dependent Aβ Proteotoxicity in Caenorhabditis Elegans. *Exp. Gerontol.* **2012**, *47* (2), 188–197. https://doi.org/10.1016/j.exger.2011.12.006.

(196) Saharia, K.; Kumar, R.; Gupta, K.; Mishra, S.; Subramaniam, J. R. Reserpine Requires the D2-Type Receptor, Dop-3, and the Exoribonuclease, Eri-1, to Extend the Lifespan in C. Elegans. *J. Biosci.* **2016**, *41* (4), 689–695. https://doi.org/10.1007/s12038-016-9652-7.

(197) Fang, E. F.; Waltz, T. B.; Kassahun, H.; Lu, Q.; Kerr, J. S.; Morevati, M.; Fivenson, E. M.; Wollman, B. N.; Marosi, K.; Wilson, M. A.; Iser, W. B.; Eckley, D. M.; Zhang, Y.; Lehrmann, E.; Goldberg, I. G.; Scheibye-Knudsen, M.; Mattson, M. P.; Nilsen, H.; Bohr, V. A.;

Becker, K. G. Tomatidine Enhances Lifespan and Healthspan in C. Elegans through Mitophagy Induction via the SKN-1/Nrf2 Pathway. *Sci. Rep.* **2017**, *7* (1), 46208. https://doi.org/10.1038/srep46208.

(198) Ching, T.-T.; Chen, Y.-C.; Li, G.; Liu, J.; Xu, X. Z. S.; Hsu, A.-L. Short-Term Enhancement of Motor Neuron Synaptic Exocytosis during Early Aging Extends Lifespan in Caenorhabditis Elegans. *Exp. Biol. Med.* **2020**, *245* (17), 1552–1559. https://doi.org/10.1177/1535370220950639.

(199) Guerrero-Rubio, M. A.; Hernández-García, S.; Escribano, J.; Jiménez-Atiénzar, M.; Cabanes, J.; García-Carmona, F.; Gandía-Herrero, F. Betalain Health-Promoting Effects after Ingestion in Caenorhabditis Elegans Are Mediated by DAF-16/FOXO and SKN-1/Nrf2 Transcription Factors. *Food Chem.* **2020**, *330*, 127228. https://doi.org/10.1016/j.foodchem.2020.127228.

(200) Zeng, W.-Y.; Tan, L.; Han, C.; Zheng, Z.-Y.; Wu, G.-S.; Luo, H.-R.; Li, S.-L. Trigonelline Extends the Lifespan of C. Elegans and Delays the Progression of Age-Related Diseases by Activating AMPK, DAF-16, and HSF-1. *Oxid. Med. Cell. Longev.* **2021**, *2021*, 1–11. https://doi.org/10.1155/2021/7656834.

(201) Dubiley, T. A.; Rushkevich, Y. E.; Koshel, N. M.; Voitenko, V. P.; Vaiserman, A. M. Life Span Extension in Drosophila Melanogaster Induced by Morphine. *Biogerontology* **2011**, *12* (3), 179–184. https://doi.org/10.1007/s10522-010-9308-1.

(202) Shen, J.; Shan, J.; Zhu, X.; Yang, P.; Zhang, D.; Liang, B.; Li, M.; Zang, X.; Dai, Z. Sex Specific Effects of Capsaicin on Longevity Regulation. *Exp. Gerontol.* **2020**, *130*, 110788. https://doi.org/10.1016/j.exger.2019.110788.

(203) Song, X.; Dai, J.; Li, H.; Li, Y.; Hao, W.; Zhang, Y.; Zhang, Y.; Su, L.; Wei, H. Anti-Aging Effects Exerted by Tetramethylpyrazine Enhances Self-Renewal and Neuronal Differentiation of Rat bMSCs by Suppressing NF-kB Signaling. *Biosci. Rep.* **2019**, *39* (6),

BSR20190761. https://doi.org/10.1042/BSR20190761.

(204) Dang, Y.; An, Y.; He, J.; Huang, B.; Zhu, J.; Gao, M.; Zhang, S.; Wang, X.; Yang, B.; Xie, Z. Berberine Ameliorates Cellular Senescence and Extends the Lifespan of Mice via Regulating P16 and Cyclin Protein Expression. *Aging Cell* **2020**, *19* (1), e13060. https://doi.org/10.1111/acel.13060.

(205) Costa-Machado, L. F.; Garcia-Dominguez, E.; McIntyre, R. L.; Lopez-Aceituno, J. L.; Ballesteros-Gonzalez, Á.; Tapia-Gonzalez, A.; Fabregat-Safont, D.; Eisenberg, T.; Gomez, J.; Plaza, A.; Sierra-Ramirez, A.; Perez, M.; Villanueva-Bermejo, D.; Fornari, T.; Loza, M. I.; Herradon, G.; Hofer, S. J.; Magnes, C.; Madeo, F.; Duerr, J. S.; Pozo, O. J.; Galindo, M.-I.; del Pino, I.; Houtkooper, R. H.; Megias, D.; Viña, J.; Gomez-Cabrera, M. C.; Fernandez-Marcos, P. J. Peripheral Modulation of Antidepressant Targets MAO-B and GABAAR by Harmol Induces Mitohormesis and Delays Aging in Preclinical Models. *Nat. Commun.* **2023**, *14* (1), 2779. https://doi.org/10.1038/s41467-023-38410-y.

(206) Zhang, L.; Zhang, J.; Zhao, B.; Zhao-Wilson, X. Quinic Acid Could Be a Potential Rejuvenating Natural Compound by Improving Survival of Caenorhabditis Elegans under Deleterious Conditions. *Rejuvenation Res.* **2012**, *15* (6), 573–583. https://doi.org/10.1089/rej.2012.1342.

(207) Li, X.-H.; Li, C.-Y.; Lu, J.-M.; Tian, R.-B.; Wei, J. Allicin Ameliorates Cognitive Deficits Ageing-Induced Learning and Memory Deficits through Enhancing of Nrf2 Antioxidant Signaling Pathways. *Neurosci. Lett.* **2012**, *514* (1), 46–50. https://doi.org/10.1016/j.neulet.2012.02.054.

(208) Powolny, A. A.; Singh, S. V.; Melov, S.; Hubbard, A.; Fisher, A. L. The Garlic Constituent Diallyl Trisulfide Increases the Lifespan of C. Elegans via Skn-1 Activation. *Exp. Gerontol.* **2011**, *46* (6), 441–452. https://doi.org/10.1016/j.exger.2011.01.005.

(209) Ogawa, T.; Kodera, Y.; Hirata, D.; Blackwell, T. K.; Mizunuma, M. Natural Thioallyl Compounds Increase Oxidative Stress Resistance and Lifespan in Caenorhabditis Elegans by Modulating SKN-1/Nrf. *Sci. Rep.* **2016**, *6* (1), 21611. https://doi.org/10.1038/srep21611.

(210) Qi, Z.; Ji, H.; Le, M.; Li, H.; Wieland, A.; Bauer, S.; Liu, L.; Wink, M.; Herr, I. Sulforaphane Promotes C. Elegans Longevity and Healthspan via DAF-16/DAF-2 Insulin/IGF-1 Signaling. *Aging-US* **2021**, *13* (2), 1649–1670. https://doi.org/10.18632/aging.202512.

(211) Osorio-Paz, I.; Valle-Jiménez, X.; Brunauer, R.; Alavez, S. Vanillic Acid Improves Stress Resistance and Substantially Extends Life Span in Caenorhabditis Elegans. *J. Gerontol. Ser. A* **2023**, *78* (7), 1100–1107. https://doi.org/10.1093/gerona/glad086.

(212) Thomas, C.; Erni, R.; Wu, J. Y.; Fischer, F.; Lamers, G.; Grigolon, G.; Mitchell, S. J.; Zarse, K.; Carreira, E. M.; Ristow, M. A Naturally Occurring Polyacetylene Isolated from Carrots Promotes Health and Delays Signatures of Aging. *Nat. Commun.* **2023**, *14* (1), 8142. https://doi.org/10.1038/s41467-023-43672-7.
